# Novel synthesis of benzotriazolyl alkyl esters: an unprecedented CH_2_ insertion†

**DOI:** 10.1039/d0ra10413b

**Published:** 2021-02-17

**Authors:** Mohamed Elagawany, Lingaiah Maram, Bahaa Elgendy

**Affiliations:** Department of Pharmaceutical and Administrative Sciences, University of Health Sciences and Pharmacy St. Louis MO 63110 USA belgendy@wustl.edu; Center for Clinical Pharmacology, Washington University School of Medicine, St. Louis College of Pharmacy St. Louis MO 63110 USA; Department of Pharmaceutical Chemistry, Faculty of Pharmacy, Damanhour University Damanhour Egypt; Chemistry Department, Faculty of Science, Benha University Benha 13518 Egypt

## Abstract

We have developed a novel method for the synthesis of benzotriazolyl alkyl esters (BAEs) from *N*-acylbenzotriazoles and dichloromethane (DCM) under mild conditions. This reaction is one of few examples to show the use of DCM as a C-1 surrogate in carbon–heteroatom bond formation and to highlight the versatility of using DCM as a methylene building block.

## Abstract 2

Versatile synthesis of benzotriazolyl alkyl esters (BAEs) from *N*-acylbenzotriazoles using DCM as a methylene building block.

## Graphical abstract

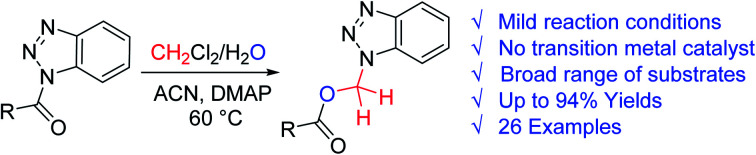

## Introduction

Benzotriazolyl alkyl esters (BAEs) are bifunctional building blocks that can be used for the construction of a myriad of multifunctional chemical compounds. The benzotriazolyl (Bt) and acyloxy groups are good leaving groups and can be selectively eliminated to provide useful scaffolds for the synthesis of important chemical compounds. For example, upon treatment of benzotriazolyl alkyl esters with organozinc reagents, one can substitute the benzotriazolyl moiety with several groups (*e.g.* alkyl, aryl, alkenyl,…*etc*) to synthesize esters.^1^ Alternatively, chemoselective reduction of benzotriazolyl alkyl esters with SmI_2_ leads to the cleavage of the acyloxy group and formation of a Bt bearing radical that undergoes cross-coupling with carbonyl compounds to form a variety of β-(benzotriazole-1-yl)alcohols.^2^

**Fig. 1:**
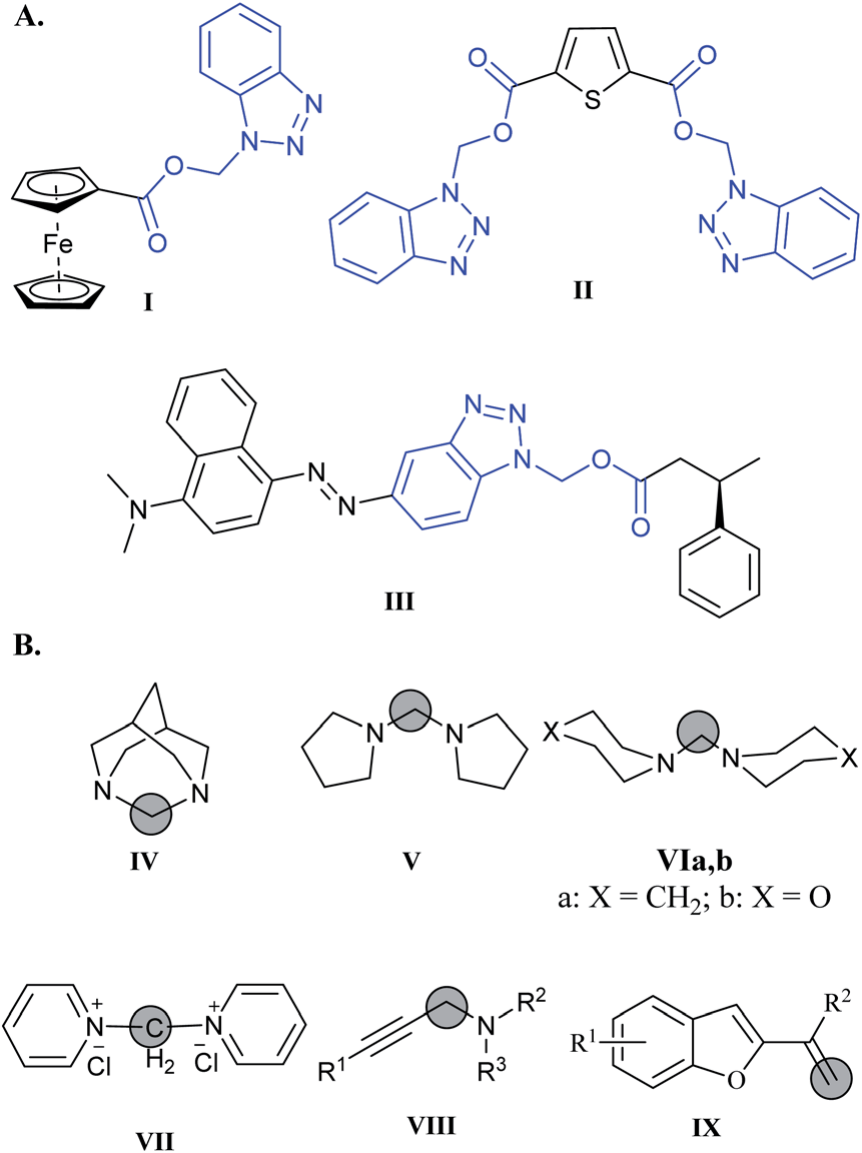
(A) Examples of multifunctional ligands based on BAE moiety (I and II) and aryl azo BAE enzyme substrates (III). (B) Compounds IV–IX synthesized using DCM as a C-1 surrogate.

Multifunctional ligands based on the BAE moiety (*i.e.*I and II, Fig. 1) were developed as nitrogen-donor ligands, and were shown to have the ability to coordinate to transition metals such as Rh(i), Ir(i), and Co(ii). These ligands stabilized their metal ions in multiple oxidation states and geometries, and improved their activity in the carbonylation of methanol. These ligands were synthesized by condensation of the hydroxymethyl-benzotriazole and carboxylic acids in DCM in the presence of DCC, DMAP, and 4-pyrrolidinopyridine.^3^

Aryl azo benzotriazolyl alkyl esters (III, Fig. 1) are used as masked enzyme substrates that can be detected by surface-enhanced resonance Raman scattering (SERRS) only after hydrolysis by the enzyme of interest. This enzymatic hydrolysis generates the dye that has strong affinity to the surface and can be detected by SERRS at very low levels and consequently allow for an enzyme assay with ultra-sensitivity for enzyme turnover monitoring.^4–6^

The most common synthetic approaches to synthesize BAEs are condensation of the hydroxymethylbenzotriazole and carboxylic acids,^3^ and the reaction of the appropriate sodium benzotriazolate with chloromethyl esters.^4^

Dichloromethane (DCM) is reported to react with strong nucleophiles,^7–9^ and bases.^10^ For example, DCM is reported to degrade at low temperature in bispidine and give 1,3-diazaadamantane hydrochloride (IV) as the major product (Fig. 1).^11^ Methylene bridged cyclic amines such as dipyrrolidylmethane (V), dipiperidylmethane (VIa), and dimorpholinylmethane (VIb) are useful chelating ligands (Fig. 1). These ligands can be synthesized easily at room temperature by stirring the corresponding bases with DCM in absence of light.^12^ Similarly, methylenebispyridinium dichloride (VII) was formed when pyridine was dissolved in DCM and left for long time.^13^ DCM was used as a C-1 surrogate in several synthetic reactions. For example, Zhang *et al.* used DCM as a C-1 building block in the synthesis of propargylamines VIII*via* CuCl catalysis.^14^ Another example was the synthesis of substituted benzofurane IX*via* a three component reaction involving DCM as a source of methylene in a C=C bond formation.^15^

In continuation of our work developing new synthetic approaches for the preparation of biologically active compounds,^16–26^ we discovered and developed a new methodology for the synthesis of BAEs *via* CH_2_ insertion using DCM. The new method is general, convenient and has a broad scope of applicability to various substrates.

## Results and discussion

**Scheme 1:**
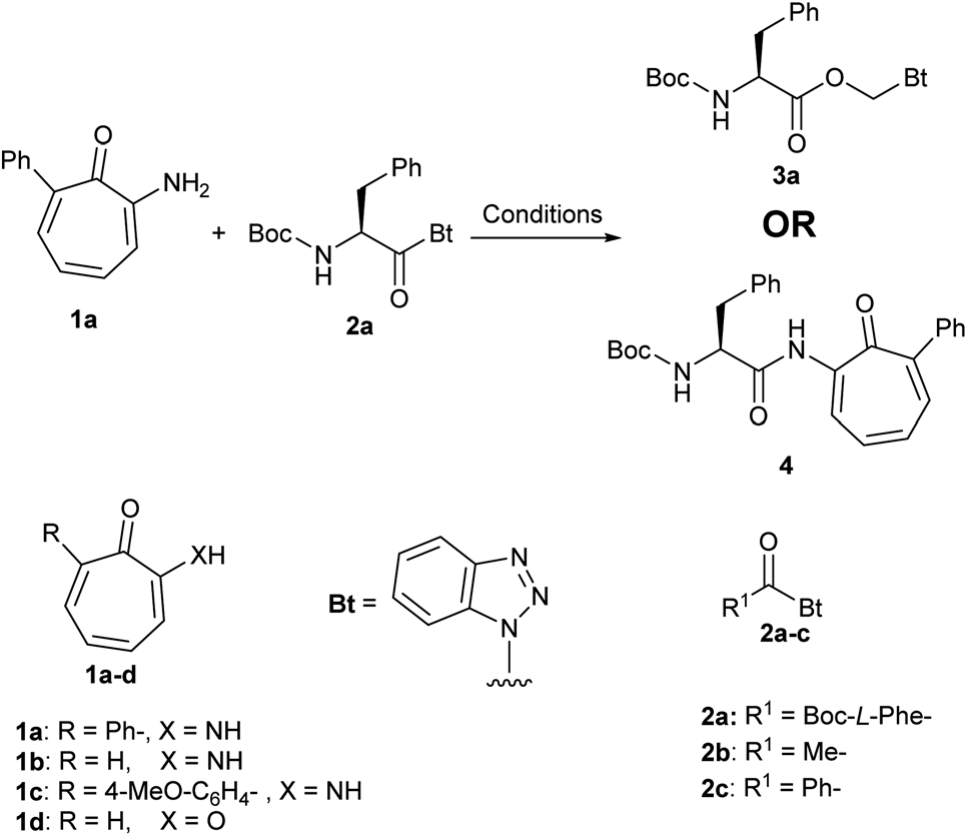
Reaction of aminotropones (1a–c) and tropone (1d) with acyl benzotriazoles 2a–c.

One of our ongoing research programs is to develop tropolones as antibacterial agents.^27,28^ While developing aminoacid conjugates of aminotropones 1a, we used acyl benzotriazole as an acylating reagent. Aminotropones were reported to couple with similar acylating reagents such as acid chlorides under conventional reaction conditions.^29,30^ When we reacted aminotropone 1a with acyl benzotriazole 2a in acetonitrile (ACN) in presence of Hunig's base (Scheme 1) according to standard protocol of coupling using *N*-acyl benzotriazole as an acylating reagent,^16,22^ we observed no progress after one hour of stirring at room temperature, and the starting materials were largely insoluble. Therefore, we added few drops of dichloromethane (DCM) as a co-solvent to enhance the solubility of the starting materials. The reaction mixture became homogeneous, but no reaction took place. We repeated the reaction using one equivalent of DMAP, but no change was observed at room temperature. Heating the reaction mixture at 60 °C for 12 h led to consumption of the acyl benzotriazole and formation of a new product 3a, which was isolated and purified (Scheme 1). Aminotropone 1a was isolated as a side product. The molecular weight of the expected product 4 is 444, but LC/MS of 3a showed a molecular weight of 397 (M + H) (*i.e.* 48 mass unit difference from 4 and 31 mass unit difference from 2a). Comparing ^1^H NMR spectrum of 3a to spectrum of 2a showed two additional protons at 6.50 and 7.70 ppm. The two protons appeared as douplets with the same coupling constant (*i.e. J* = 11.3 Hz). The ^13^C NMR spectrum showed an extra aliphatic carbon at 68.2 ppm. We ran a series of 2D NMR to identify the product and we found that the two unexpected protons at 6.50 and 7.70 ppm are attached to the same aliphatic carbon at 68.2 ppm and the new product has the Boc-l-Phe moiety.

When we tried the coupling of aminotropone 1a with either acyl benzotriazole (2b) and/or benzoyl benzotriazole (2c) (Scheme 1) under the same reaction conditions (ACN/DCM, DMAP/60 °C), we obtained products that has the same mass differences from substrates and expected products that was observed in the case of Boc-l-Phe–Bt (*i.e.* 31 and 48). Coupling of other aminotropones (*i.e.*1b and 1c), and tropone 1d led to the formation of similar unexpected products with 48 mass unit difference than the expected products. In both cases aminotropones (1b and 1c) and tropone (1d) were isolated as side products. We repeated the reaction under same conditions but avoided using DCM as a co-solvent and we obtained the expected amide and benzotriazole as a side product.

**Fig. 2:**
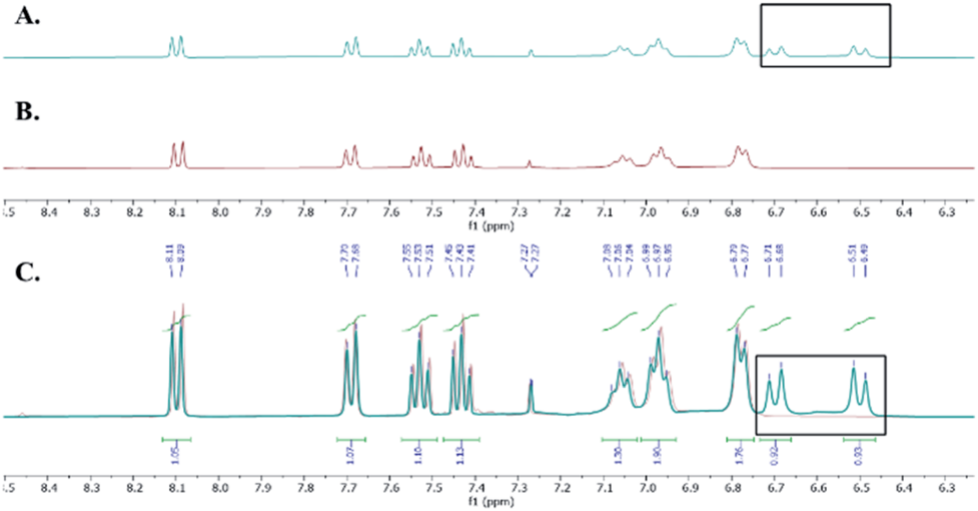
(A) ^1^H NMR of 3a. (B) ^1^H NMR of 3b. (C) Overlay of ^1^H NMR of 3a (green) and 3b (red).

We hypothesized that the extra CH_2_ was generated from DCM and to test this hypothesis, we ran the reaction in deuterated DCM. We obtained product 3b and by analyzing and comparing the ^1^H NMR spectra of 3a and 3b, we found that the two spectra were superimposed with the extra two protons disappeared in case of deuterated DCM, which suggests that these protons are generated from DCM (Fig. 2). The molecular formula of 3a (C_21_H_24_N_4_O_4_Na^+^) was determined by HRESIMS data (*m*/*z* 419.1687 [M + Na]^+^, calcd 419.1690). Similarly, the molecular formula of 3b (C_21_H_22_D_2_N_4_O_4_Na^+^) was determined by HRESIMS data (*m*/*z* 421.1812 [M + Na]^+^, calcd 421.1815).

**Fig. 3:**
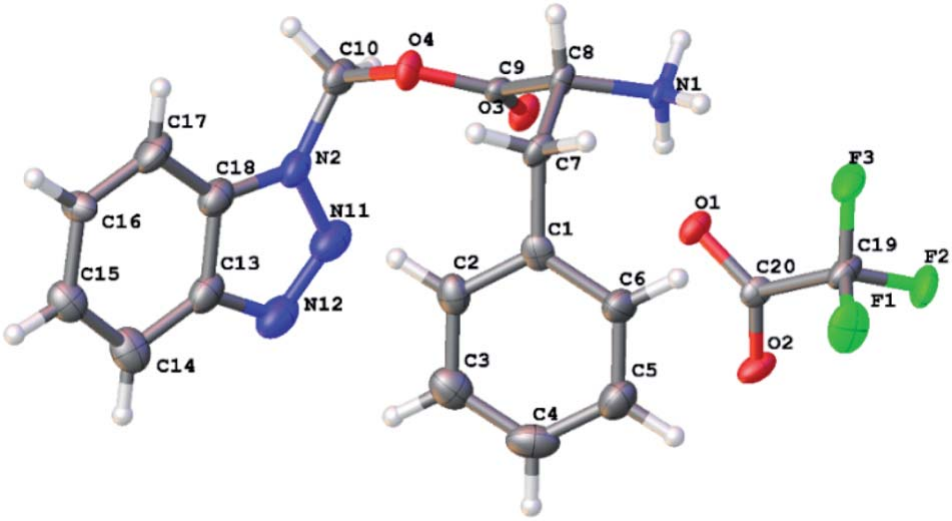
An ORTEP representation of the X-ray crystallography structure of BAE 3c with the ellipsoid shown at 50% contour % probability level.

Finally, we were able to determine the structure of the product to be benzotriazolyl alkyl ester using X-ray crystallography. Deprotection of the Boc group of 3a using TFA in DCM led to the formation of 3c that was suitable to obtain a single crystal X-ray structure (Fig. 3). The molecular formula of 3c (C_16_H_16_N_4_O_2_Na^+^) was determined by HRESIMS data (*m*/*z* 319.1165 [M + Na]^+^, calcd 319.1165).

**Fig. 4:**
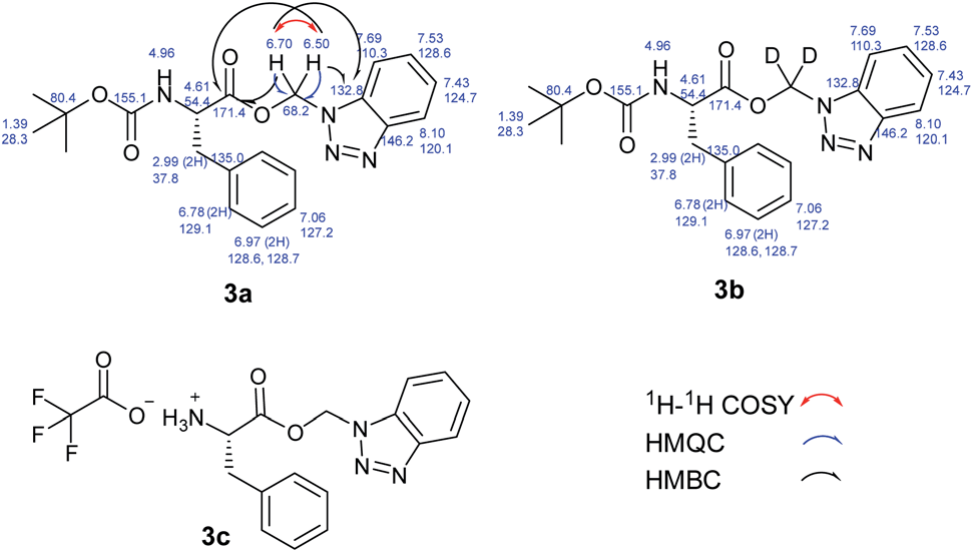
^1^H and ^13^C Chemical shift assignments of 3a and 3b; and chemical structure of 3c.

The structures of 3a and 3b were further confirmed following a thorough analysis of 2D NMR data. All the protons and carbons were assigned based on COSY, HSQC, and HMBC correlations (Fig. 4).

To determine if aminotropone derivatives contribute to the observed transformation, we carried out the reaction in absence of aminotropone and obtained the same product in the same reaction time and with the same yield, which indicates that aminotropone did not play a role in this transformation.

We hypothesize that the mechanism of this reaction takes place *via*: (i) base-catalysed hydrolysis of *N*-acyl benzotriazole.^31^ (ii). *In situ* formation of chloromethyl benzotriazole through reaction of benzotriazole with DCM in presence of base (benzotriazole was reported to react with DCM in DMF in presence of sodium hydride at reflux conditions).^32^ (iii) The base facilitates the formation of acyloxy anion that reacted with chloromethyl benzotriazole to form the desired BAE.^33^

To explore the scope of this reaction, we started first by optimizing the reaction conditions using synthesis of (1*H*-benzo[*d*][1,2,3]triazol-1-yl)methyl benzoate (5a) as a template reaction (Table 1). Screening numerous solvents revealed that ACN and DCM were the best solvents and gave the desired product 5a in 93% yield (Table 1, entries 1 and 2). Although using DCM only as a solvent and reagent afforded the product in high yield (93%), ACN is preferred to minimize the hazardous of using larger quantity of DCM and to design a reaction that is green in nature. Replacing ACN with DMF or THF led to the formation of the product in lower yields (*i.e.* 55 and 63%, respectively) (Table 1, entries 3–4). No product was observed when ACN was replaced by 1,4-dioxane, toluene, water, or ethanol (Table 1, entries 5–8).

**Table 1:** Optimization of conditions^a^

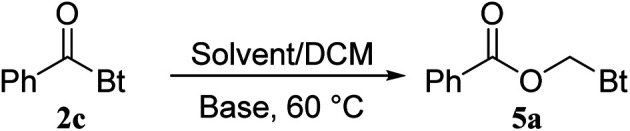			
Entry	Solvent	Base	Yield (%)
1	Acetonitrile	DMAP (1 equiv.)	93
2	DCM	DMAP (1 equiv.)	93
3	DMF	DMAP (1 equiv.)	55
4	THF	DMAP (1 equiv.)	63
5	1,4-Dioxane	DMAP (1 equiv.)	N.R.
6	Toluene	DMAP (1 equiv.)	N.R.
7	Water	DMAP (1 equiv.)	N.R.
8	Ethanol	DMAP (1 equiv.)	N.R.
9	Acetonitrile	DIPEA (1–3 equiv.)	N.R.
10	Acetonitrile	TEA (1–3 equiv.)	N.R.
11	Acetonitrile	DBU (1 equiv.)	65
12	Acetonitrile	KOH (1 equiv.)	N.R.
13	Acetonitrile	*t*-BuOK (1 equiv.)	N.R.
14^b^	Acetonitrile	DMAP (1 equiv.)	N.R.
15^b^^,^^c^	Acetonitrile	DMAP (1 equiv.)	20
16^b^^,^^d^	Acetonitrile	DMAP (1 equiv.)	50
17^b^^,^^e^	Acetonitrile	DMAP (1 equiv.)	95
18^b^^,^^f^	Acetonitrile	DMAP (1 equiv.)	95

a Conditions: 2c (0.3 mmol), base (0.3 mmol), in solvent (4 mL) and DCM (1 mL) at 60 °C for 5 h.

b Anhydrous solvents and inert argon conditions.

c H_2_O (2.0 equiv.).

d H_2_O (5.0 equiv.).

e H_2_O (10.0 equiv.).

f H_2_O (20.0 equiv.).

To identify the optimum base for the reaction, we screened several other bases in addition to DMAP in the best identified solvent (*i.e.* ACN) (Table 1, entries 9–13). DMAP was found to give the best result (Table 1, entry 1). No reaction was observed when DIPEA or TEA were used, and the starting material remained intact. When DBU was used, the product 5a was obtained in 65% (entry 11). The use of KOH or potassium-*tert*-butoxide led to hydrolysis of the starting *N*-acyl benzotriazole 2c (entries 12 and 13).

To further confirm the proposed mechanism and verify that water is the source of oxygen in the formed product, we have conducted a series of controlled experiments with anhydrous solvents under inert conditions and using water as a reagent to test if water is the source of oxygen and if this is the case to determine how many equivalents of water are needed to ensure sufficient reactivity (Table 1, entries 14–18). Compound 5a was not formed using anhydrous ACN and DCM in absence of water under argon and starting materials remained intact (entry 14). When we added H_2_O to the reaction, compound 5a was formed and the best yield was achieved using 10.0 equivalents of H_2_O. Increasing H_2_O to 20 equivalents did not improve the yield further and no hydrolysis of starting materials was observed. Therefore, H_2_O is the source of oxygen and 10 equiv. is the optimum ratio to obtain the desired product in the model reaction.

**Scheme 2:**
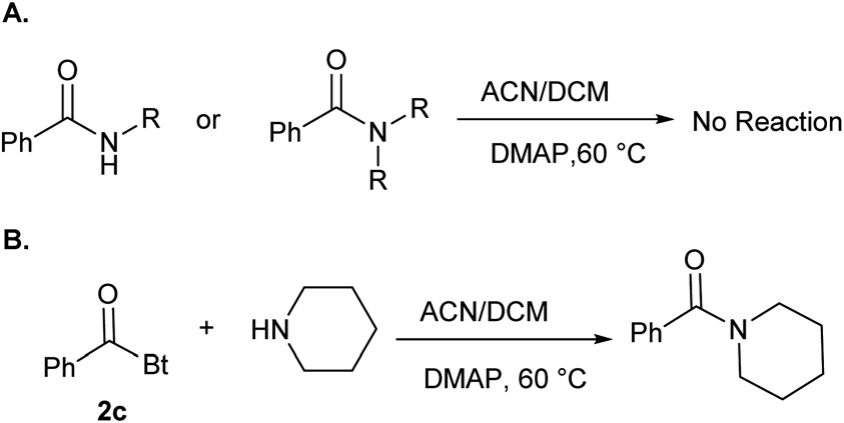
(A) Reaction of *N*-phenylbenzamide and *N*,*N*-diphenylbenzamide with DCM. (B) Competition experiment using piperidine as a nucleophile.

To investigate if this kind of CH_2_ insertion could take place with other *N*-acyl derivatives, we used *N*-phenylbenzamide and *N*,*N*-diphenylbenzamide as substrates (Scheme 2A). No reaction was observed in both cases under the optimized reaction conditions. Finally, we ran a competition experiment in the presence of piperidine as a strong nucleophile. The amide product was obtained exclusively with no insertion taking place at all (Scheme 2B). This observation illustrates that in presence of strong nucleophile, the coupling is the preferred pathway, which is usually the case in coupling reactions involving *N*-acyl benzotriazoles. Insertion of CH_2_ was the favored pathway in case of aminotropones (Scheme 1) due to their weak nucleophilicity.

**Fig. 5:**
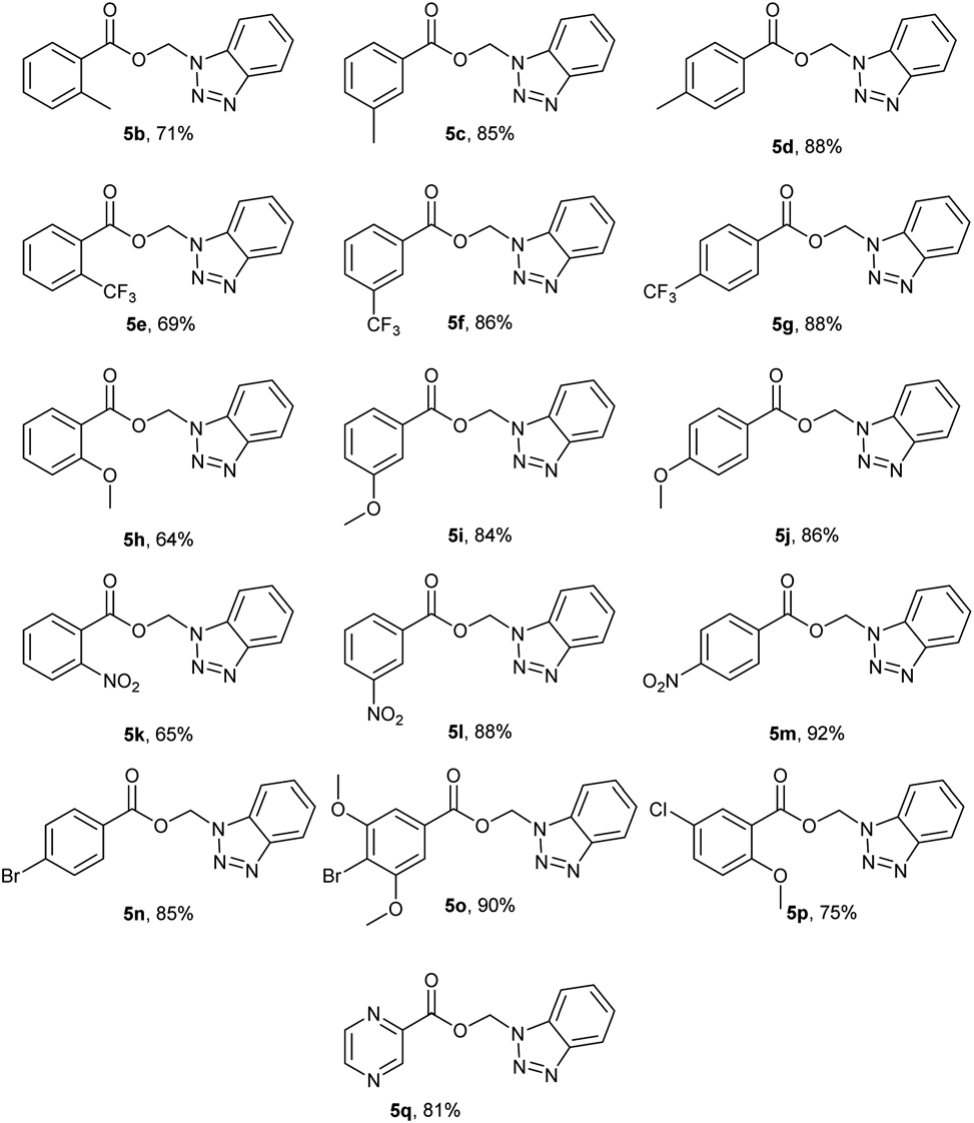
Benzotriazolyl alkyl esters (BAEs) 5a–n.

To further explore the generality of this reaction, we screened various aromatic acyl benzotriazoles (2d–s) bearing electron donating groups, electron withdrawing groups, and substituted at *o*-, *m*-, and *p*- positions of the phenyl ring. We have also used 1*H*-benzotriazol-1-yl-2-pyrazinyl methanone as a representative of heterocyclic acyl benzotriazoles. The desired BAEs (5a–n) were obtained in all cases in moderate to excellent yields (63–93%) (Fig. 5). It is worth mentioning that compounds 5b,e,h,k were obtained in low yields (*i.e.* <10%) using our standard reaction conditions. Yields were improved significantly when we used DBU as a base under the same reaction conditions (*i.e.* 64–71%). Compounds 5b,e,h,k possess *ortho*-substituents at the phenyl ring and we hypothesize that these compounds have better steric complementarity with DBU over DMAP. Compound 5p was the only exception because it was obtained in good yield (75%) using DMAP. Although 5p is analogous to 5h (both 5p and 5h possess the same *ortho*-methoxy substituent), the *meta*-chloro substituent in 5p most likely contributed to the observed difference in reactivity.

**Fig. 6:**
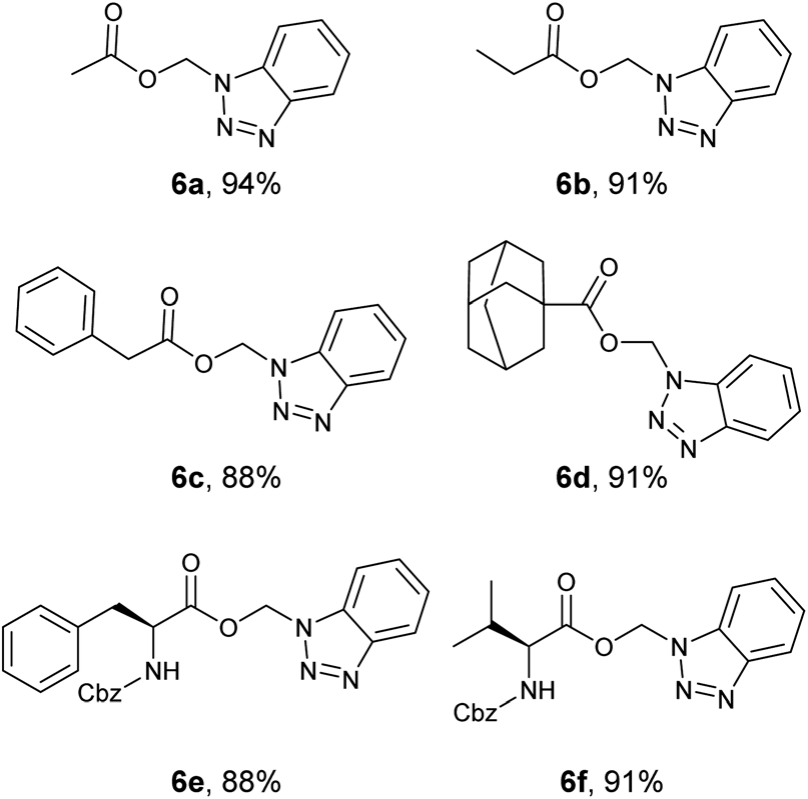
Benzotriazolyl alkyl esters (BAEs) 6a–f.

Aliphatic BAEs (6a–d) were synthesized from their corresponding *N*-acylbenzotriazoles (Fig. 6). Acetate, propionate, and phenylacetate derivatives were synthesized in 88–93% yield. The adamantane-1-carboxylate benzotriazolyl alkyl ester (6d) was synthesized in 91% yield. Moreover, we extended our new methodology to synthesize amino acid derivatives of benzotriazolyl alkyl ester. Boc-l-Phe- benzotriazolyl alkyl ester (3a, Fig. 4) was synthesized in 94% yield. This compound was deprotected in TFA/DCM (1 : 1) and gave the free aminoacid derivative 3c in quantitative yield (Fig. 4). Using deuterated DCM afforded compound 3b in 91% yield (Fig. 4). This clearly showed that our method was compatible with Boc protecting group. The method was also compatible with Cbz protecting group and we synthesized both Cbz-l-Phe- and Cbz-l-Val- benzotriazolyl alkyl ester (6e and 6f) in 88 and 91% yield, respectively.

## Conclusions

In summary, we have developed a simple and practical method for the synthesis of benzotriazolyl alkyl esters (BAEs), which are bi-functional building blocks useful for the synthesis of numerous multifunctional compounds. The structure of BAE 3c was confirmed using X-ray crystallography. We used DCM as a C-1 surrogate in a carbon–heteroatom bond formation under metal-free conditions that highlights the versatility of using DCM as a building block. The new method is convenient and requires simple operation, applicable to broad scope of substrates, and products are obtained in high yields.

## Conflicts of interest

There are no conflicts to declare.

## Supplementary Material

RA-011-D0RA10413B-s001

RA-011-D0RA10413B-s002
