# Supplementary material for: Novel synthesis of benzotriazolyl alkyl esters: an unprecedented CH2 insertion
Source: RSC Adv. 2021 Feb 17;11(13):7564–9. doi: 10.1039/d0ra10413b (PMC8694985; doi:10.1039/d0ra10413b)

Electronic Supplementary Material (ESI) for RSC Advances.
This journal is © The Royal Society of Chemistry 2021

Novel synthesis of benzotriazolyl alkyl esters: An Unprecedented CH_2 Insertion

Mohamed Elagawany,[a,b,c] Lingaiah Maram,[a,b] Bahaa Elgendy*,[a,b,d]

[a]*Department of Pharmaceutical and Administrative Sciences, University of Health Sciences and Pharmacy, St. Louis, MO 63110, USA*
[b]*Center for Clinical Pharmacology, Washington University School of Medicine and St. Louis College of Pharmacy, St. Louis, MO 63110, USA*
[c]*Department of Pharmaceutical Chemistry, Faculty of Pharmacy, Damanhour University, Damanhour, Egypt*
[d]*Chemistry Department, Faculty of Science, Benha University, Benha 13518, Egypt*

Table of Contents

Materials and Methods **S2**

Experimental Procedures **S2**

^{1}H and ^{13}C NMR spectra……………………………………………………… S11-S71

Crystallographic Details of X-ray single crystal data of 3c……………………… S72

Materials and Methods

All materials were purchased from commercial suppliers and used without further purification. The purities of the final compounds were characterized by high-performance liquid chromatography (LC/MS) using a gradient elution program (Ascentis Express Peptide C18 column, acetonitrile/water 5/95/95/5, 5 min, 0.05% formic acid) and UV detection (254 nM). The purities of final compounds were 95% or greater. NMR spectra was recorded on a Bruker NMR 400 MHz Avance III spectrometer operating at 400 MHz for ^{1}H NMR and 100 MHz for ^{13}C NMR. Chemical shifts are given in part per million (ppm) relative to tetramethylsilane (TMS), coupling constants *J* are given in Hertz. HPLC-HRMS analyses were performed on reverse phase gradient using Agilent (Santa Clara, CA) 1200 series binary pump (G1312B), waters XTerra MS C_{18} (3.5 um; 2.1 x 150 mm) + Phenomenex C_{18} security guard column (2 x 4 mm) using 0.2% acetic acid in H_2O/methanol as mobile phases; wavelength = 254 nm; and mass spectrometry was done with 6220 Agilent (Santa Clara, CA) TOF in electrospray ionization (ESI) mode with positive and negative method in both Profile and Centroid mode.

General Procedure for the Synthesis of Benzotriazolyl Alkyl Esters

Method 1: (compounds 3a, 3b, 5a, 5c, 5d, 5f, 5g, 5i, 5j, 5l-5q, and 6a-6f):

DMAP (0.3 mmol, 1 equiv.) was added to a stirred solution of Acyl-Bt (0.3 mmol, 1 equiv.) in acetonitrile (4 mL) and DCM (1 mL). The reaction mixture was stirred at 60 ˚C for 5 h. After completion of the reaction determined by TLC, the solvent was evaporated, and the product was purified by flash chromatography using ethyl acetate: Hexanes (1:4) to obtain the desired product in pure form.

Method 2: (compounds 5b, 5e, 5h, and 5k)

DBU (0.3 mmol, 1 equiv.) was added to a stirred solution of Acyl-Bt (0.3 mmol, 1 equiv.) in acetonitrile (4 mL) and DCM (1 mL). The reaction mixture was stirred at 60 ˚C for 5 h. After completion of the reaction determined by TLC, the solvent was evaporated, and the product was purified by flash chromatography using ethyl acetate: Hexanes (1:4) to obtain the desired product in pure form.

(1*H*-Benzo[*d*][1,2,3]triazol-1-yl)methyl (*tert*-butoxycarbonyl)-*L*-phenylalaninate (3a)

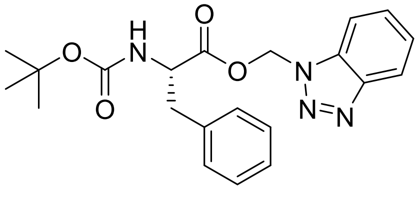

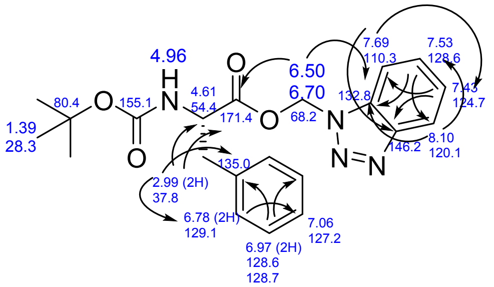

Colorless oil (113 mg, 0.29 mmol, 94%); 1H NMR (400 MHz, CDCl3) δ 8.10 (d, *J* = 8.3 Hz, 1H), 7.69 (d, *J* = 8.3 Hz, 1H), 7.53 (t, *J* = 7.7 Hz, 1H), 7.43 (t, *J* = 7.6 Hz, 1H), 7.06 (t, *J* = 7.5 Hz, 1H), 6.97 (t, *J* = 7.5 Hz, 2H), 6.78 (d, *J* = 7.5 Hz, 2H), 6.70 (d, *J* = 11.3 Hz, 1H), 6.50 (d, *J* = 11.3 Hz, 1H), 4.96 (d, *J* = 8.2 Hz, 1H), 4.61 (q, *J* = 6.8 Hz, 1H), 2.99 (d, *J* = 6.0 Hz, 2H), 1.39 (s, 9H), 1.29

– 1.21 (m, 1H); 13C NMR (100 MHz, CDCl3) δ 171.4, 155.1, 146.2, 135.0, 132.8, 129.0, 128.6, 128.6, 127.2, 124.7, 120.1, 110.3, 80.4, 68.2, 54.4, 37.8, 28.3. δ. HRMS m/z for C21H24N4O4 [M+Na]+. Calcd 419.168976, found 419.168746.

(1*H*-Benzo[*d*][1,2,3]triazol-1-yl)methyl-*d₂* (*tert*-butoxycarbonyl)-*L*-phenylalaninate (3b)

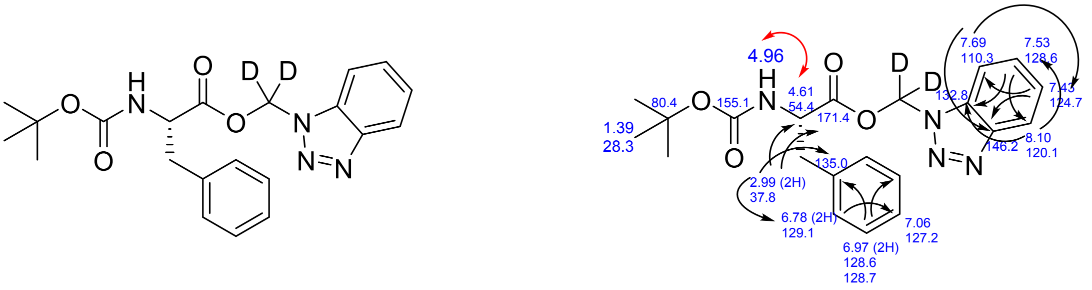

Colorless oil (109 mg, 0.27 mmol, 91%); ^{1}H NMR (400 MHz, $CDCl_3$) δ 8.09 (d, J = 8.3 Hz, 1H), 7.69 (d, J = 8.3 Hz, 1H), 7.57 – 7.49 (m, 1H), 7.45 – 7.39 (m, 1H), 7.06 (t, J = 7.4 Hz, 1H), 6.96 (t, J = 7.5 Hz, 2H), 6.78 (d, J = 7.4 Hz, 2H), 4.99 (d, J = 8.2 Hz, 1H), 4.61 (q, J = 6.7 Hz, 1H), 2.99 (d, J = 6.1 Hz, 2H), 1.39 (s, 9H); ^{13}C NMR (101 MHz, $CDCl_3$) δ 171.4, 155.1, 146.1, 135.0, 132.8, 129.0, 128.6, 128.5, 127.1, 124.7, 120.1, 110.3, 80.3, 54.4, 37.8, 31.6, 28.3. HRMS *m/z* for $C_{21}H_{22}D_2N_4O_4$ $[M+Na]^+$. Calcd 421.181530, found 421.181242.

(*S*)-1-((1*H*-Benzo[*d*][1,2,3]triazol-1-yl)methoxy)-1-oxo-3-phenylpropan-2-aminium trifluoroacetate (3c)

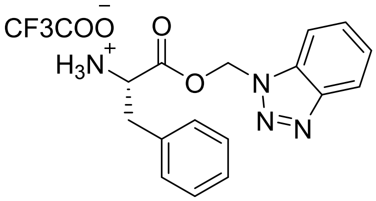

Compound **3a** was dissolved in a mixture of DCM and TFA (1:1) and stirred for 1h. The solvent was evaporated and the residue was crystallized from ethanol: ether (1:5) to obtain the triflate salt as a white needles (99%); HRMS *m/z* for $C_{16}H_{16}N_4O_2$ $[M+Na]^+$. Calcd 319.116547, found 319.116482.

(1*H*-Benzo[*d*][1,2,3]triazol-1-yl)methyl benzoate (5a)[1]

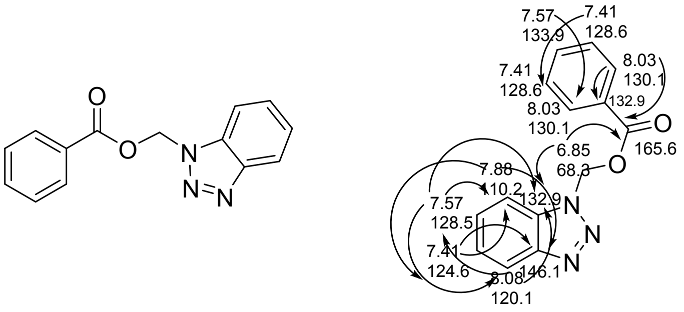

White microcrystals (71 mg, 0.28 mmol, 93%); mp 78-80 ˚C. ^{1}H NMR (400 MHz, $CDCl_3$) δ; ^{13}C NMR (100 MHz, $CDCl_3$) δ. 8.08 (d, J = 8.3 Hz, 1H), 8.06 – 8.00 (m, 2H), 7.88 (d, J = 8.3 Hz,

1H), 7.60 – 7.53 (m, 2H), 7.41 (t, J = 7.7 Hz, 3H), 6.85 (s, 2H); ^{13}C NMR (100 MHz, $CDCl_3$) δ 165.6, 146.1, 133.9, 132.9, 130.1, 128.6, 128.5, 128.4, 128.2, 124.6, 120.1, 110.2, 68.3.

(1*H*-Benzo[*d*][1,2,3]triazol-1-yl)methyl 2-methylbenzoate (5b)

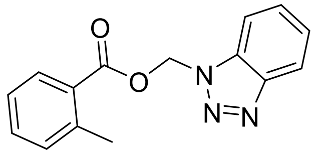

Off white microcrystals (57 mg, 0.21 mmol, 71%); mp 85-87 ˚C. ^{1}H NMR (400 MHz, $CDCl_3$) δ 8.09 (d, J = 8.8 Hz, 1H), 7.89 (t, 2H), 7.57 (t, 1H), 7.41 (q, J = 7.5 Hz, 2H), 7.25 – 7.17 (m, 2H), 6.82 (s, 2H), 2.58 (s, 3H); ^{13}C NMR (100 MHz, $CDCl_3$) δ 166.0, 146.2, 141.3, 133.0, 132.8, 131.9, 131.1, 128.4, 127.4, 125.9, 124.5, 120.1, 110.1, 68.1, 21.9. HRMS *m/z* for $C_{15}H_{13}N_3O_2$ [M+Na]$^+$. Calcd 290.089998, found 290.089904.

(1*H*-Benzo[*d*][1,2,3]triazol-1-yl)methyl 3-methylbenzoate (5c)

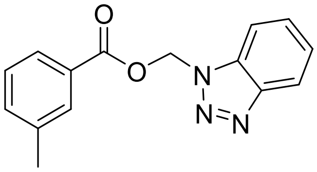

White microcrystals (68 mg, 0.25 mmol, 85%); mp 93-94 ˚C. ^{1}H NMR (400 MHz, $CDCl_3$) δ 8.08 (d, J = 8.4 Hz, 1H), 7.91 – 7.81 (m, 3H), 7.57 (t, J = 8.4 Hz, 1H), 7.44 – 7.35 (m, 2H), 7.31 (dd, J = 7.6, 1.5 Hz, 1H), 6.84 (s, 2H), 2.36 (s, 3H); ^{13}C NMR (100 MHz, $CDCl_3$) δ 165.8, 146.1, 138.5, 134.7, 132.9, 130.6, 128.5, 128.3, 127.2, 124.6, 120.1, 110.2, 68.2, 21.2. HRMS *m/z* for $C_{15}H_{13}N_3O_2$ [M+Na]$^+$. Calcd 290.089998, found 290.089938.

(1*H*-Benzo[*d*][1,2,3]triazol-1-yl)methyl 4-methylbenzoate (5d)

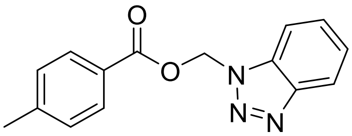

Off white microcrystals (70 mg, 0.26 mmol, 88%); mp 84-86 ˚C. ^{1}H NMR (400 MHz, $CDCl_3$) δ 8.08 (d, J = 9.1 Hz, 1H), 7.92 (d, J = 8.1 Hz, 2H), 7.88 (d, J = 7.8 Hz, 1H), 7.56 (t, 1H), 7.41 (t, 1H), 7.21 (d, J = 8.1 Hz, 2H), 6.83 (s, 2H), 2.38 (s, 3H); ^{13}C NMR (100 MHz, $CDCl_3$) δ 165.6, 146.2, 144.9, 132.9, 130.1, 129.3, 128.4, 125.6, 124.5, 120.1, 110.2, 68.2, 21.7. HRMS *m/z* for $C_{15}H_{13}N_3O_2$ [M+Na]$^+$. Calcd 290.089998, found 290.089941.

(1*H*-Benzo[*d*][1,2,3]triazol-1-yl)methyl 2-(trifluoromethyl)benzoate (5e)

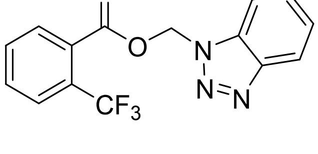

Colorless oil (69%); ^{1}H NMR (66 mg, 0.21 mmol, 400 MHz, $CDCl_3$) δ 8.10 (d, J = 8.4 Hz, 1H), 7.84 (d, J = 8.3 Hz, 1H), 7.78 (d, J = 7.0 Hz, 1H), 7.73 (d, J = 7.3 Hz, 1H), 7.64 – 7.56 (m, 3H), 7.46 – 7.41 (m, 1H), 6.85 (s, 2H); ^{13}C NMR (100 MHz, $CDCl_3$) δ 165.4, 146.2, 132.8, 132.2, 131.9, 131.9, 130.7, 128.6, 127.0, 124.7, 120.2, 110.0, 68.8. HRMS *m/z* for $C_{15}H_{10}F_3N_3O_2$ $[M+Na]^+$. Calcd 344.061732, found 344.061538.

(1*H*-Benzo[*d*][1,2,3]triazol-1-yl)methyl 3-(trifluoromethyl)benzoate (5f)

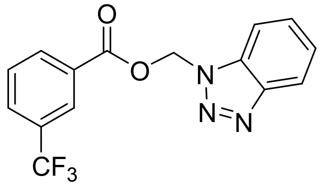

White microcrystals (82 mg, 0.26 mmol, 86%); mp 78-80 ˚C. ^{1}H NMR (400 MHz, $CDCl_3$) δ 8.22 (d, J = 7.9 Hz, 1H), 8.10 (d, J = 9.1 Hz, 1H), 7.88 (d, J = 9.0 Hz, 1H), 7.83 (d, J = 8.4 Hz, 1H), 7.63 – 7.55 (m, 2H), 7.44 (t, J = 9.2 Hz, 1H), 6.88 (s, 2H); ^{13}C NMR (100 MHz, $CDCl_3$) δ 164.4, 146.2, 133.2, 132.9, 130.5, 130.5, 129.3, 129.3, 128.7, 127.1, 124.7, 120.2, 110.0, 68.5. HRMS *m/z* for $C_{15}H_{10}F_3N_3O_2$ $[M+Na]^+$. Calcd 344.061732, found 344.061717.

(1*H*-Benzo[*d*][1,2,3]triazol-1-yl)methyl 4-(trifluoromethyl)benzoate (5g)

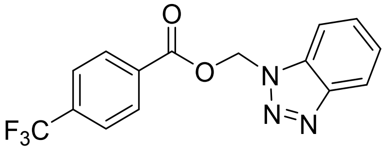

White microcrystals (84 mg, 0.26 mmol, 88%); mp 88-89 ˚C. ^{1}H NMR (400 MHz, $CDCl_3$) δ 8.16 (d, J = 8.2 Hz, 2H), 8.11 (d, J = 8.4 Hz, 1H), 7.87 (d, J = 8.9 Hz, 1H), 7.70 (d, J = 8.2 Hz, 2H), 7.60 (t, J = 7.7 Hz, 1H), 7.44 (t, J = 7.7 Hz, 1H), 6.88 (s, 2H); ^{13}C NMR (100 MHz, $CDCl_3$) δ 164.5, 146.2, 135.2, 132.8, 131.6, 130.5, 128.7, 125.7, 124.7, 122.0, 120.3, 110.0, 77.4. HRMS *m/z* for $C_{15}H_{10}F_3N_3O_2$ $[M+Na]^+$. Calcd 344.061732, found 344.061521.

(1*H*-Benzo[*d*][1,2,3]triazol-1-yl)methyl 2-methoxybenzoate (5h)

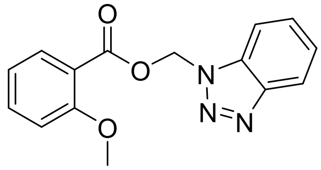

White microcrystals (54 mg, 0.19 mmol, 64%); mp 91-92 ˚C. ^{1}H NMR (400 MHz, $CDCl_3$) δ 8.08 (d, J = 8.4 Hz, 1H), 7.90 (d, J = 9.1 Hz, 1H), 7.80 (d, J = 7.8 Hz, 1H), 7.56 (t, J = 7.2 Hz, 1H), 7.50 – 7.46 (m, 1H), 7.41 (t, J = 8.1 Hz, 1H), 7.00 – 6.90 (m, 3H), 6.81 (s, 2H), 3.85 (s, 3H); ^{13}C NMR (100 MHz, $CDCl_3$) δ 164.6, 160.0, 146.1, 134.8, 134.4, 132.9, 132.2, 128.4, 124.5, 120.2, 120.0, 112.1, 110.4, 68.1, 56.0. HRMS *m/z* for $C_{15}H_{13}N_3O_3$ $[M+Na]^+$. Calcd 306.084912, found 306.084901.

(1*H*-Benzo[*d*][1,2,3]triazol-1-yl)methyl 3-methoxybenzoate (5i)

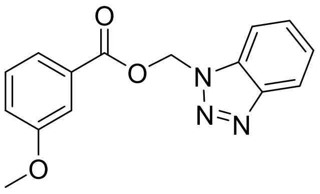

White microcrystals (71 mg, 0.25 mmol, 84%); mp 122-123 ˚C.^{1}H NMR (400 MHz, $CDCl_3$) δ 8.09 (d, J = 8.4 Hz, 1H), 7.89 (d, J = 8.4 Hz, 1H), 7.64 (d, J = 7.7 Hz, 1H), 7.61 – 7.52 (m, 2H), 7.42 (t, J = 8.2 Hz, 1H), 7.37 – 7.28 (m, 1H), 7.12 (d, J = 7.4 Hz, 1H), 6.84 (s, 2H), 3.82 (s, 3H); ^{13}C NMR (100 MHz, $CDCl_3$) δ 165.5, 159.6, 146.2, 132.9, 129.6, 128.5, 124.6, 122.5, 120.6, 120.1, 114.3, 110.2, 68.4, 55.5. HRMS *m/z* for $C_{15}H_{13}N_3O_3$ [M+Na]$^+$. Calcd 306.084912, found 306.084901.

(1*H*-Benzo[*d*][1,2,3]triazol-1-yl)methyl 4-methoxybenzoate (5j)

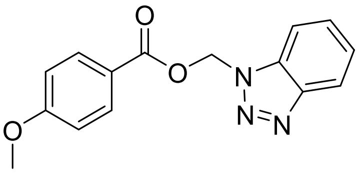

Colorless prisms (73 mg, 0.26 mmol, 86%); mp 129-130 ˚C. ^{1}H NMR (400 MHz, $CDCl_3$) δ 8.08 (d, J = 8.4 Hz, 1H), 7.99 (d, J = 9.0 Hz, 2H), 7.89 (d, J = 8.4 Hz, 1H), 7.56 (t, J = 7.7 Hz, 1H), 7.41 (d, J = 7.7 Hz, 1H), 6.89 (d, J = 9.0 Hz, 2H), 6.82 (s, 2H), 3.84 (s, 3H); ^{13}C NMR (100 MHz, $CDCl_3$) δ 165.2, 164.2, 146.1, 132.9, 132.2, 128.4, 124.5, 120.6, 120.0, 113.9, 110.3, 68.1, 55.5. HRMS *m/z* for $C_{15}H_{13}N_3O_3$ [M+Na]$^+$. Calcd 306.084912, found 306.084792.

(1*H*-Benzo[*d*][1,2,3]triazol-1-yl)methyl 2-nitrobenzoate (5k)

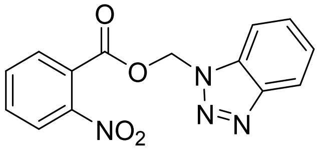

White microcrystals (58 mg, 0.19 mmol, 65%); mp 108-110 ˚C.^{1}H NMR (400 MHz, $CDCl_3$) δ 8.10 (d, J = 8.4 Hz, 1H), 7.96 – 7.92 (m, 1H), 7.85 (d, J = 8.3 Hz, 1H), 7.70 – 7.60 (m, 4H), 7.46 – 7.43 (m, 1H), 6.83 (s, 2H); ^{13}C NMR (100 MHz, $CDCl_3$) δ 164.6, 147.9, 146.2, 133.3, 132.9, 132.5, 129.9, 128.7, 126.2, 124.8, 124.2, 120.1, 109.9, 69.4. HRMS *m/z* for $C_{14}H_{10}N_4O_4$ [M+Na]$^+$. Calcd 321.059426, found 321.059241.

(1*H*-Benzo[*d*][1,2,3]triazol-1-yl)methyl 3-nitrobenzoate (5l)

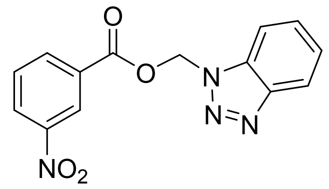

Colorless needles (79 mg, 0.27 mmol, 88%); mp 118-120 ˚C. ^1H NMR (400 MHz, $CDCl_3$) δ 8.86 (s, 1H), 8.46 – 8.34 (m, 2H), 8.09 (d, J = 8.4 Hz, 1H), 7.89 (d, J = 8.4 Hz, 1H), 7.71 – 7.58 (m, 2H), 7.47 – 7.42 (m, 1H), 6.91 (s, 2H); ^{13}C NMR (100 MHz, $CDCl_3$) δ 163.7, 148.3, 146.1, 135.6, 132.8, 130.2, 130.0, 128.7, 128.3, 125.0, 124.8, 120.2, 110.0, 68.7. HRMS *m/z* for $C_{14}H_{10}N_4O_4$ $[M+Na]^+$. Calcd 321.059426, found 321.059290.

(1*H*-Benzo[*d*][1,2,3]triazol-1-yl)methyl 4-nitrobenzoate (5m)

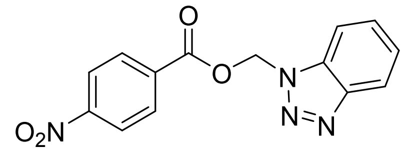

White microcrystals (82 mg, 0.28 mmol, 92%); mp 124-126 ˚C. ^1H NMR (400 MHz, $CDCl_3$) δ 8.31 – 8.20 (m, 4H), 8.11 (d, J = 9.1 Hz, 1H), 7.87 (d, J = 9.1 Hz, 1H), 7.61 (t, J = 7.7 Hz, 1H), 7.45 (t, J = 7.7 Hz, 1H), 6.89 (s, 2H); ^{13}C NMR (100 MHz, $CDCl_3$) δ 163.9, 151.1, 146.2, 133.7, 132.8, 131.3, 128.8, 124.8, 123.7, 120.3, 109.9, 68.8. HRMS *m/z* for $C_{14}H_{10}N_4O_4$ $[M+Na]^+$. Calcd 321.059426, found 321.059083.

(1*H*-Benzo[*d*][1,2,3]triazol-1-yl)methyl 4-bromobenzoate (5n)

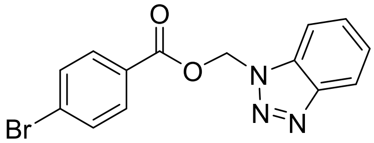

White microcrystals (88 mg, 0.27 mmol, 89%); mp 117-118 ˚C. ^1H NMR (400 MHz, $CDCl_3$) δ 8.09 (d, J = 8.4 Hz, 1H), 7.91 – 7.84 (m, 3H), 7.61 – 7.54 (m, 3H), 7.43 (t, J = 7.7 Hz, 1H), 6.84 (s, 2H); ^{13}C NMR (100 MHz, $CDCl_3$) δ 164.9, 146.2, 132.8, 132.0, 131.5, 129.3, 128.6, 127.3, 124.7, 120.2, 110.1, 68.4. HRMS *m/z* for $C_{14}H_{10}BrN_3O_2$ $[M+Na]^+$. Calcd 353.984860, found 353.984711.

(1*H*-Benzo[*d*][1,2,3]triazol-1-yl)methyl 4-bromo-3,5-dimethoxybenzoate (5o)

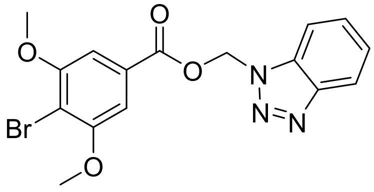

White microcrystals (105 mg, 0.27 mmol, 90%); mp 197-198 ˚C. ^{1}H NMR (400 MHz, $CDCl_3$) δ 8.10 (d, J = 7.9 Hz, 1H), 7.90 (d, J = 8.4 Hz, 1H), 7.59 (t, J = 7.4 Hz, 1H), 7.44 (t, J = 7.4 Hz, 1H), 7.23 (s, 2H), 6.85 (s, 2H), 3.92 (s, 6H); ^{13}C NMR (100 MHz, $CDCl_3$) δ 165.2, 157.2, 146.2, 132.9, 128.6, 128.3, 124.7, 120.2, 110.2, 108.1, 105.8, 68.4, 56.7. HRMS m/z for $C_{16}H_{14}BrN_3O_4$ $[M+Na]^+$. Calcd 414.005990, found 414.005887.

(1*H*-Benzo[*d*][1,2,3]triazol-1-yl)methyl 5-chloro-2-methoxybenzoate (5p)

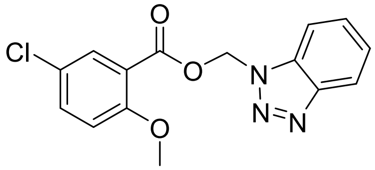

White microcrystals (71 mg, 0.22 mmol, 75%); mp 135-137 ˚C. ^{1}H NMR (400 MHz, $CDCl_3$) δ 8.09 (d, J = 8.4 Hz, 1H), 7.88 (d, J = 8.3 Hz, 1H), 7.76 (d, J = 2.7 Hz, 1H), 7.57 (t, J = 8.0 Hz, 1H), 7.46 – 7.39 (m, 2H), 6.89 (d, J = 8.9 Hz, 1H), 6.80 (s, 2H), 3.83 (s, 3H); ^{13}C NMR (100 MHz, $CDCl_3$) δ 163.6, 158.5, 146.1, 134.4, 132.9, 131.7, 128.5, 125.3, 124.6, 120.1, 118.9, 113.5, 110.3, 68.3, 56.3. HRMS m/z for $C_{15}H_{12}ClN_3O_3$ $[M+Na]^+$. Calcd 340.045940, found 340.045806.

(1*H*-Benzo[*d*][1,2,3]triazol-1-yl)methyl pyrazine-2-carboxylate (5q)

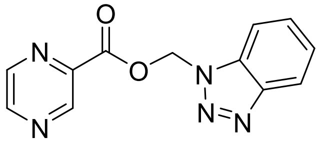

White microcrystals (62 mg, 0.24 mmol, 81%); mp 116-118 °C.^{1}H NMR (400 MHz, $CDCl_3$) δ 9.30 (s, 1H), 8.78 (d, J = 2.3 Hz, 1H), 8.76 – 8.69 (m, 1H), 8.07 (d, J = 8.4 Hz, 1H), 7.91 (d, J = 8.4 Hz, 1H), 7.58 (t, J = 7.7 Hz, 1H), 7.42 (t, J = 7.7 Hz, 1H), 6.96 (s, 2H); ^{13}C NMR (100 MHz, $CDCl_3$) δ 163.1, 148.4, 146.6, 146.1, 144.7, 142.0, 132.8, 128.7, 124.7, 120.1, 110.1, 69.0. HRMS m/z for $C_{12}H_9N_5O_2$ $[M+Na]^+$. Calcd 278.064846, found 278.064691.

(1*H*-Benzo[*d*][1,2,3]triazol-1-yl)methyl acetate (6a)[1]

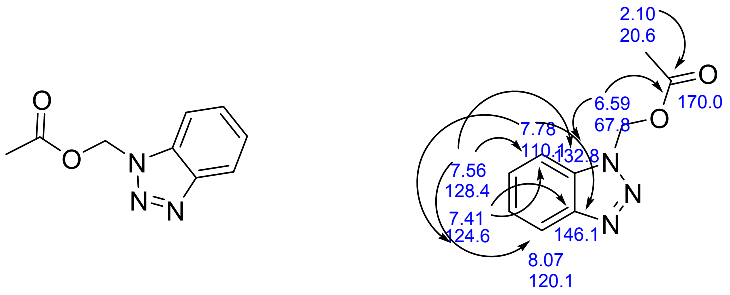

Colorless oil (54 mg, 0.28 mmol, 94%). ^{1}H NMR (400 MHz, $CDCl_3$) δ ; ^{13}C NMR (100 MHz, $CDCl_3$) δ. δ 8.07 (dd, J = 8.4, 1.1 Hz, 1H), 7.78 (dd, J = 8.4, 1.2 Hz, 1H), 7.60 – 7.51 (m, 1H), 7.46 – 7.37 (m, 1H), 6.59 (s, 2H), 2.10 (s, 3H); ^{13}C NMR (100 MHz, $CDCl_3$) δ 170.0, 146.1, 132.8, 128.4, 124.6, 120.1, 110.1, 67.8, 20.6.

(1H-Benzo[d][1,2,3]triazol-1-yl)methyl propionate (6b)

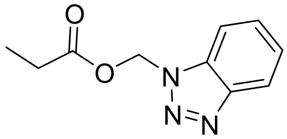

Colorless oil (56 mg, 0.27 mmol, 91%). ^{1}H NMR (400 MHz, $CDCl_3$) δ 8.08 (d, J = 8.4 Hz, 1H), 7.78 (d, J = 8.4 Hz, 1H), 7.56 (t, J = 8.1 Hz, 1H), 7.42 (t, J = 8.1 Hz, 1H), 6.60 (s, 2H), 2.38 (q, J = 7.5 Hz, 2H), 1.13 (t, J = 7.5 Hz, 3H); ^{13}C NMR (100 MHz, $CDCl_3$) δ 173.6, 146.1, 132.8, 128.4, 124.5, 120.1, 110.1, 67.7, 27.2, 8.7. HRMS *m/z* for $C_{10}H_{11}ClN_3O_2$ [M+Na]$^+$. Calcd 228.074348, found 228.074270.

(1*H*-Benzo[*d*][1,2,3]triazol-1-yl)methyl 2-phenylacetate (6c)

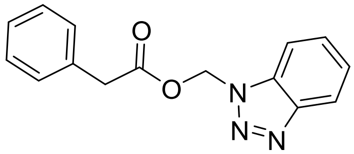

Colorless oil (70 mg, 0.26 mmol, 88%). ^{1}H NMR (400 MHz, $CDCl_3$) δ s; ^{13}C NMR (100 MHz, $CDCl_3$) δ 170.8, 146.1, 132.7, 129.2, 128.7, 128.4, 127.5, 124.6, 120.1, 110.0, 68.2, 40.8.. HRMS *m/z* for $C_{15}H_{13}N_3O_2$ [M+Na]$^+$. Calcd 290.089998, found 290.089896.

(1*H*-Benzo[*d*][1,2,3]triazol-1-yl)methyl adamantane-1-carboxylate (6d)

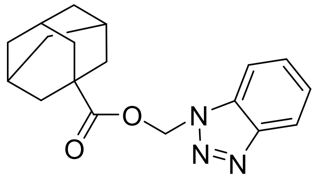

White microcrystals (85 mg, 0.27 mmol, 91%); mp 123-125 ˚C. ^{1}H NMR (400 MHz, $CDCl_3$) δ 8.08 (d, J = 8.4 Hz, 1H), 7.75 (d, J = 8.4 Hz, 1H), 7.55 (t, J = 6.7 Hz, 1H), 7.41 (t, J = 6.7 Hz, 1H), 6.59 (s, 2H), 2.04 – 1.94 (m, 3H), 1.89 – 1.81 (m, 6H), 1.73 – 1.60 (m, 6H); ^{13}C NMR (100 MHz, $CDCl_3$) δ 176.7, 146.1, 132.7, 128.4, 124.5, 120.1, 110.0, 68.0, 40.9, 38.4, 36.2, 27.7. HRMS *m/z* for $C_{18}H_{21}N_3O_2$ [M+Na]$^+$. Calcd 334.152598, found 334.152533.

(1*H*-Benzo[*d*][1,2,3]triazol-1-yl)methyl ((benzyloxy)carbonyl)-*L*-phenylalaninate (6e)

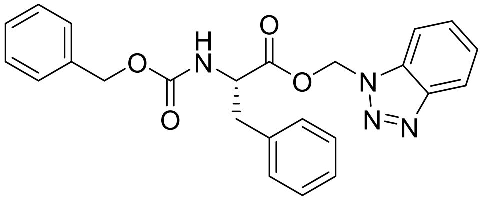

Colorless oil (114 mg, 0.27 mmol, 88%); ^{1}H NMR (400 MHz, $CDCl_3$) δ 8.11 (d, J = 8.4 Hz, 1H), 7.67 (d, J = 8.5 Hz, 1H), 7.58 – 7.26 (m, 7H), 7.12 – 6.89 (m, 3H), 6.87 – 6.62 (m, 3H), 6.51 (d, J = 11.2 Hz, 1H), 5.18 (d, J = 7.4 Hz, 1H), 5.07 (s, 2H), 4.79 – 4.64 (m, 1H), 3.02 (d, J = 5.7 Hz,

2H); ^{13}C NMR (100 MHz, $CDCl_3$) δ 170.9, 155.6, 146.1, 136.0, 134.7, 132.8, 128.9, 128.6, 128.6, 128.5, 128.3, 128.1, 127.1, 124.7, 120.1, 110.1, 68.2, 67.2, 54.7, 37.7. HRMS *m/z* for $C_{24}H_{22}N_4O_4$ $[M+Na]^+$. Calcd 453.153326, found 453.152977.

(1*H*-Benzo[*d*][1,2,3]triazol-1-yl)methyl ((benzyloxy)carbonyl)-*L*-valinate (6f)

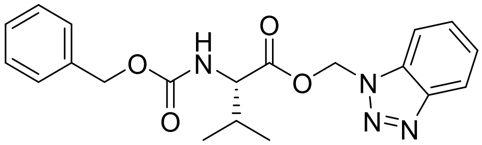

Colorless oil (104 mg, 0.27 mmol, 91%); ^1H NMR (400 MHz, $CDCl_3$) δ 8.07 (d, J = 8.3 Hz, 1H), 7.74 (d, J = 8.3 Hz, 1H), 7.55 (t, J = 7.6 Hz, 1H), 7.41 (d, J = 7.6 Hz, 1H), 7.33 (q, J = 8.8 Hz, 4H), 6.70 (d, J = 11.2 Hz, 1H), 6.54 (d, J = 11.1 Hz, 1H), 5.32 (d, J = 8.8 Hz, 1H), 5.07 (s, 2H), 4.32 (dd, J = 8.8, 4.8 Hz, 1H), 2.14 – 2.00 (m, 1H), 0.82 (d, J = 6.8 Hz, 3H), 0.70 (d, J = 6.8 Hz, 3H); ^{13}C NMR (100 MHz, $CDCl_3$) δ 171.4, 156.2, 146.0, 136.0, 132.6, 128.5, 128.3, 128.1, 124.7, 120.2, 109.9, 68.3, 67.2, 58.9, 30.9, 18.8. HRMS *m/z* for $C_{20}H_{22}N_4O_4$ $[M+Na]^+$. Calcd 405.153326, found 405.153055.

References:

1 A. R. Katritzky, W. Kuzmierkiewicz, B. Rachwal, S. Rachwal and J. Thomson, The chemistry of N-substituted benzotriazoles. Part 5. Reactions of benzotriazole with aldehydes and thionyl chloride—formation of (benzotriazol-1-yl)-1-chloroalkanes and bis(benzotriazolyl)alkanes, *J. Chem. Soc., Perkin Trans. 1*, 1987, 811–817.

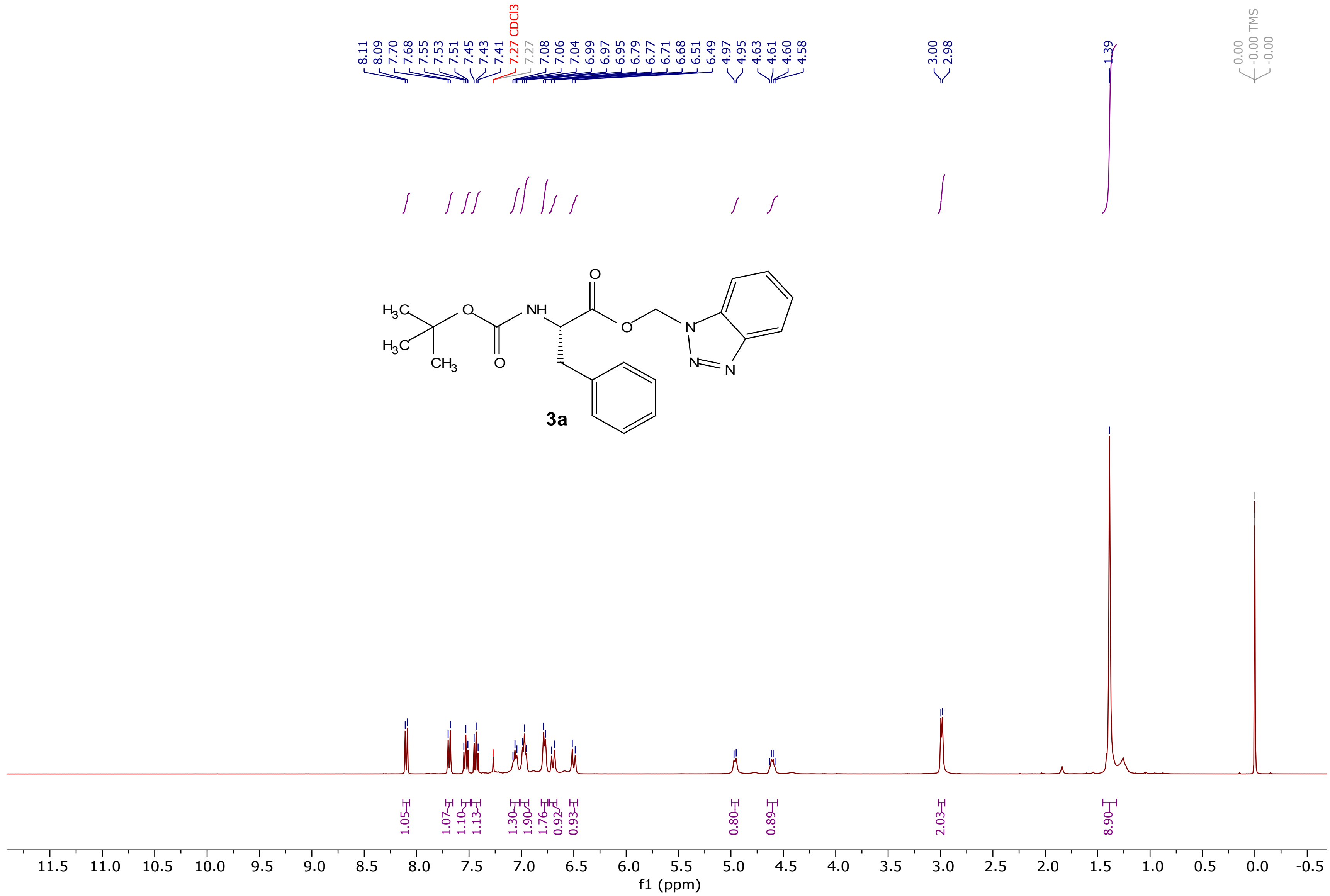

Figure S1. ^{1}H NMR spectra of **3a**.

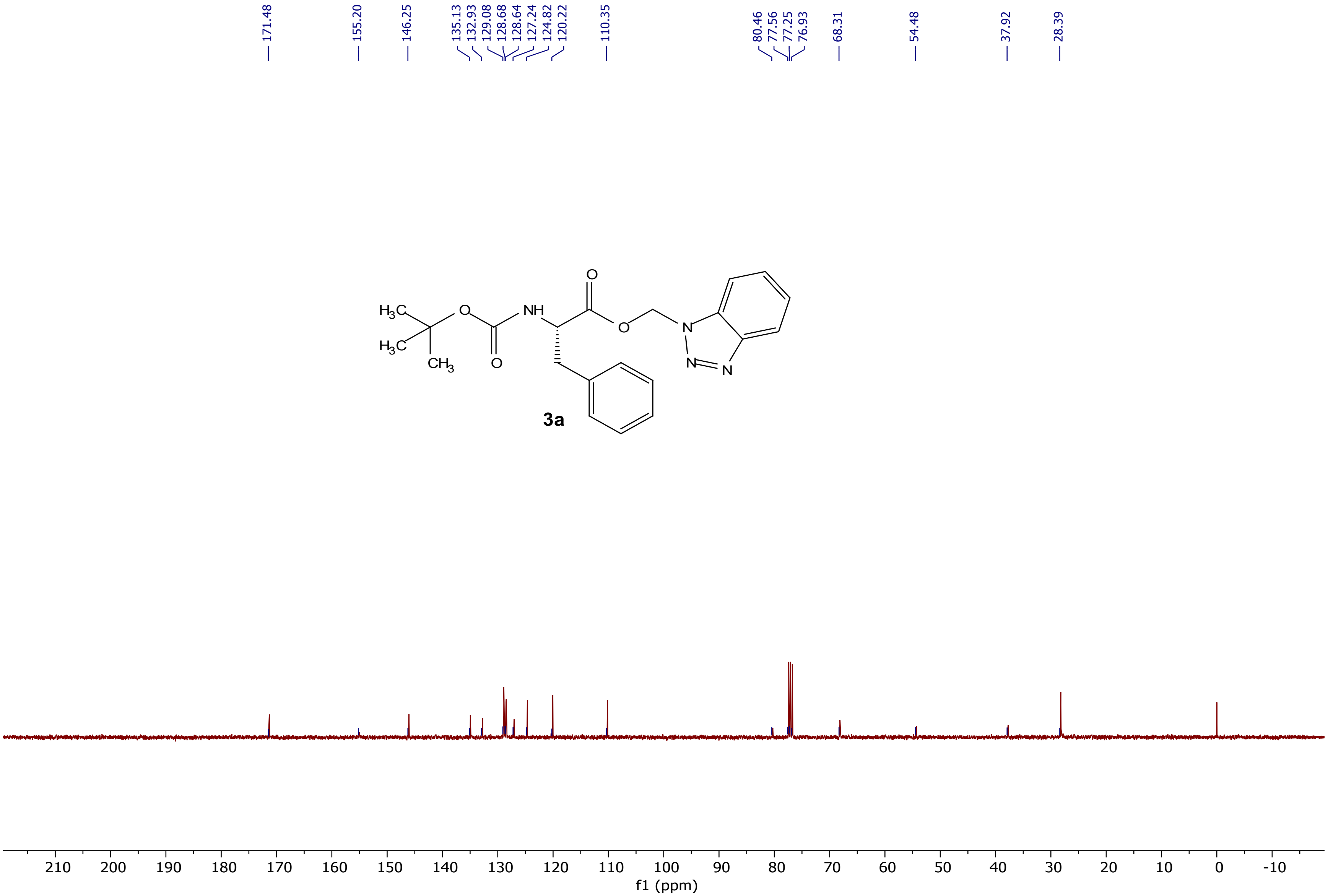

Figure S2. ^{13}C NMR spectra of **3a**.

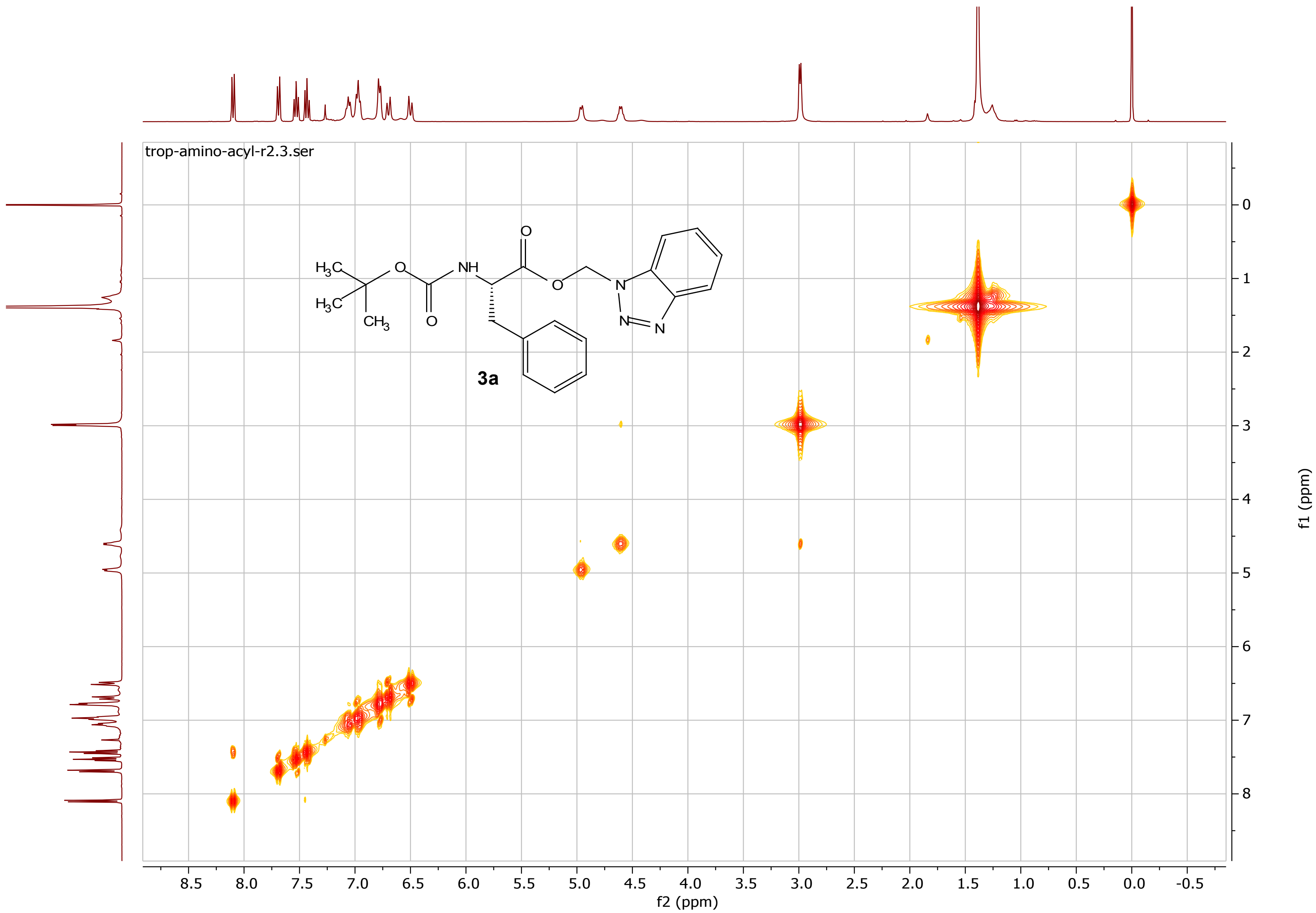

Figure S3. ^{1}H-^{1}H COSY of **3a**.

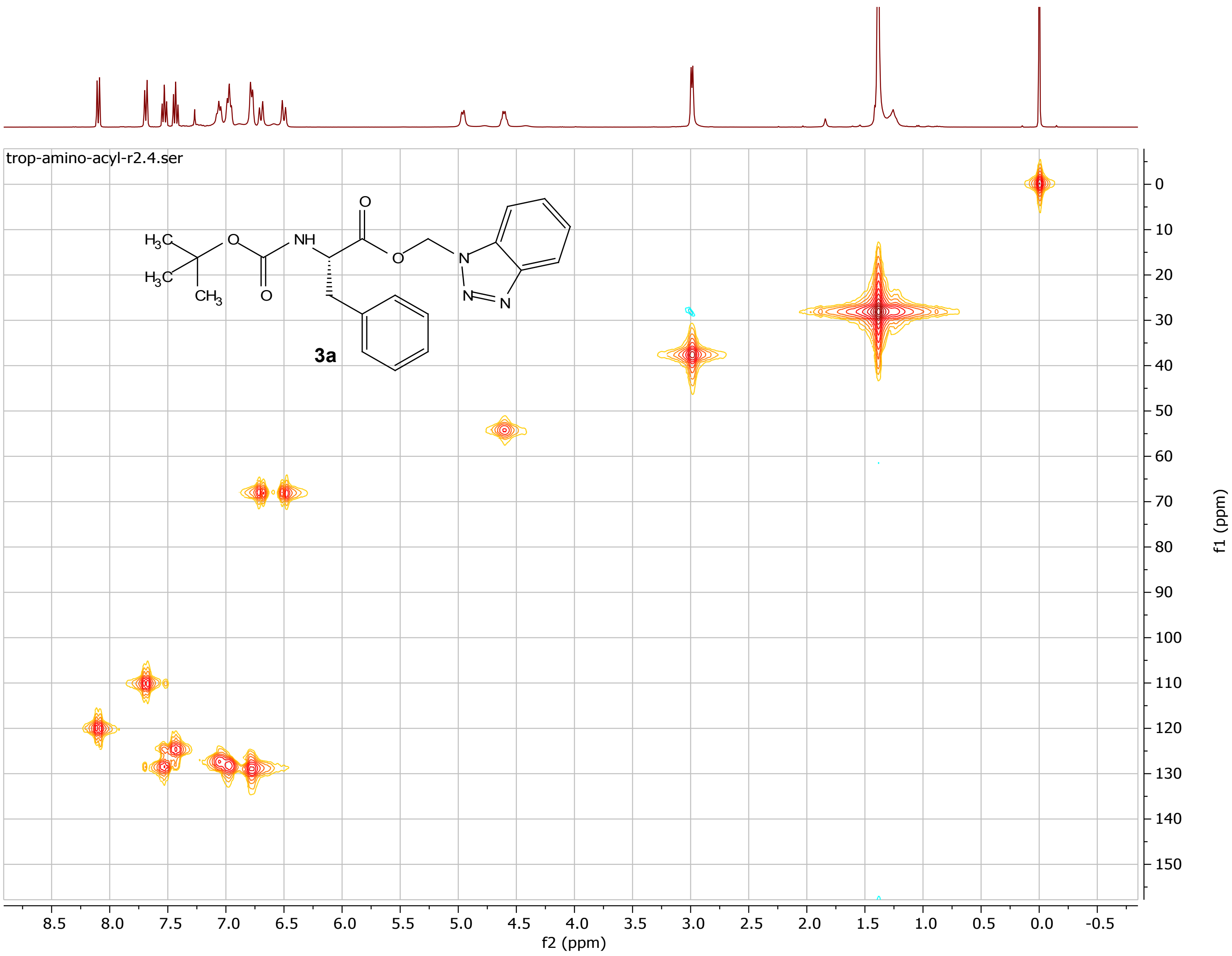

Figure S4. ^{1}H-^{13}C HMQC of **3a**.

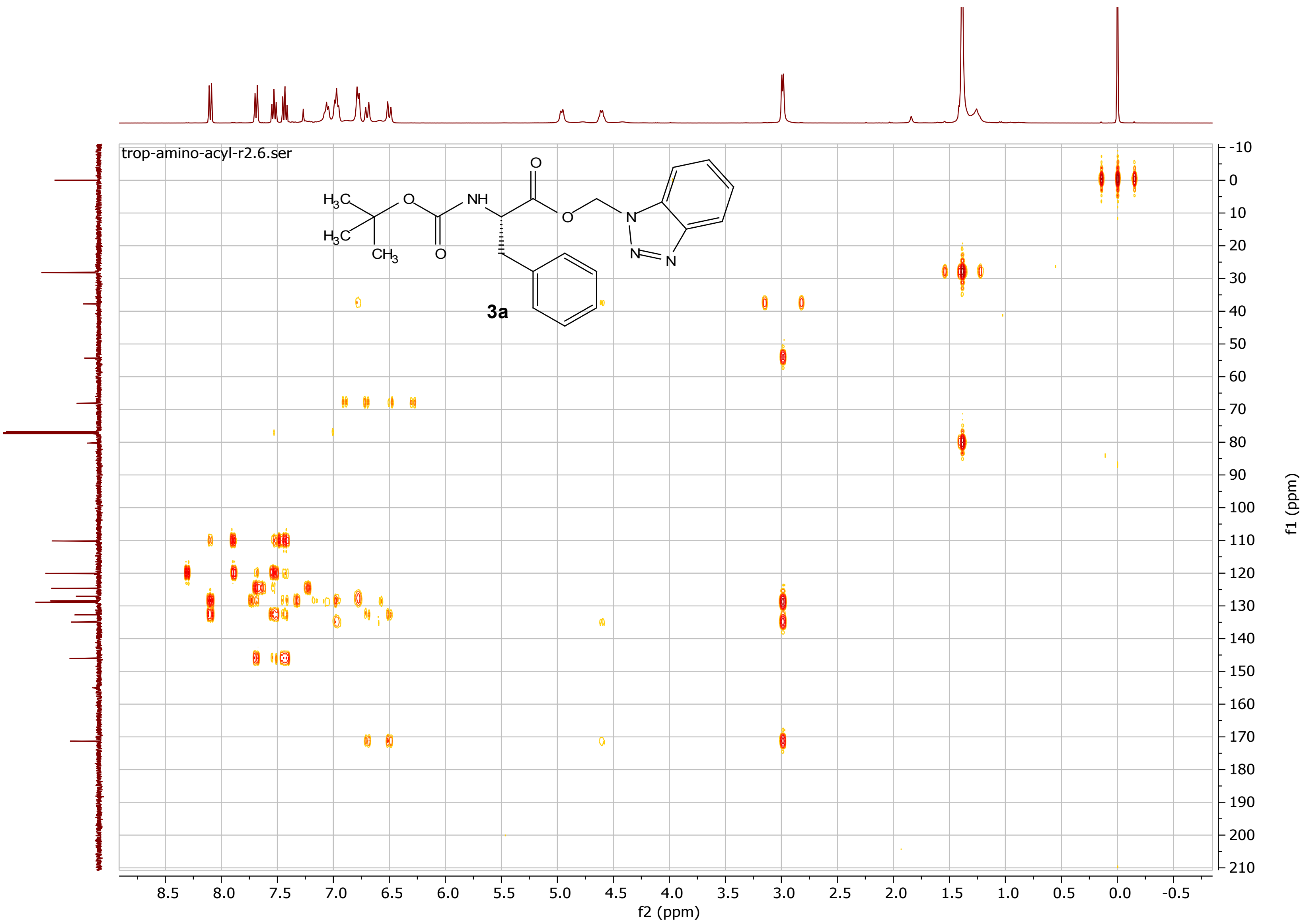

Figure S5. ¹H-¹³C HMBC of **3a**.

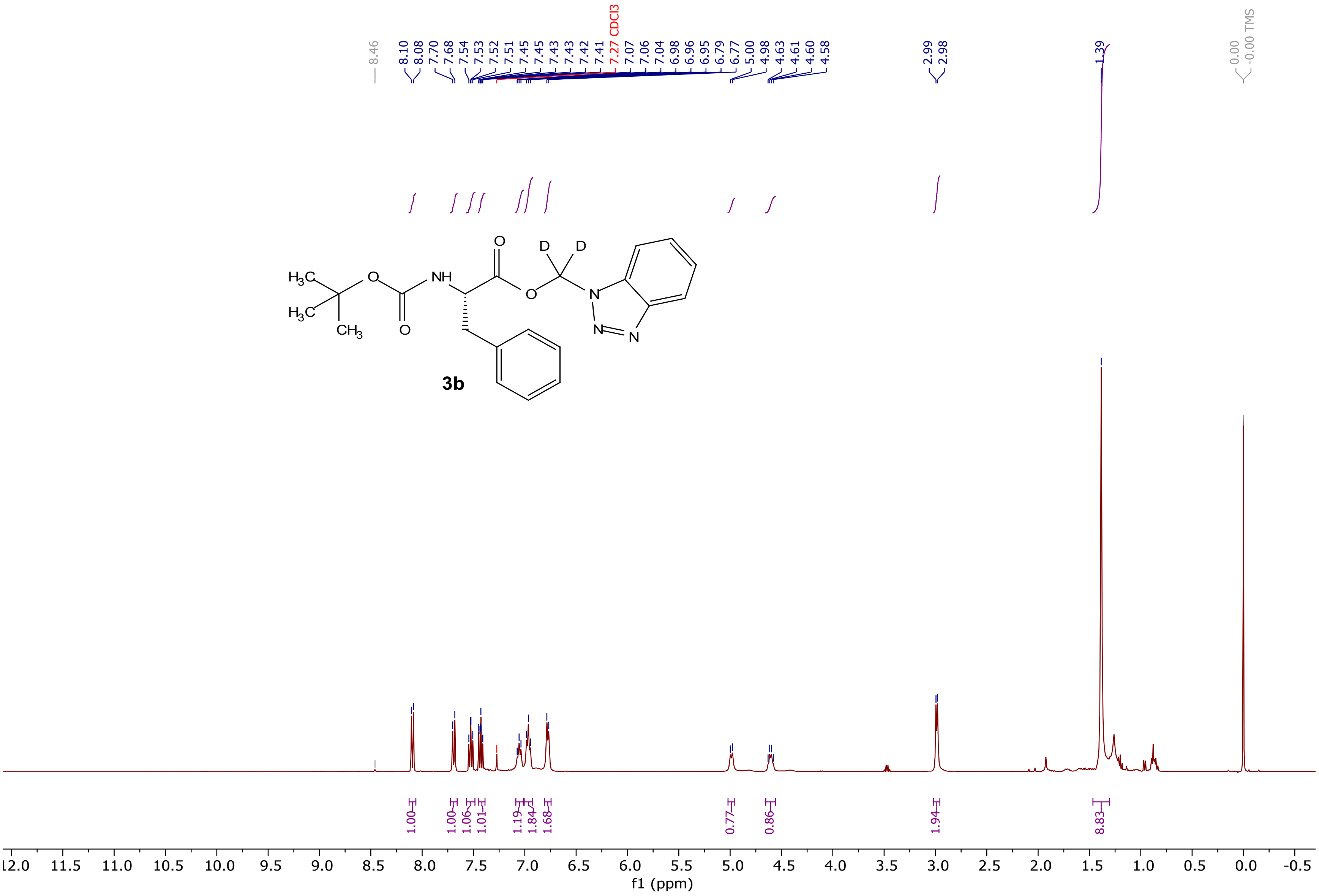

Figure S6. ^{1}H NMR of **3b**.

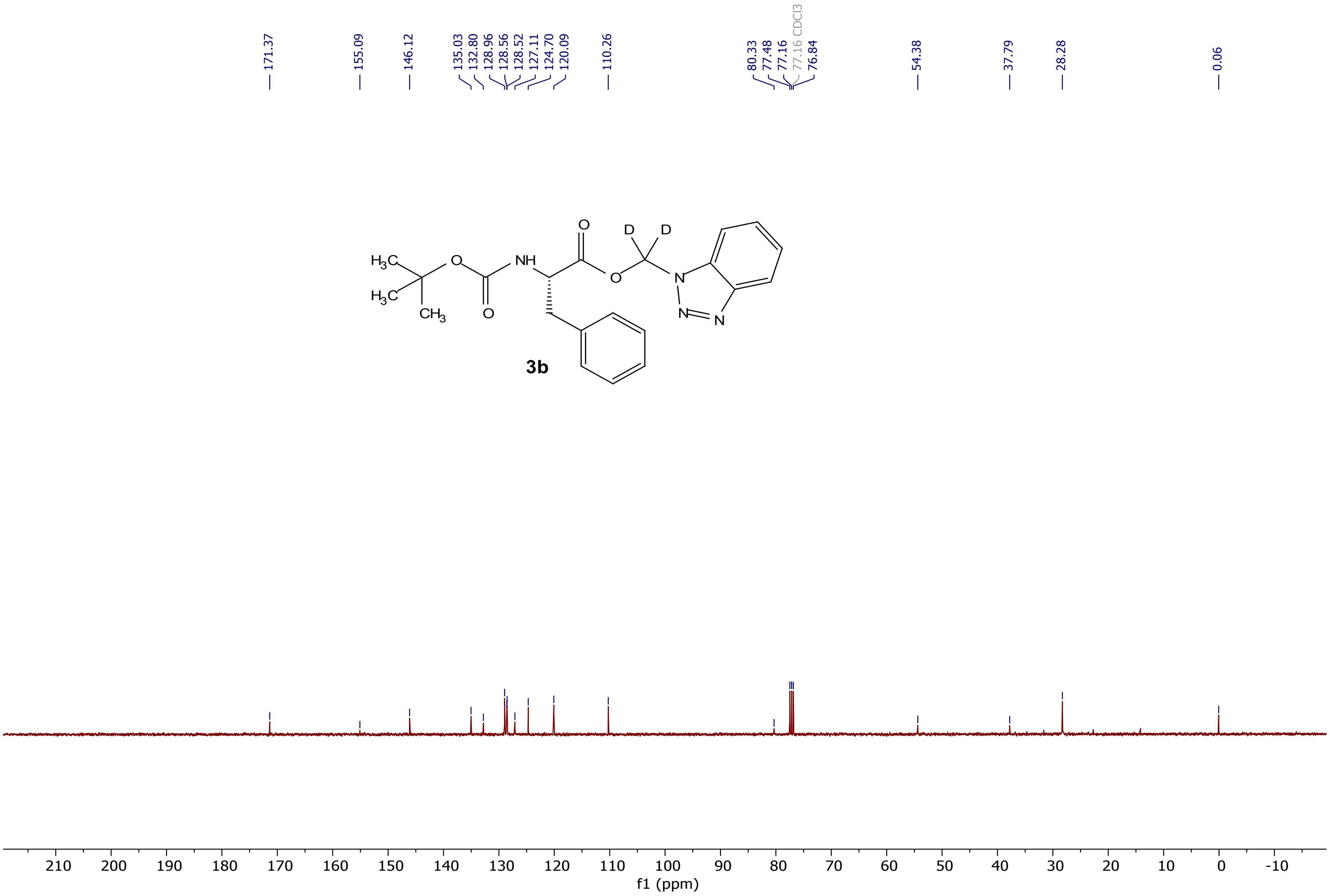

Figure S7. ^{13}C NMR of **3b**.

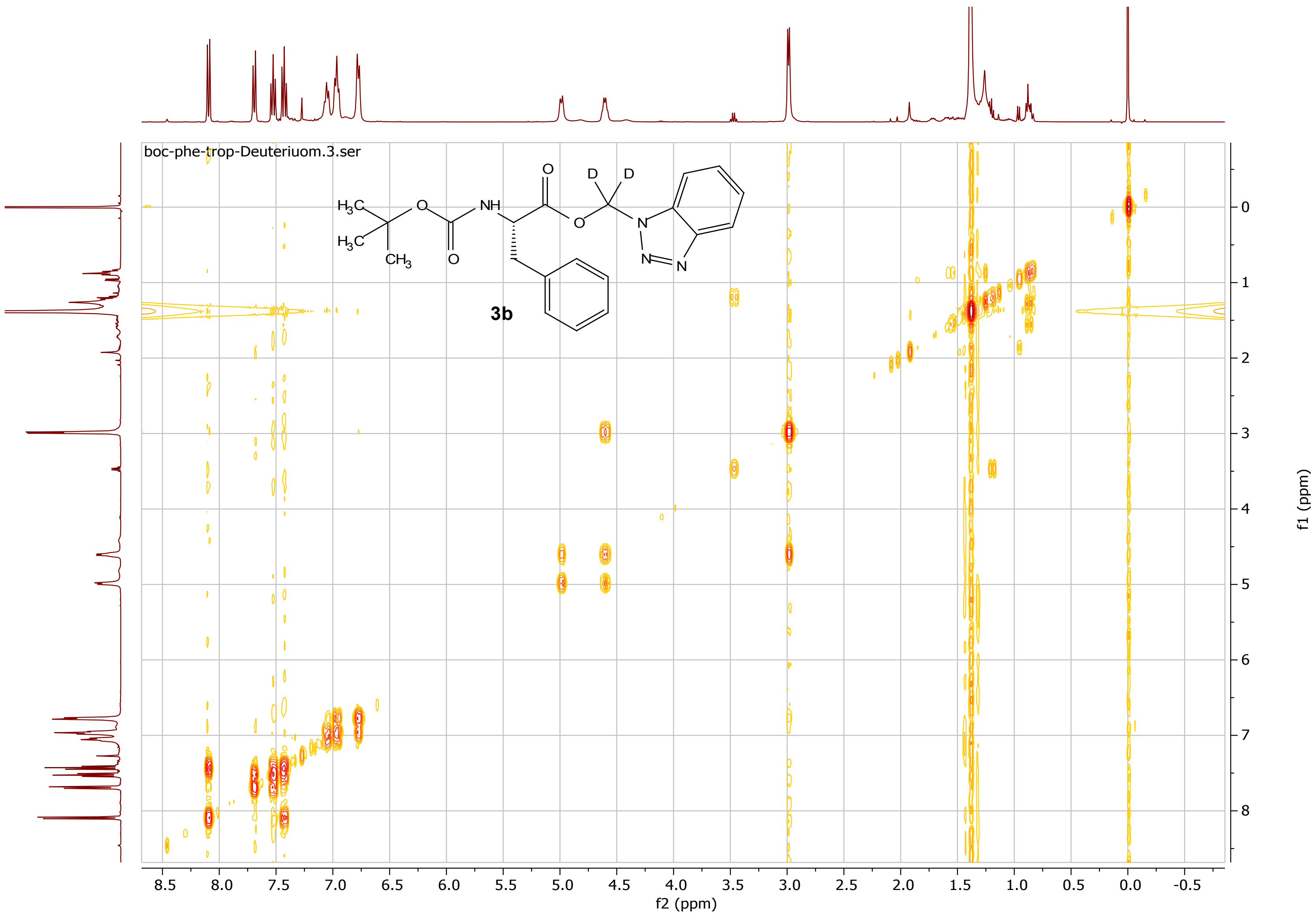

Figure S8. ^1H-^1H COSY of **3b**.

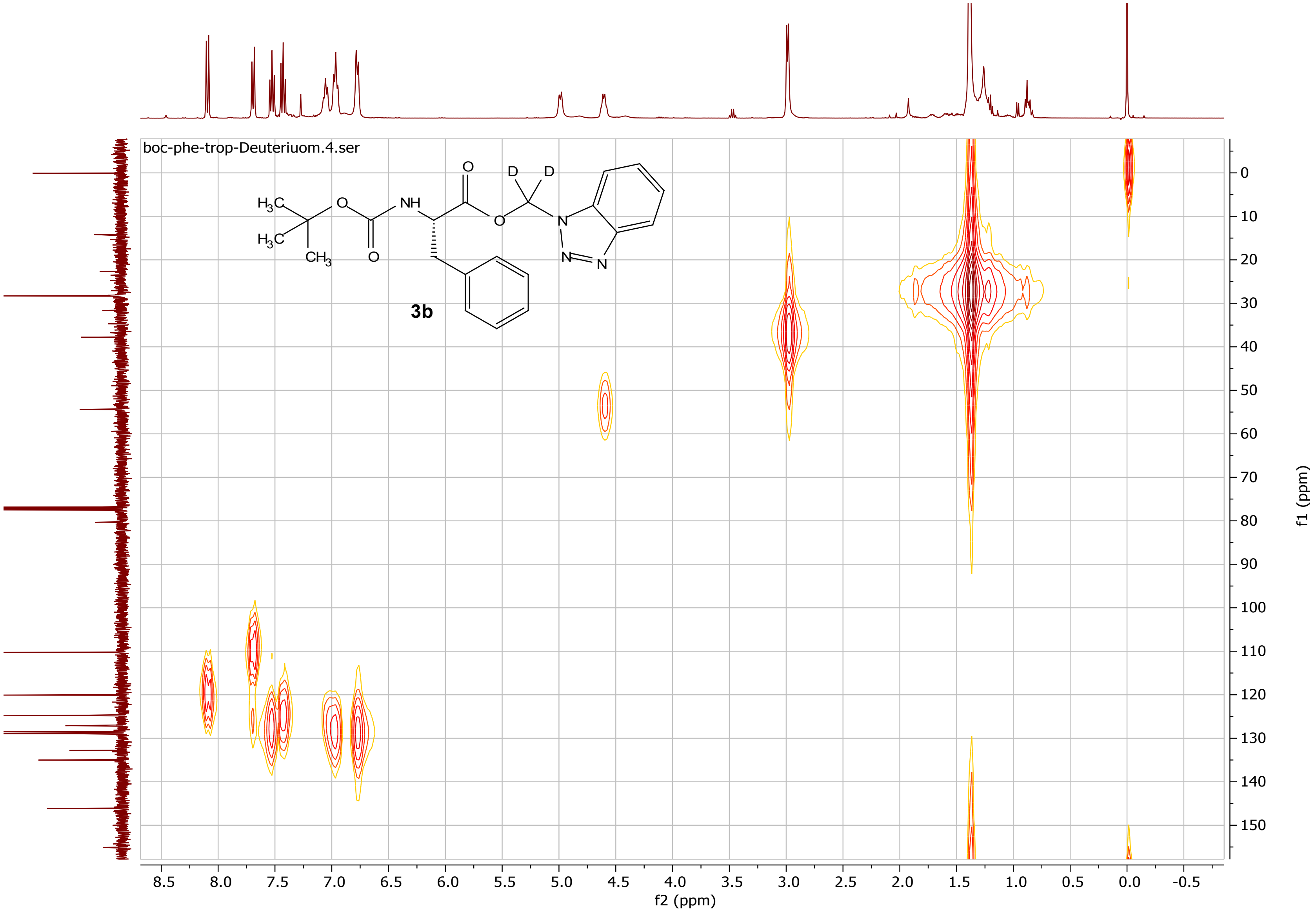

Figure S9. ^{1}H-^{13}C HMQC of **3b**.

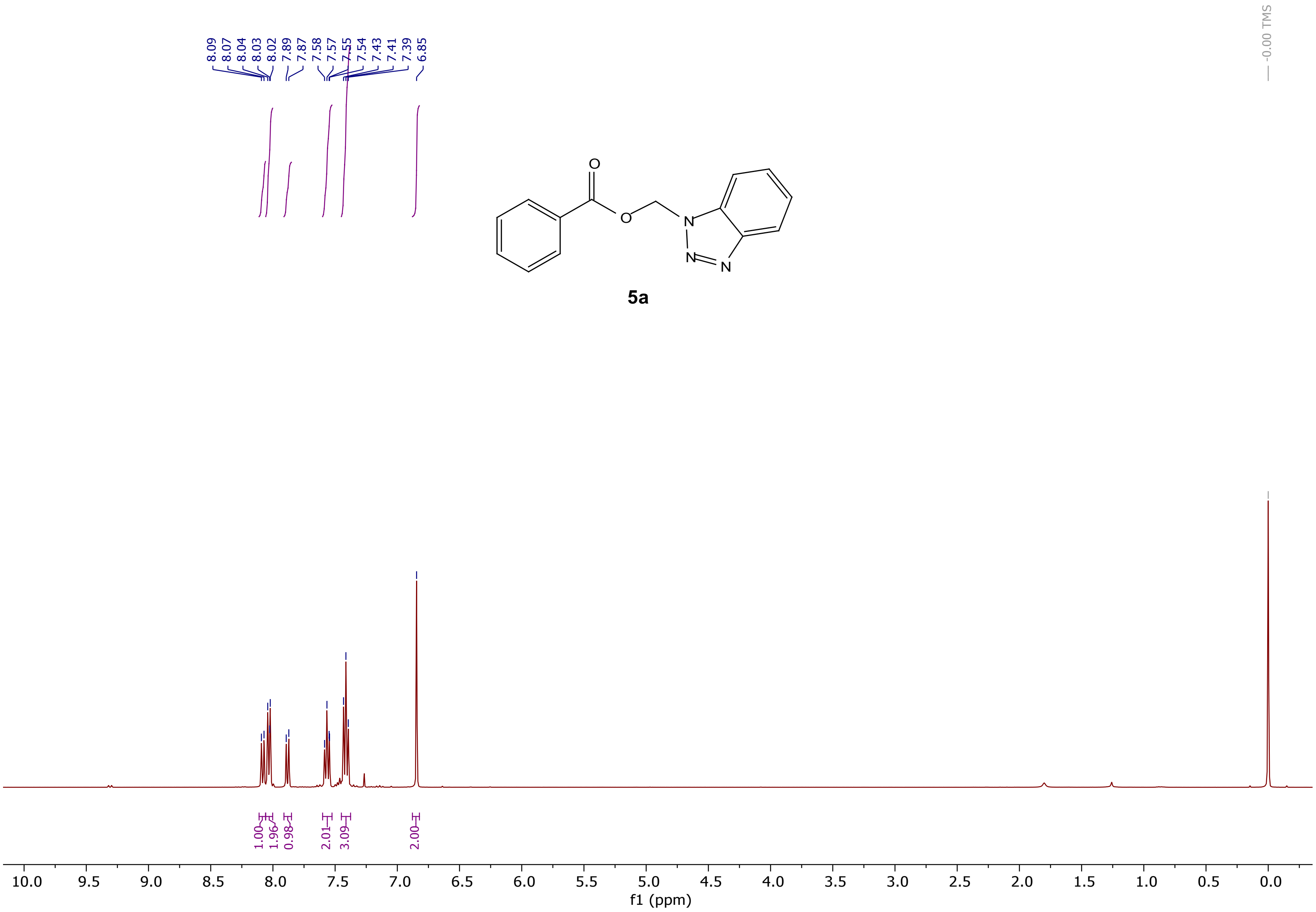

Figure S10. ^{1}H NMR of **5a**.

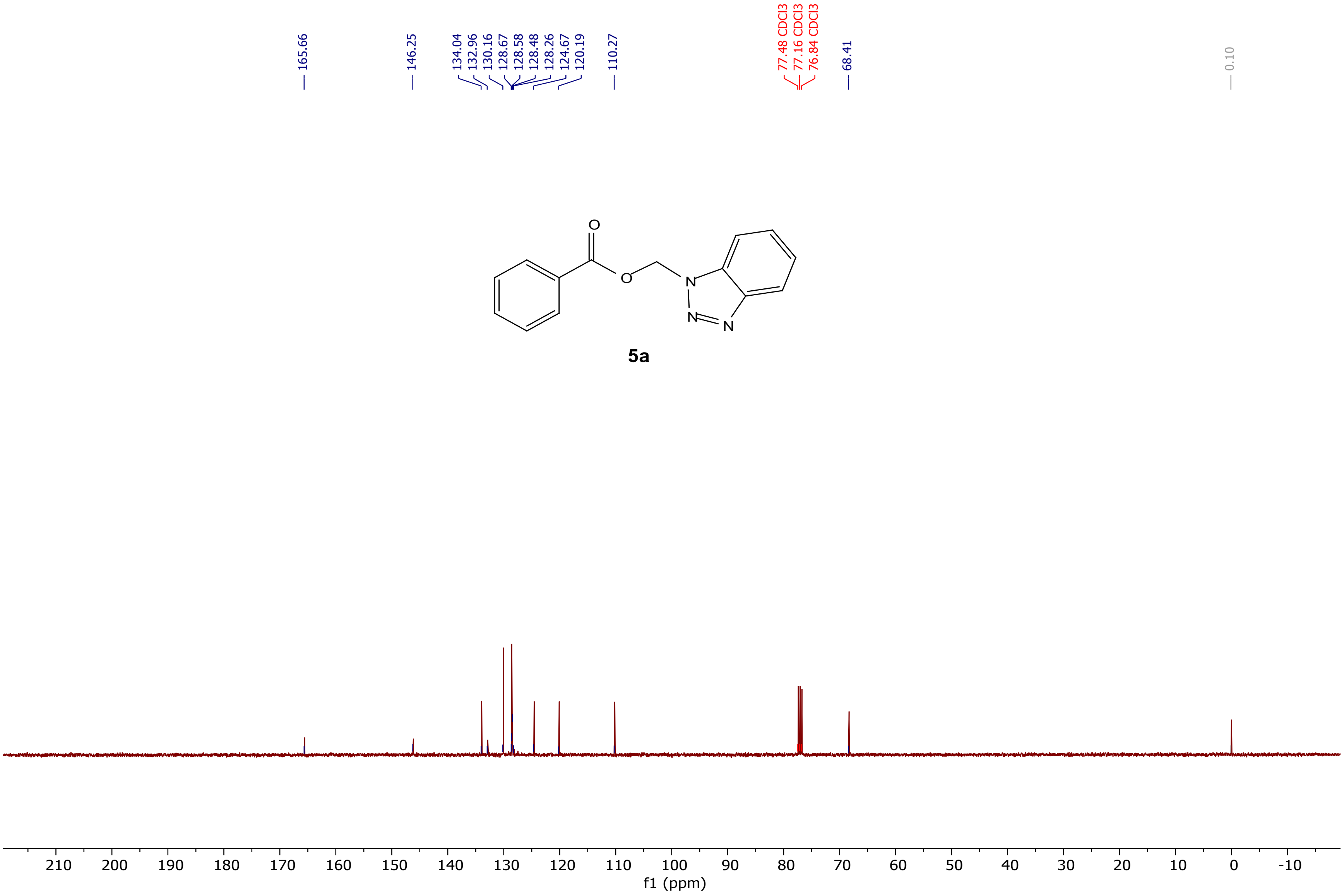

Figure S11. ^{13}C NMR of **5a**.

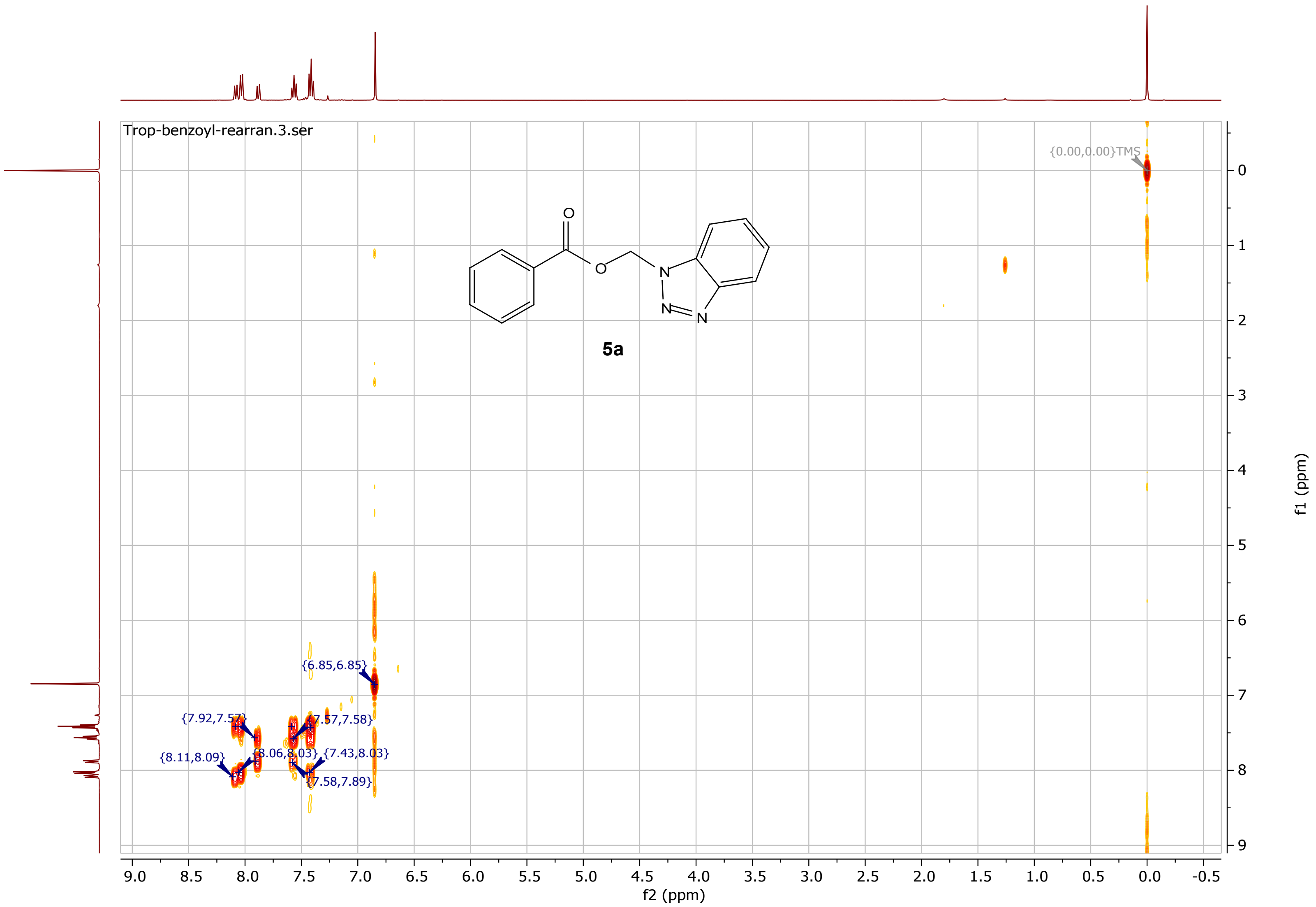

Figure S12. ^1H-^1H COSY of **5a**.

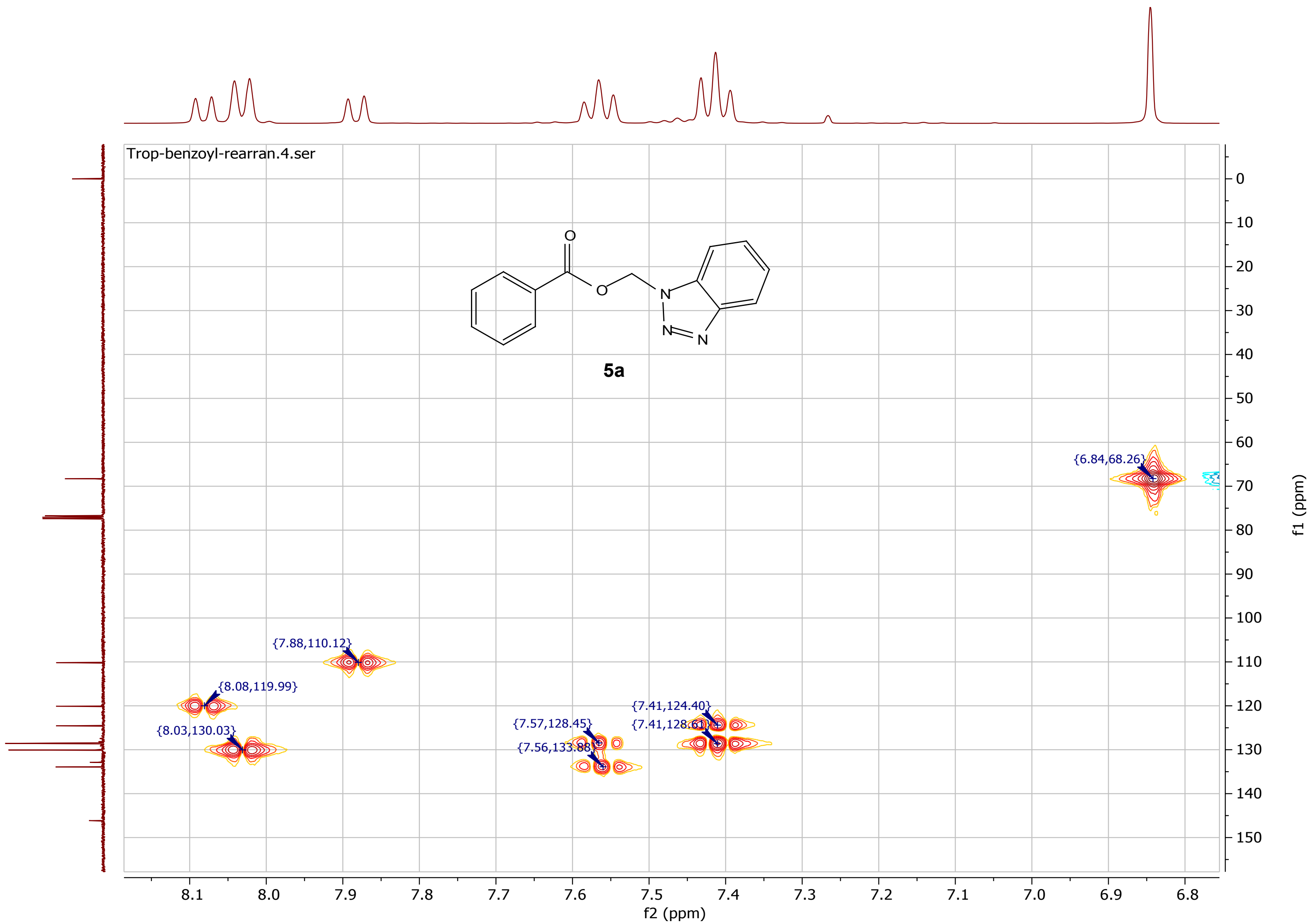

Figure S13. Expansion of ^{1}H-^{13}C HMQC of **5a**.

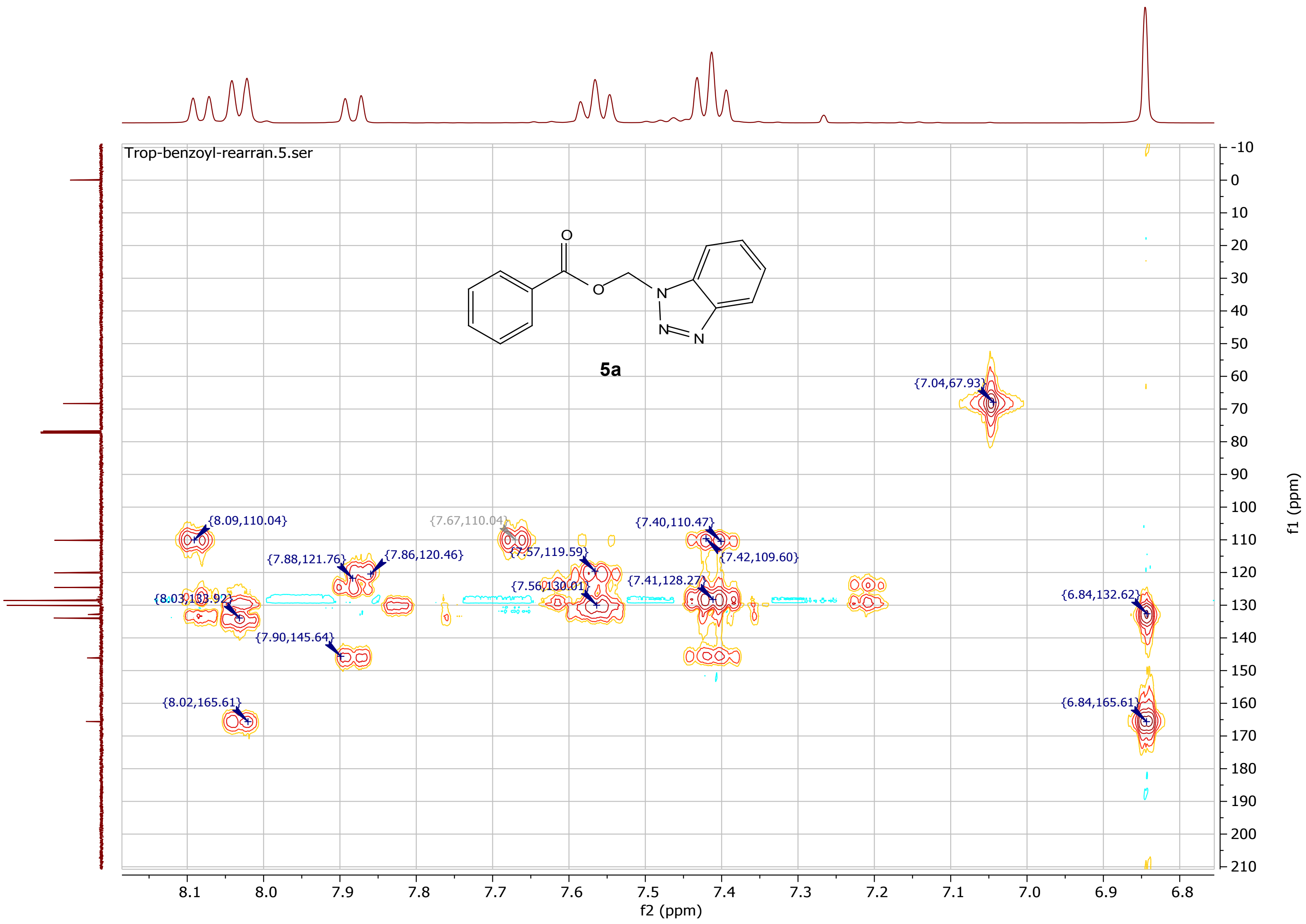

Figure S14. Expansion of ^{1}H-^{13}C HMBC of **5a**.

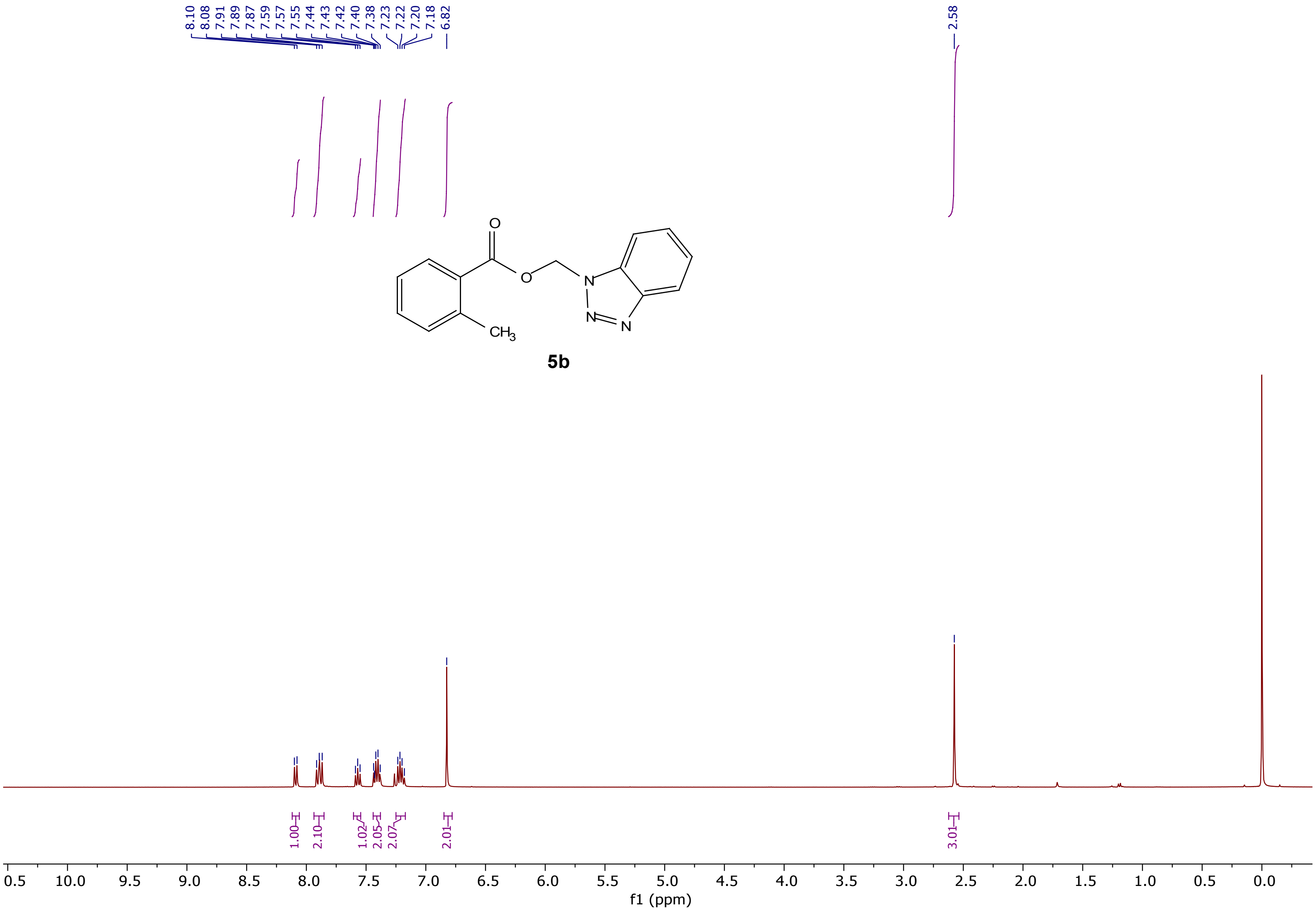

Figure S15. ^{1}H NMR of **5b**.

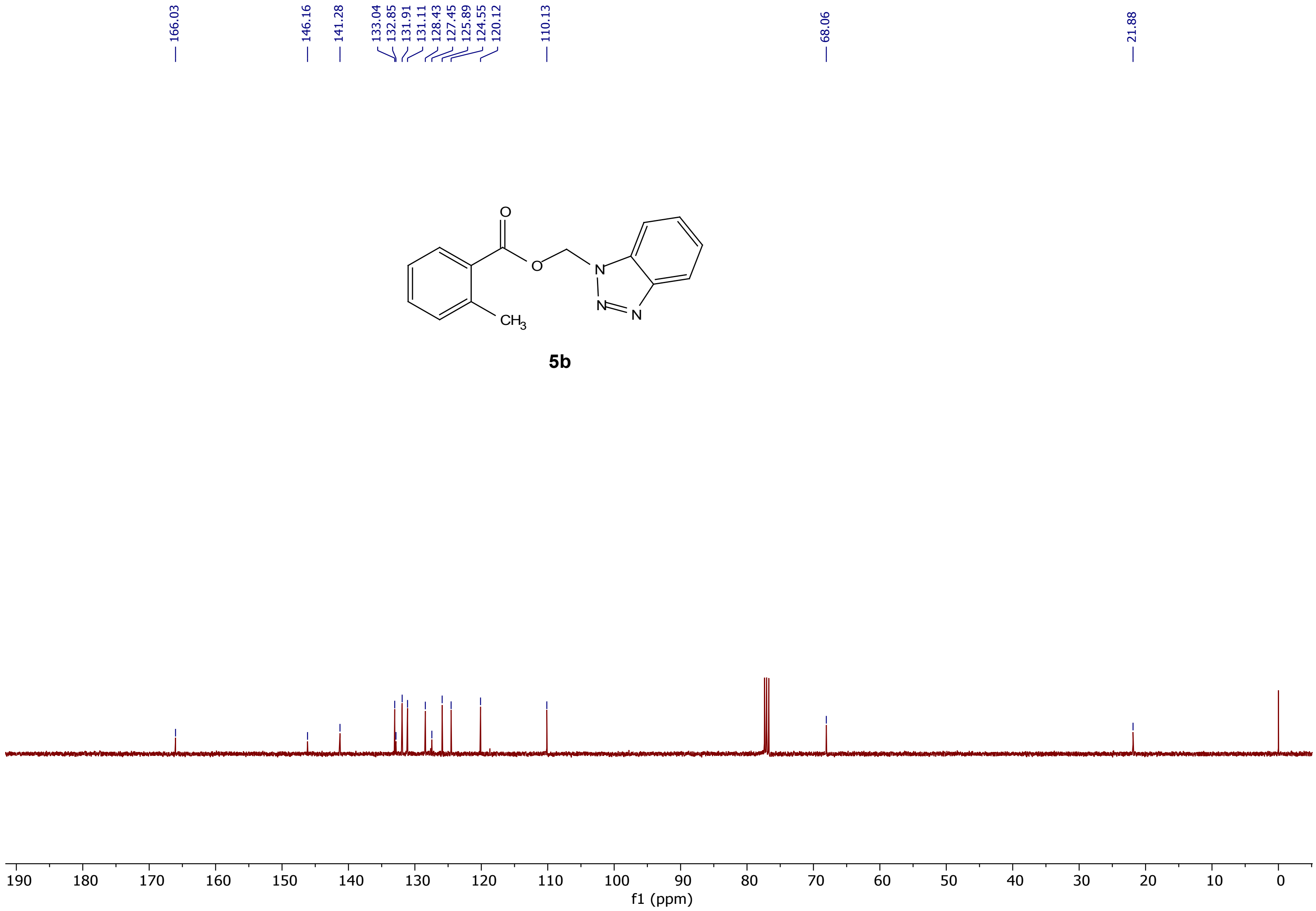

Figure S16. ^{13}C NMR of **5b**.

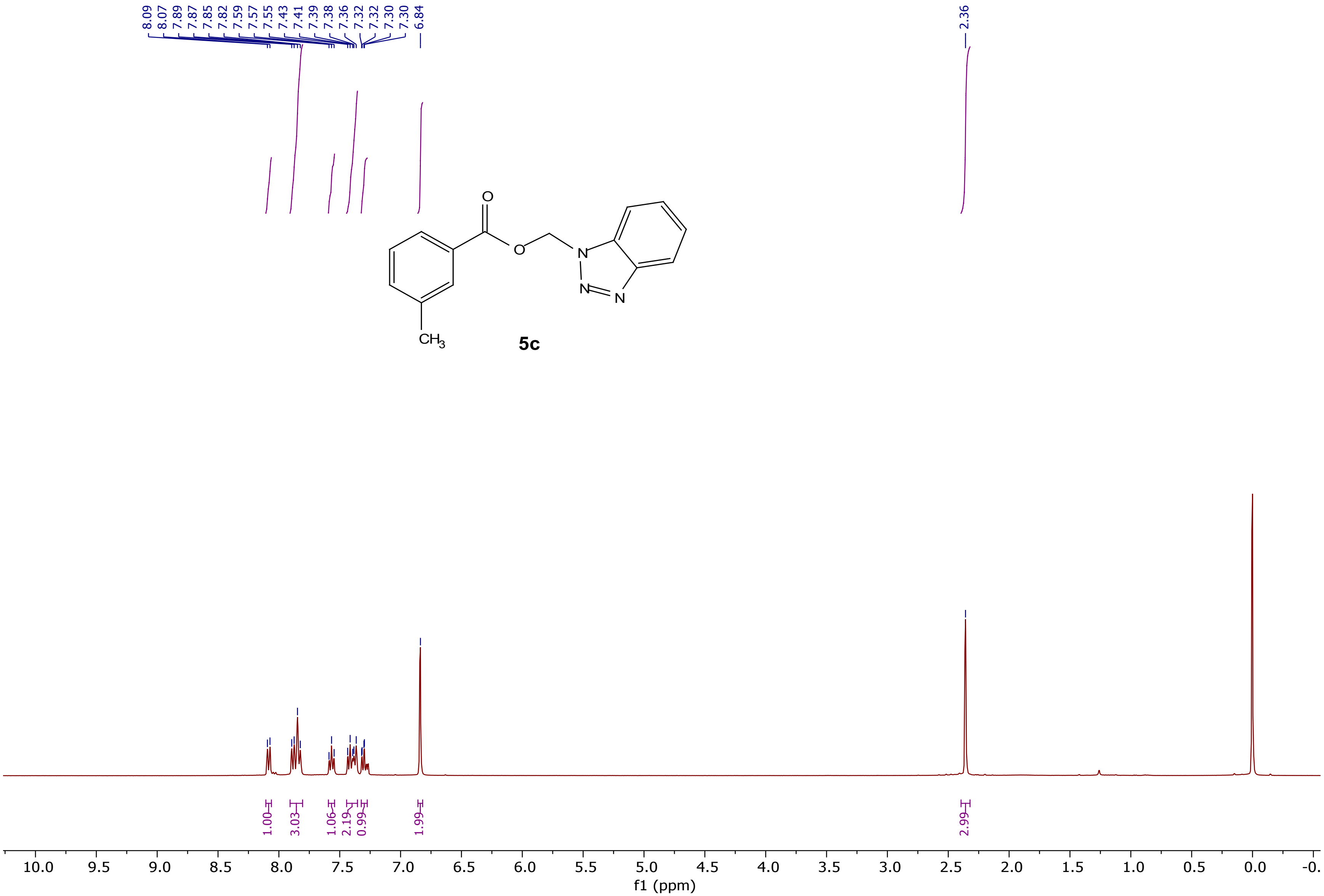

Figure S17. ^{1}H NMR of **5c**.

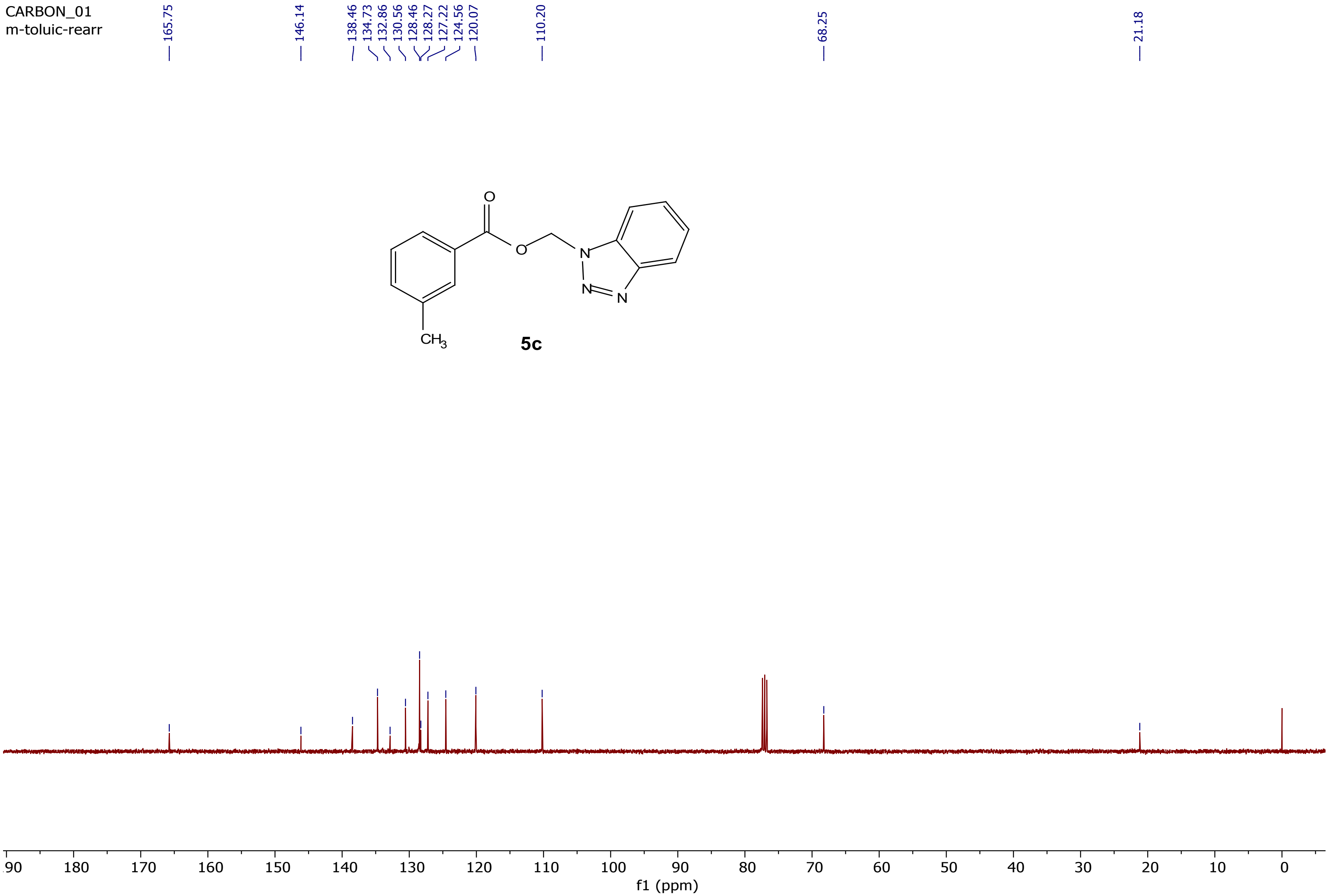

Figure S18. ^{13}C NMR of **5c**.

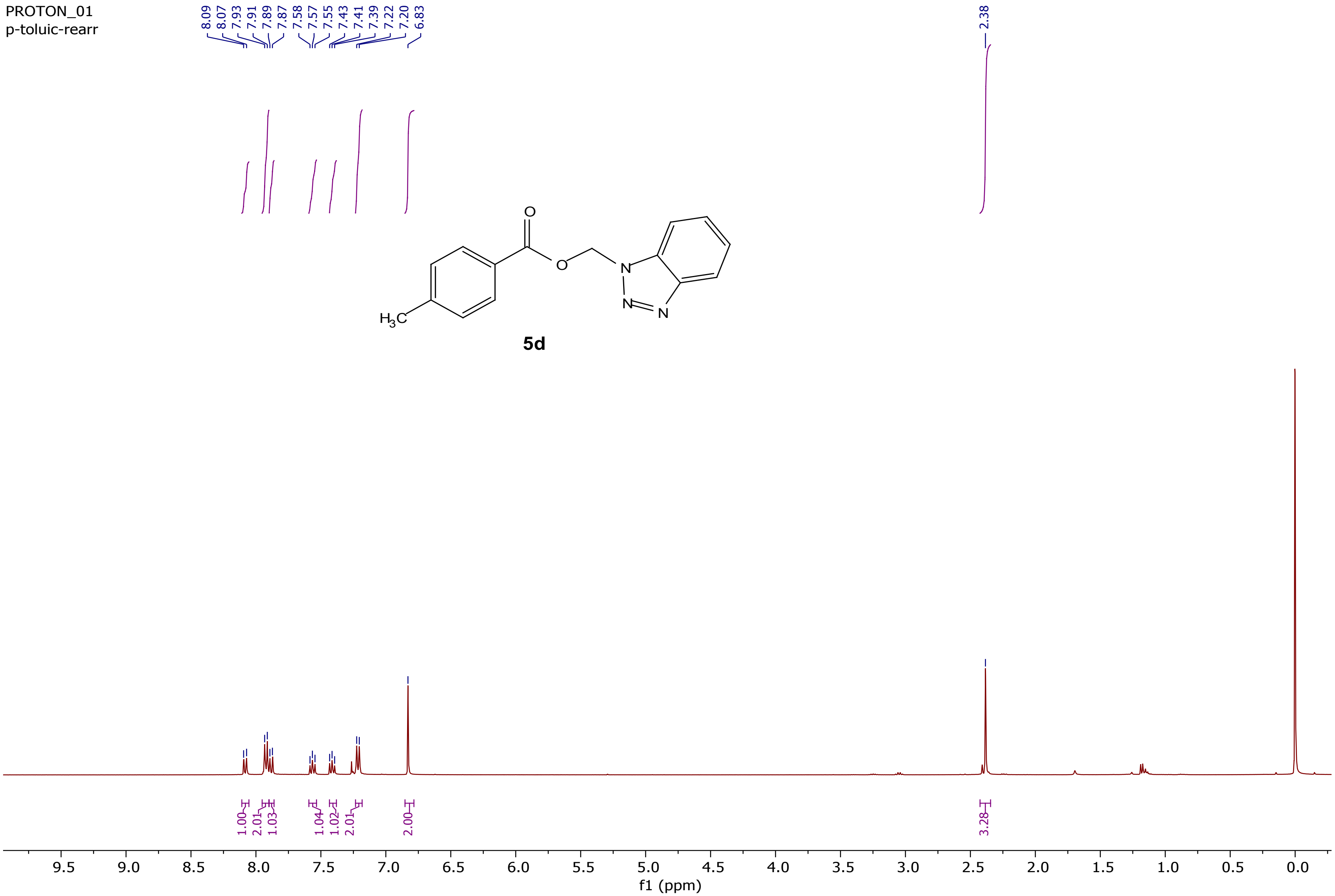

Figure S19. ^{1}H NMR of **5d**.

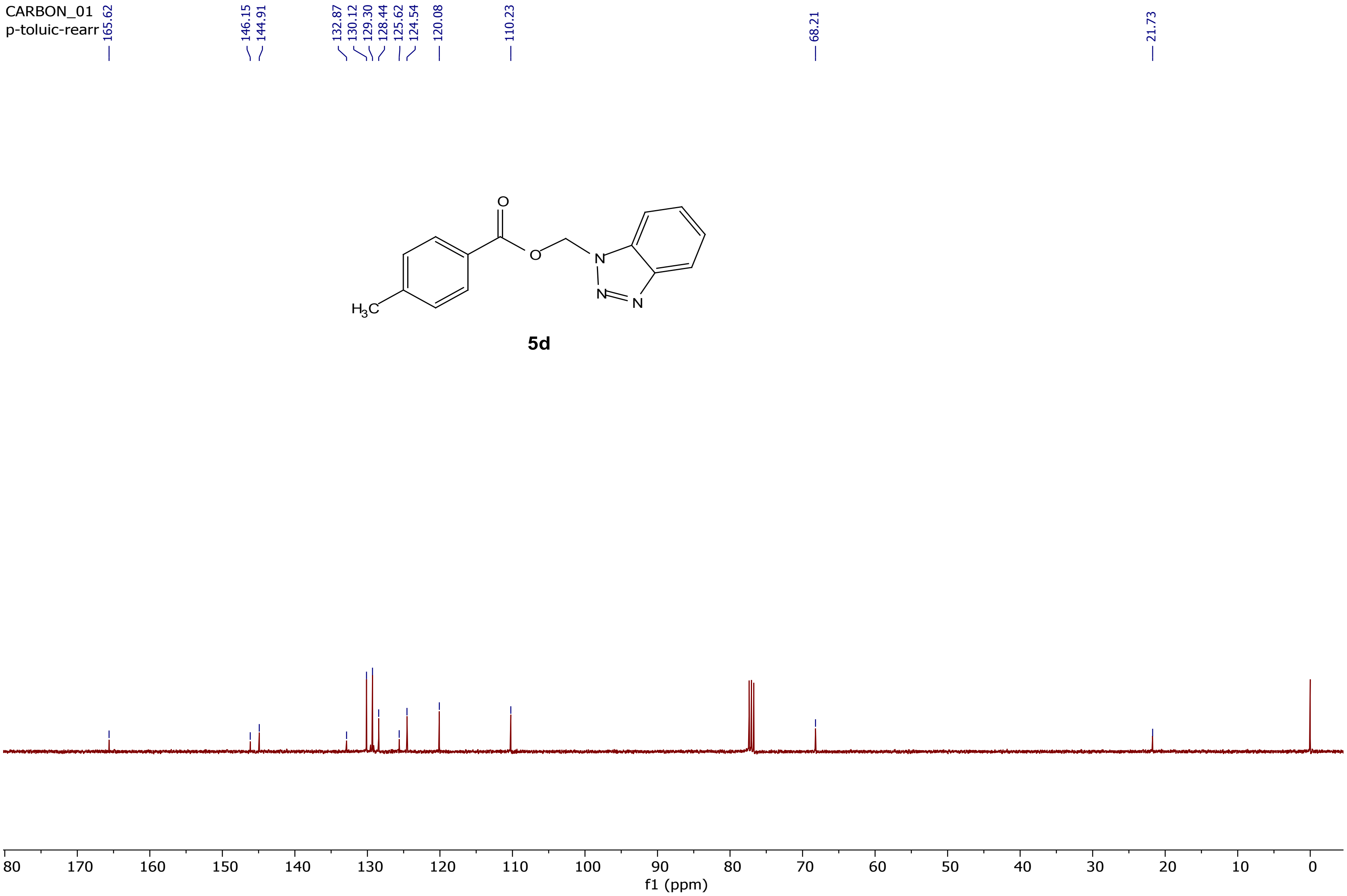

Figure S20. ^{13}C NMR of **5d**.

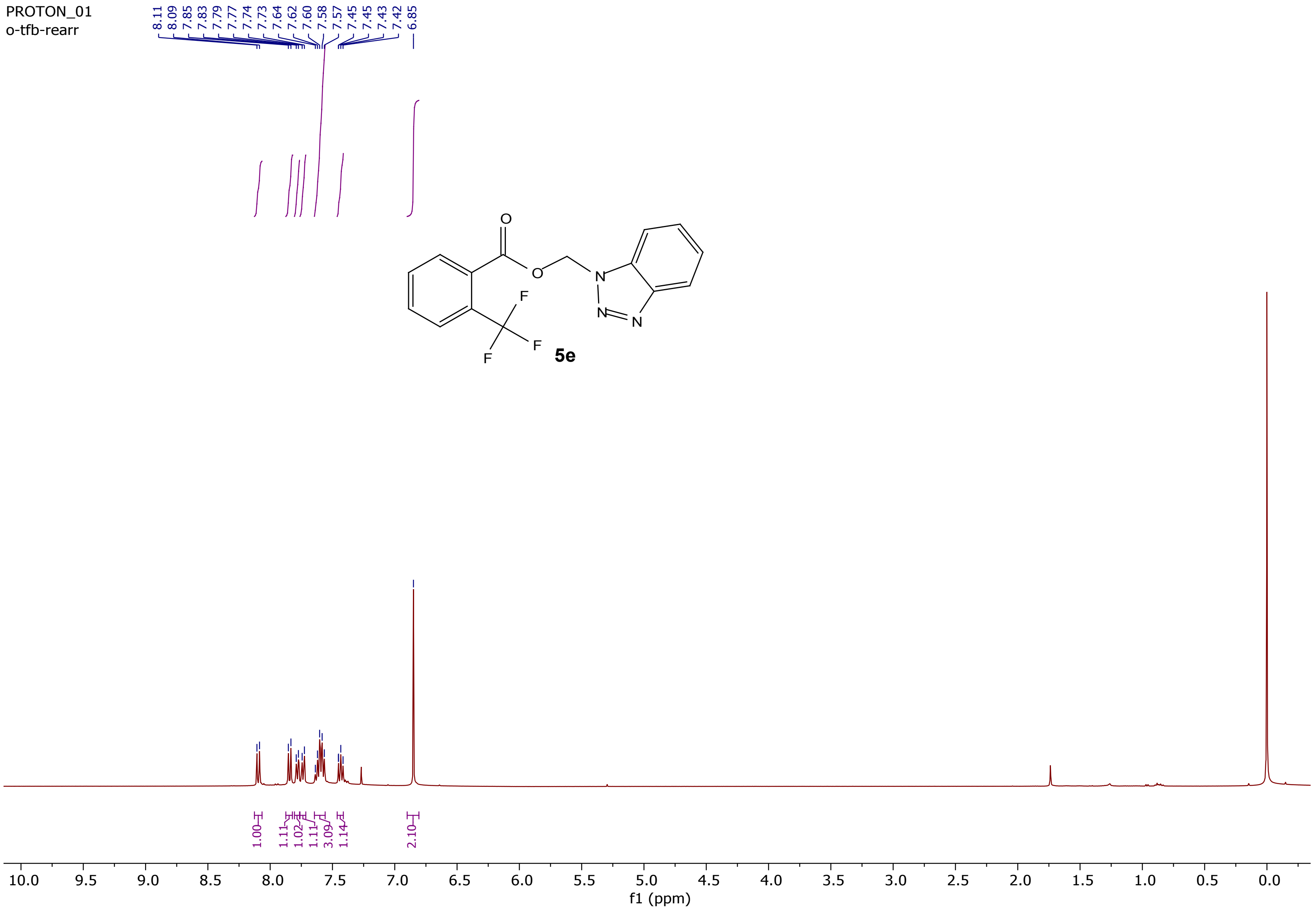

Figure S21. ^{1}H NMR of **5e**.

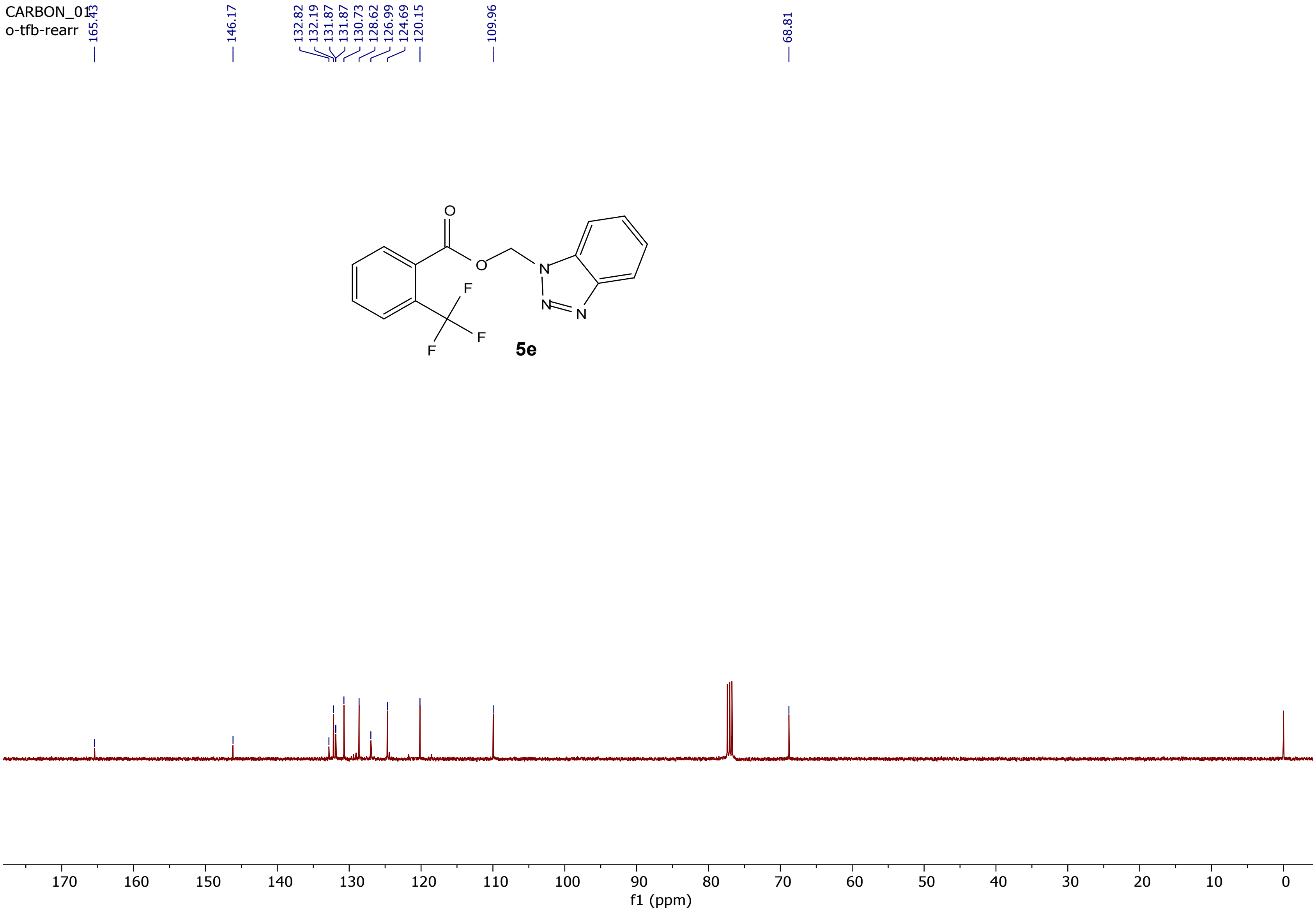

Figure S22. ^{13}C NMR of **5e**.

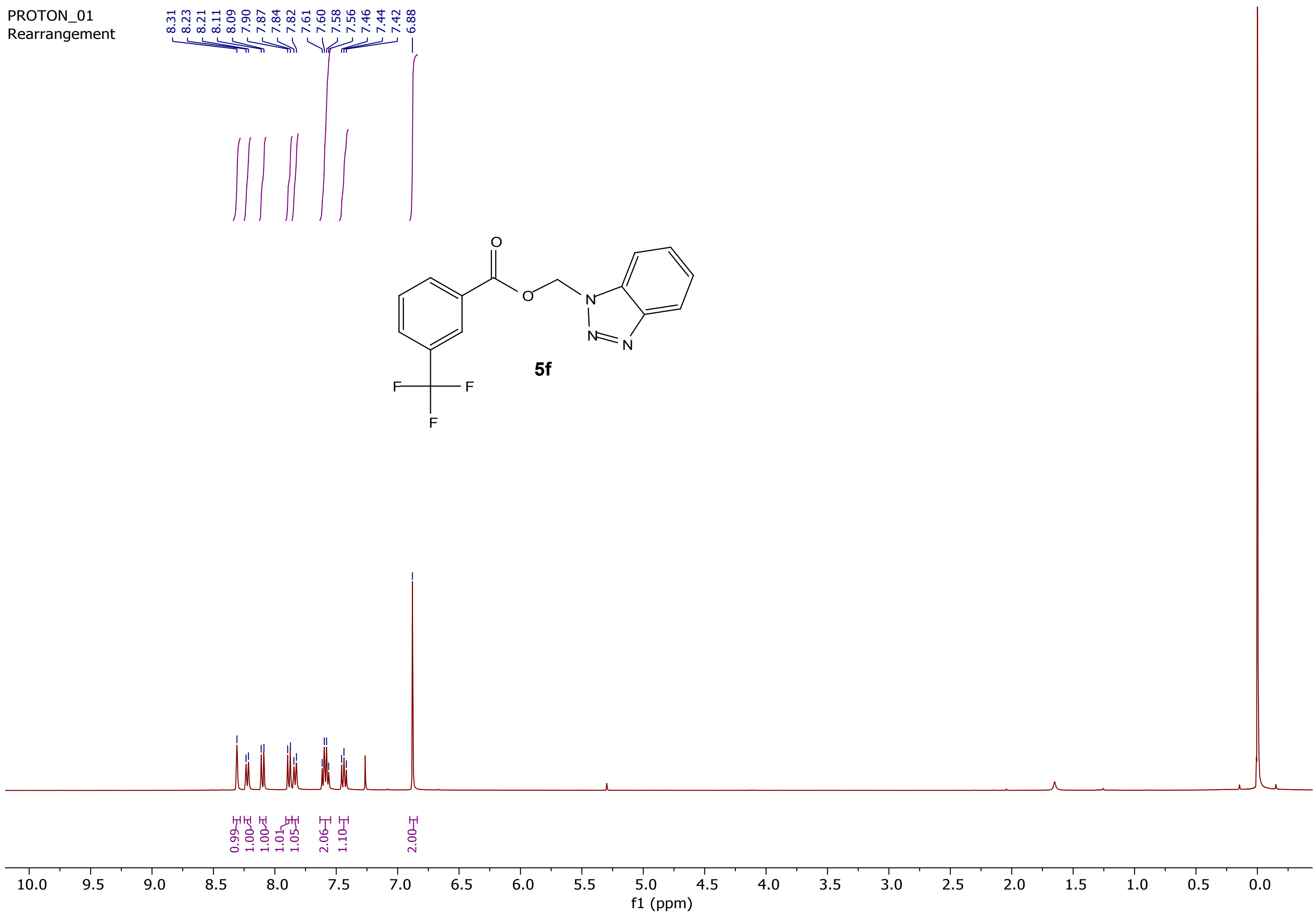

Figure S23. ^{1}H NMR of **5f**.

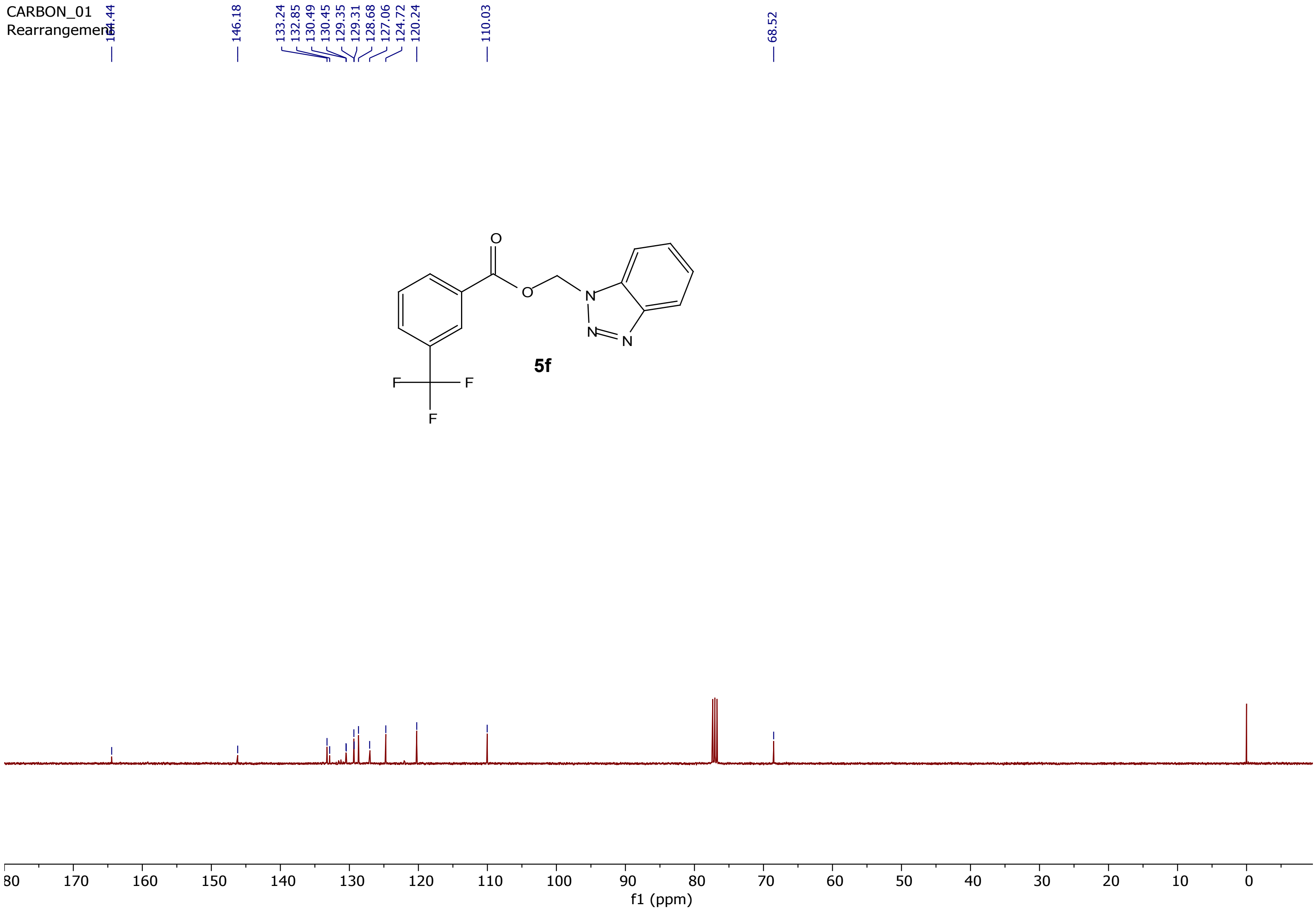

Figure S24. ^{13}C NMR of **5f**.

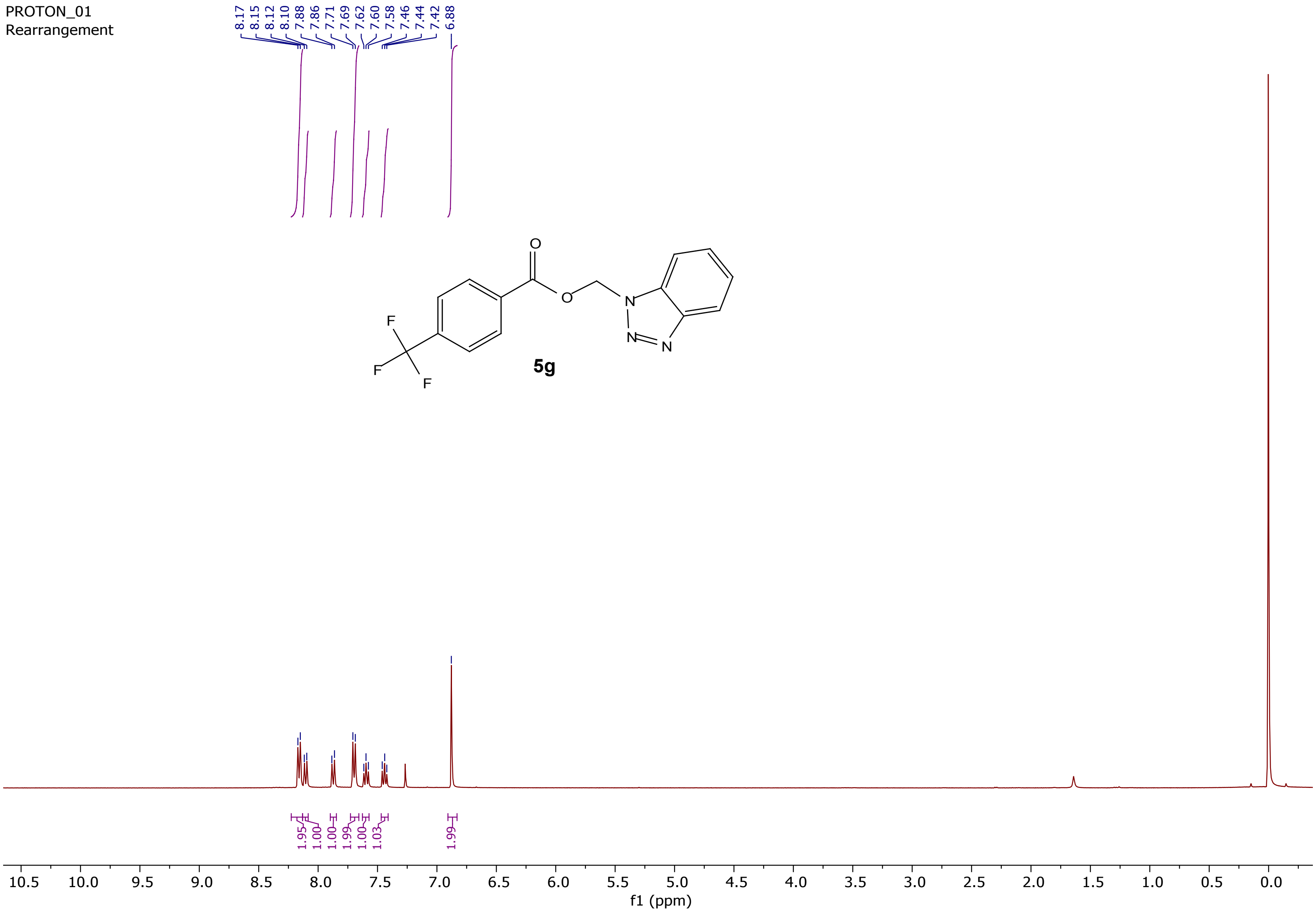

Figure S25. ^{1}H NMR of **5g**.

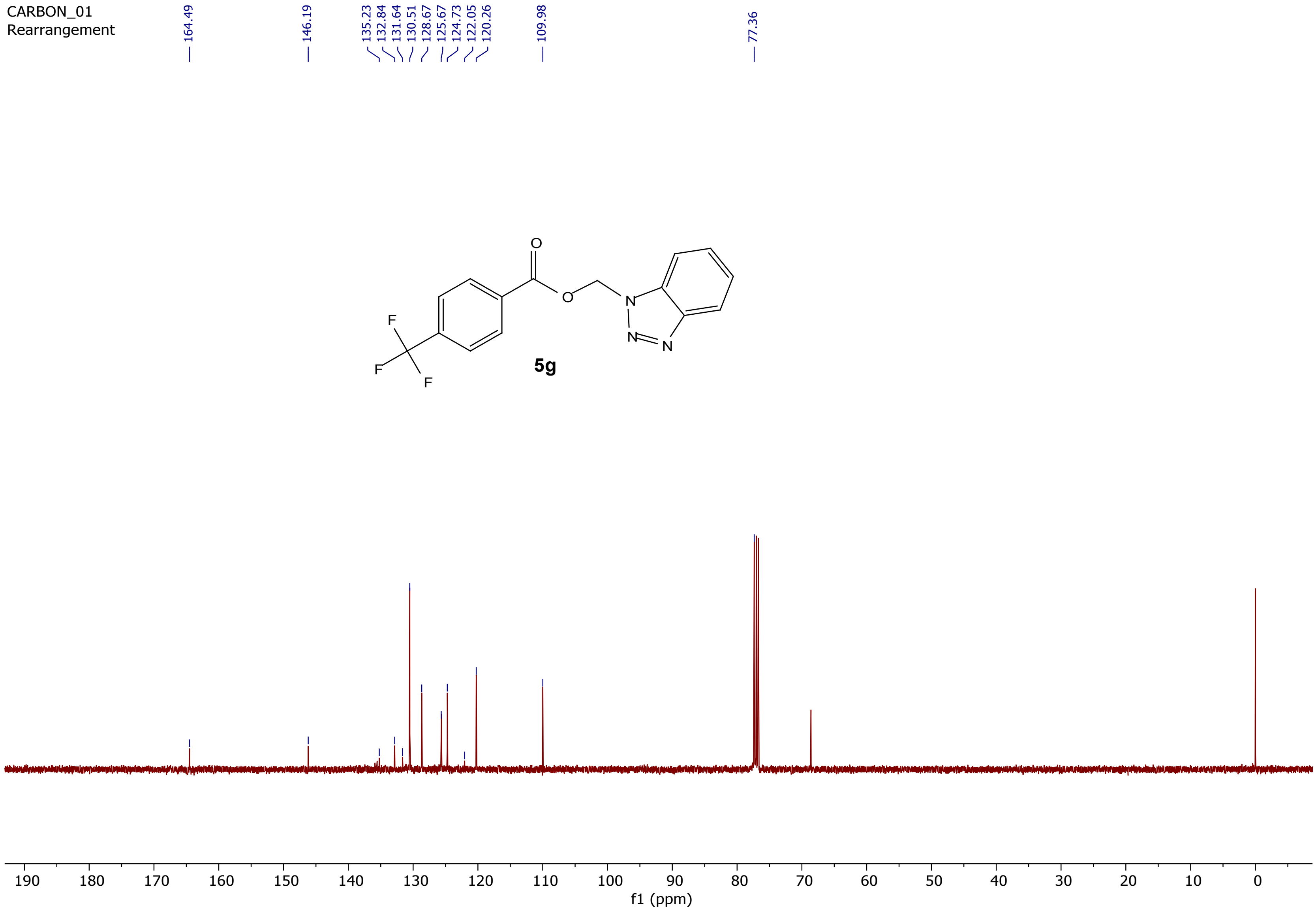

Figure S26. ^{13}C NMR of **5g**.

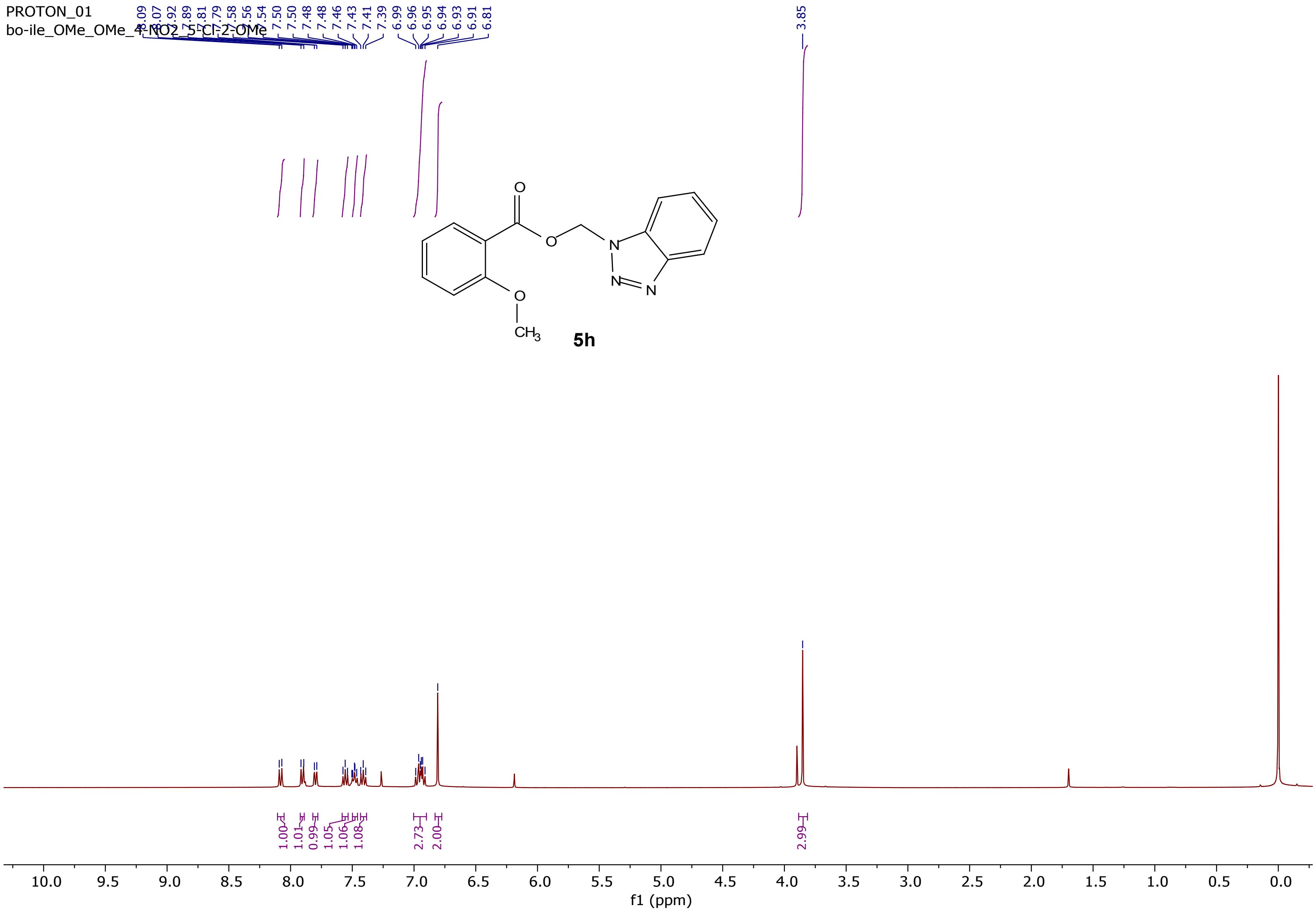

Figure S27. ^{1}H NMR of **5h**.

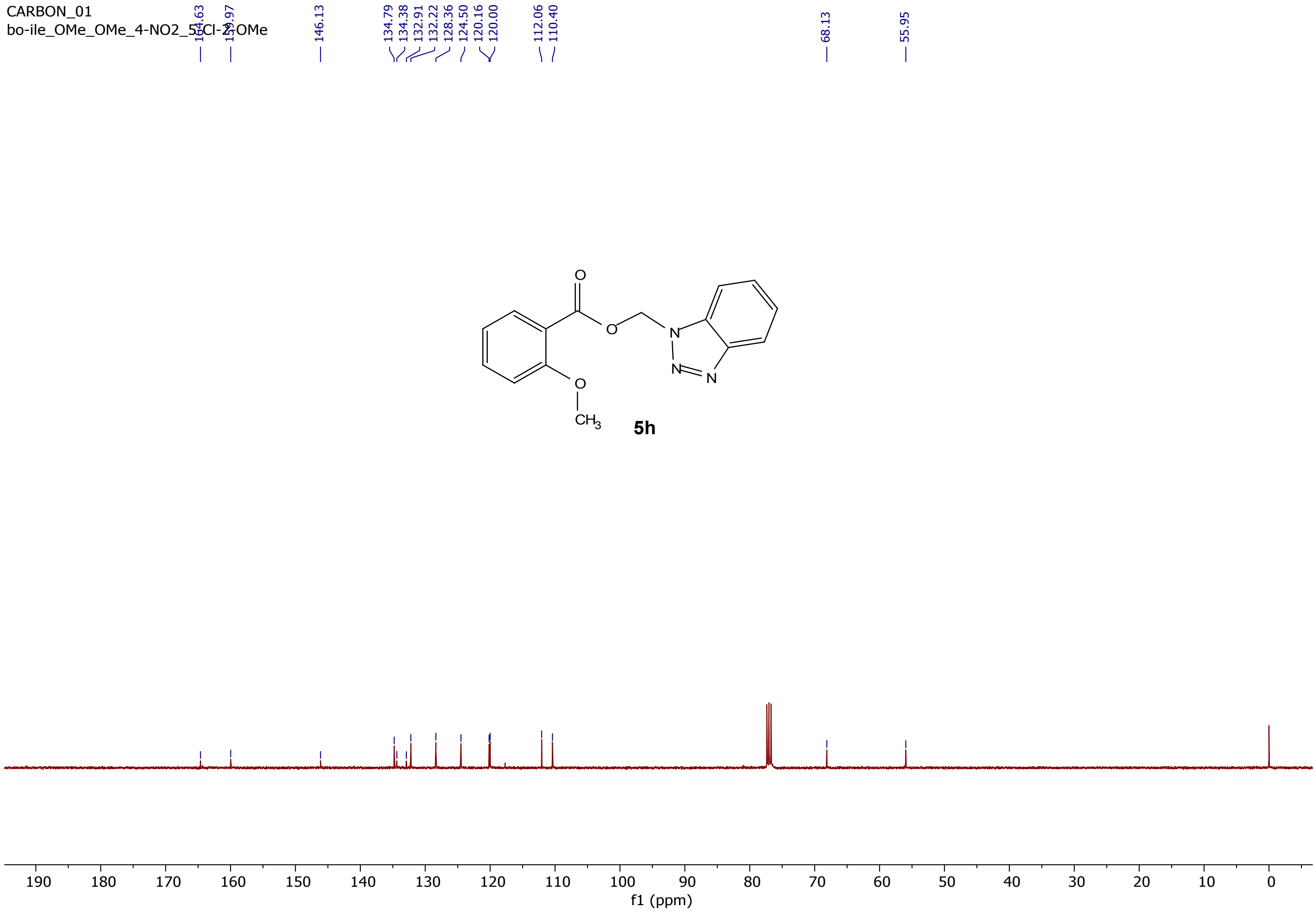

Figure S28. ^{13}C NMR of **5h**.

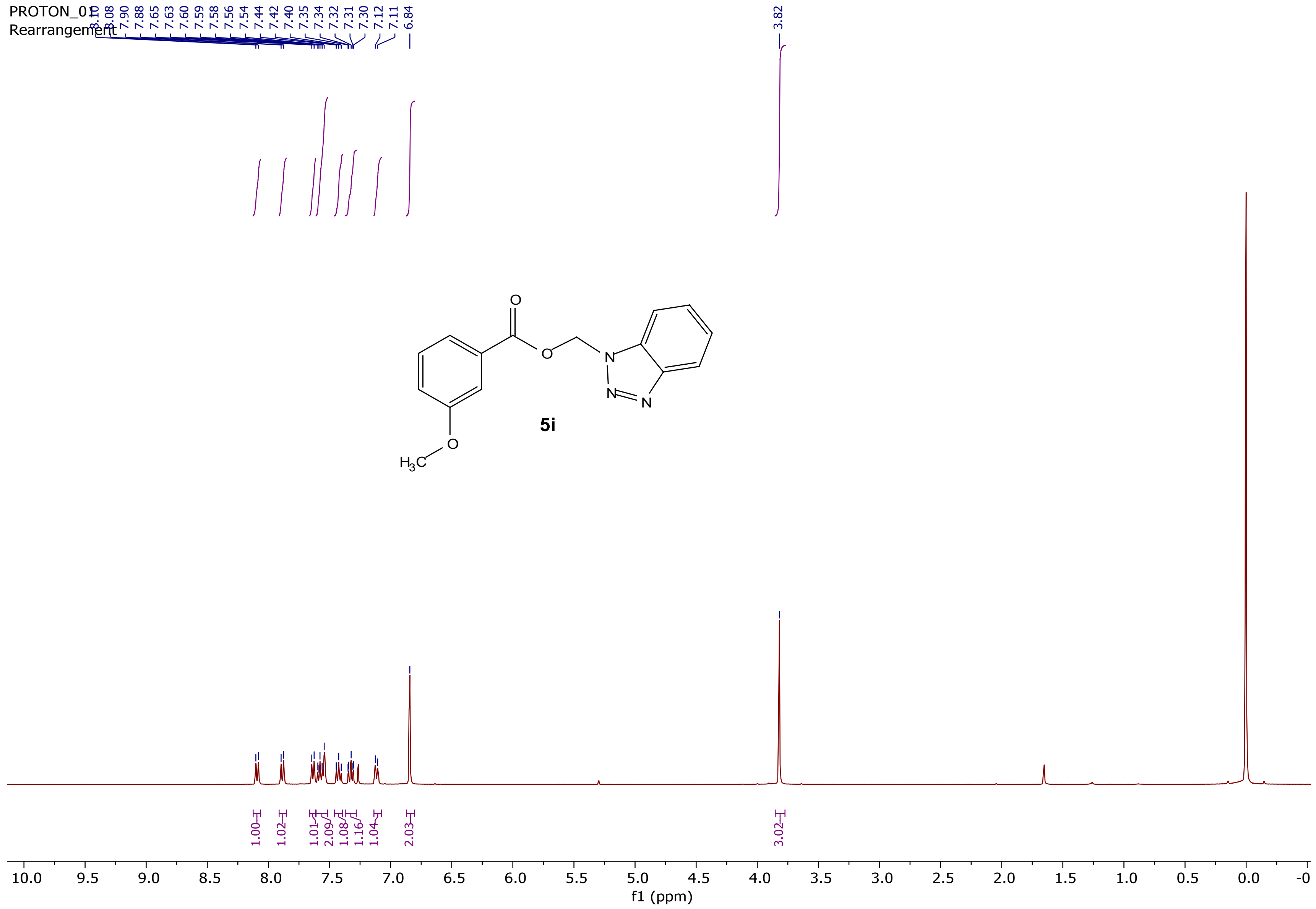

Figure S29. ^{1}H NMR of **5i**.

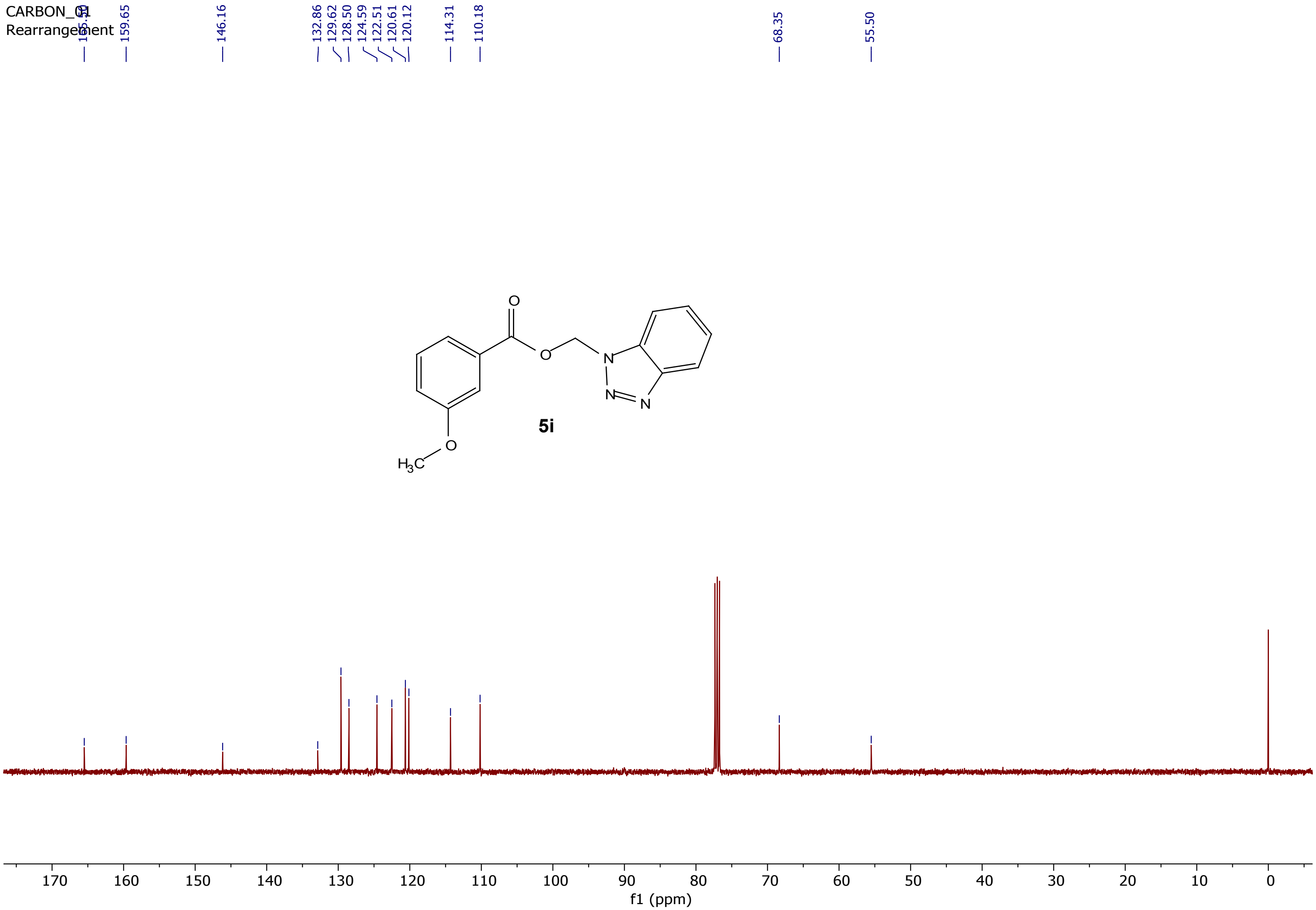

Figure S30. ^{13}C NMR of **5i**.

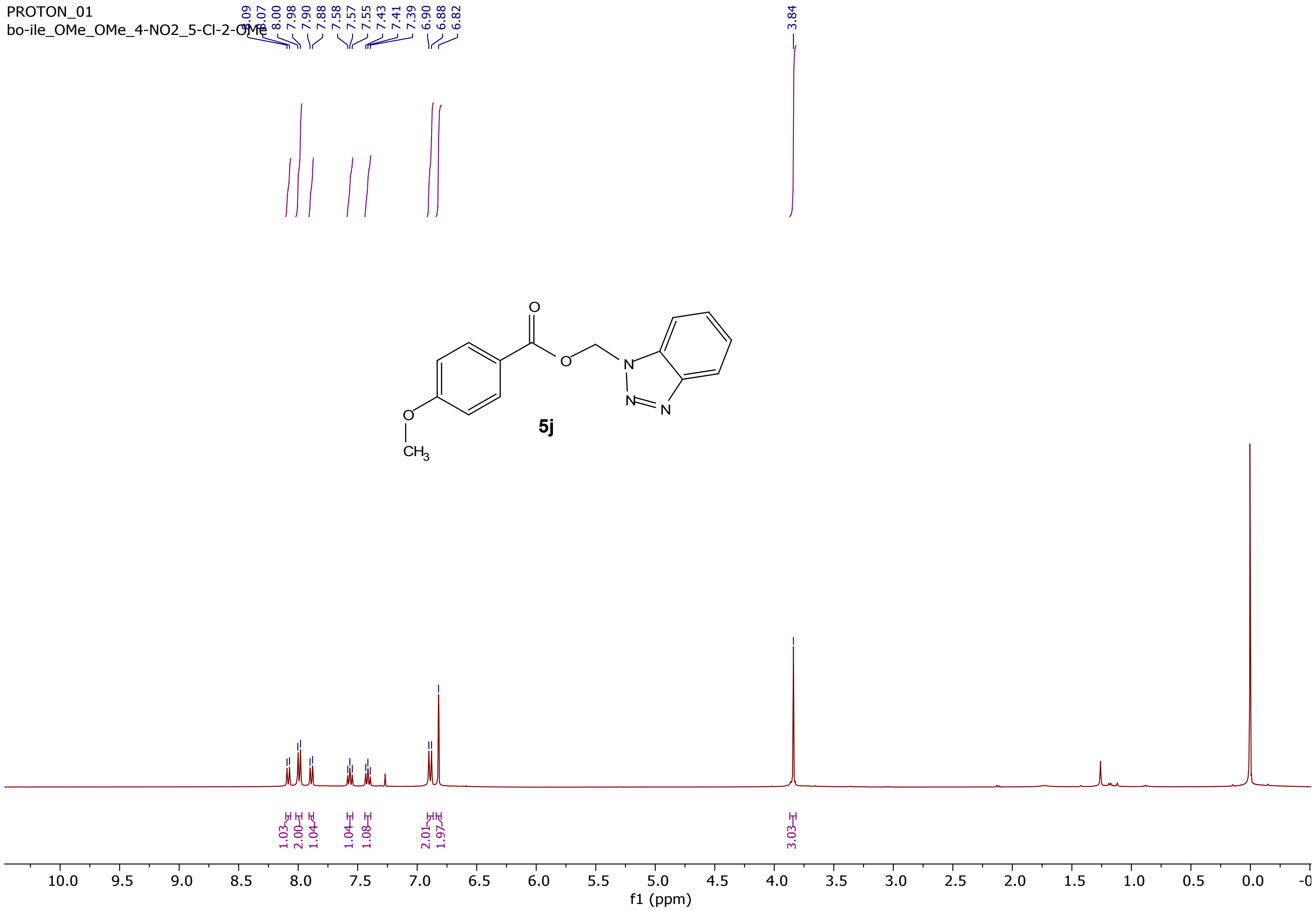

Figure S31. ^{1}H NMR of **5j**.

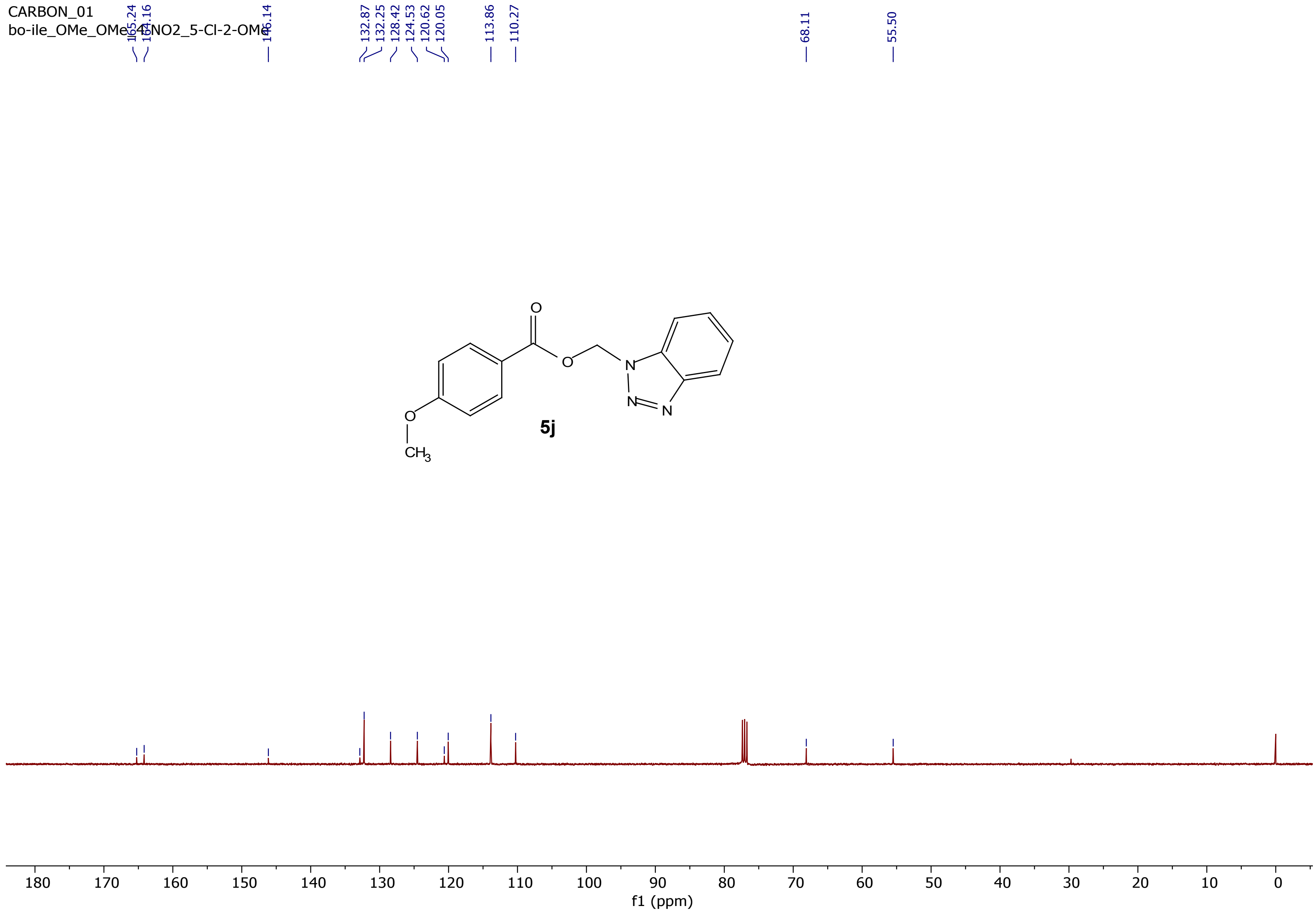

Figure S32. ^{13}C NMR of **5j**.

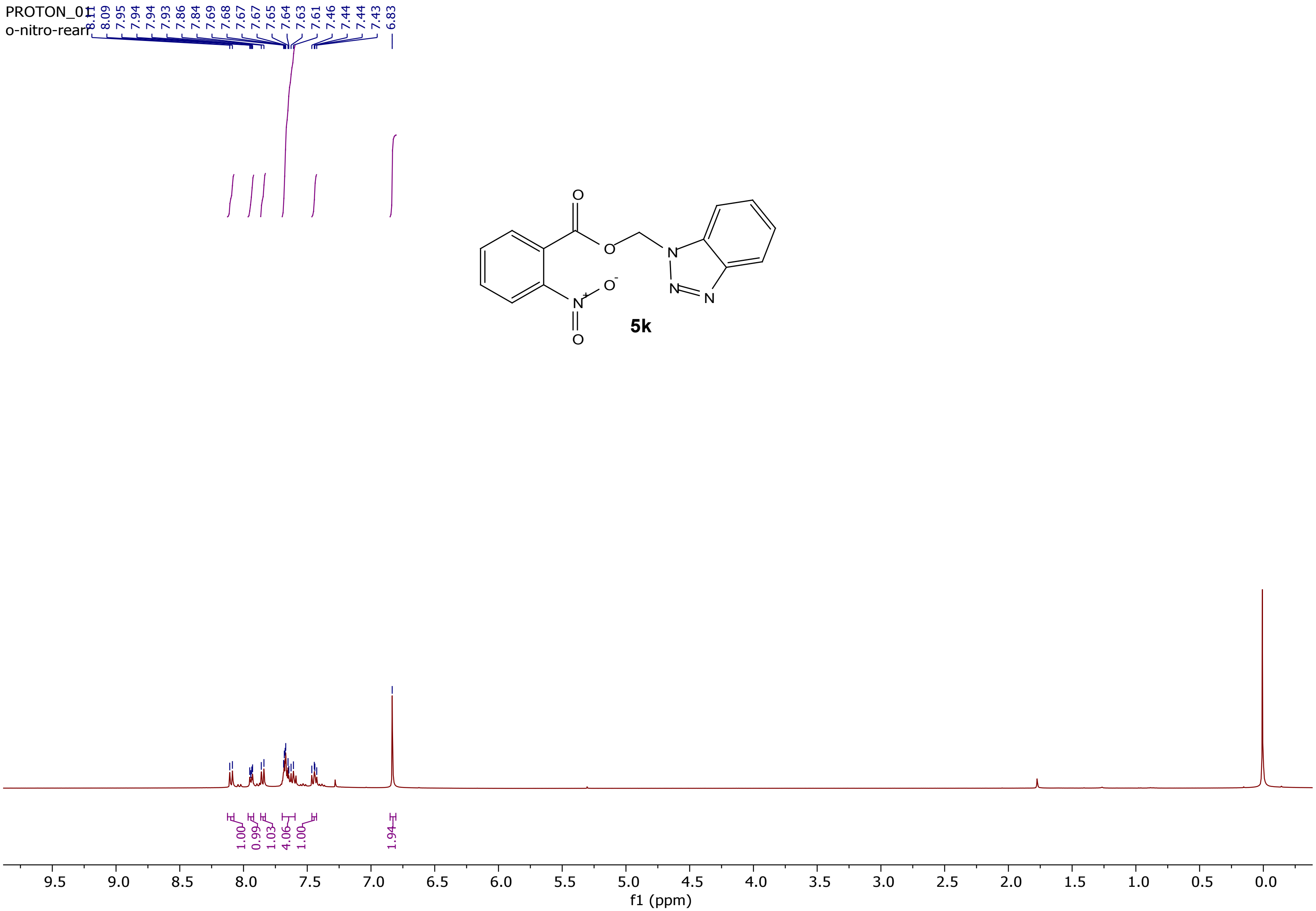

Figure S33. ^{1}H NMR of **5k**.

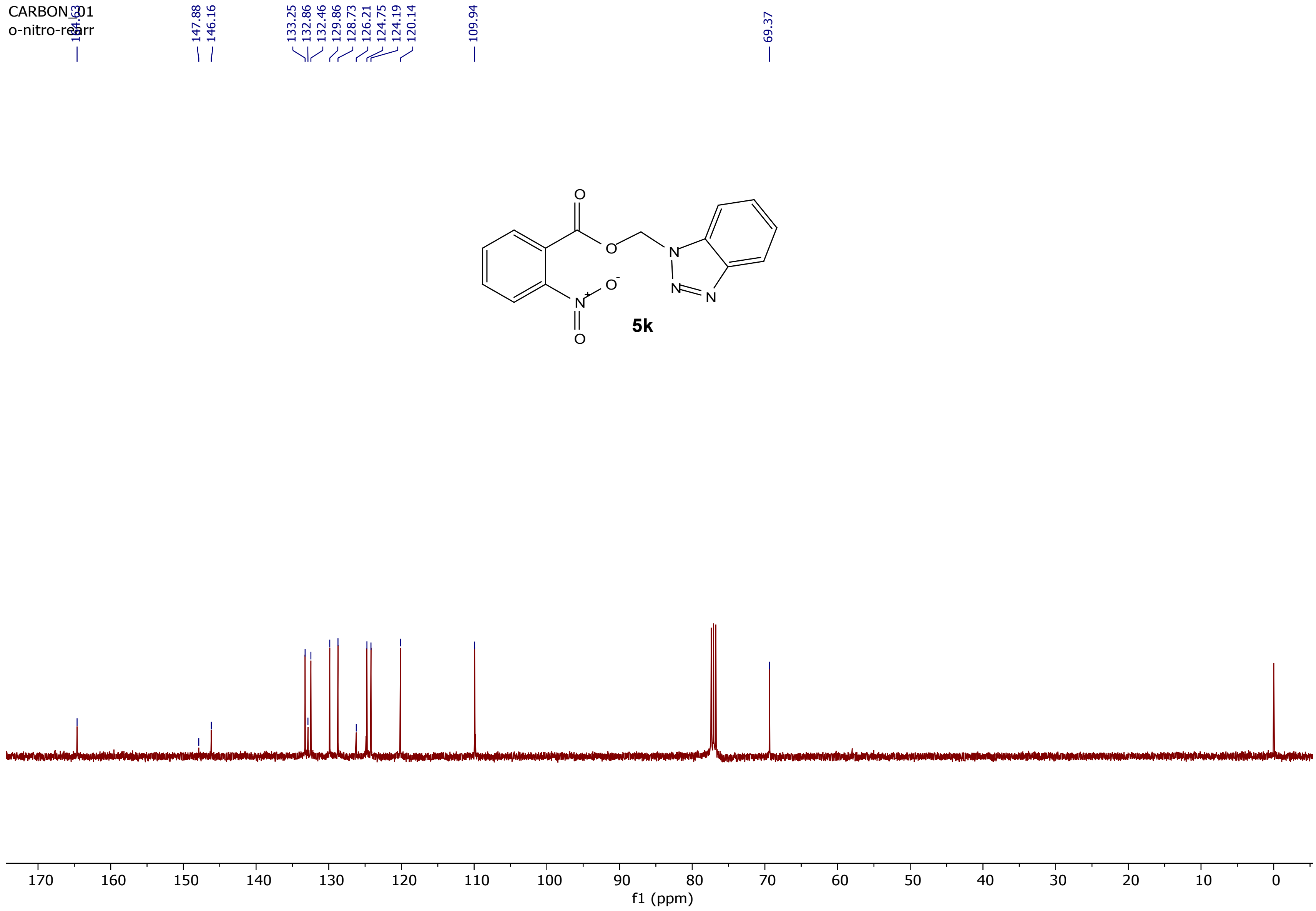

Figure S34. ^{13}C NMR of **5k**.

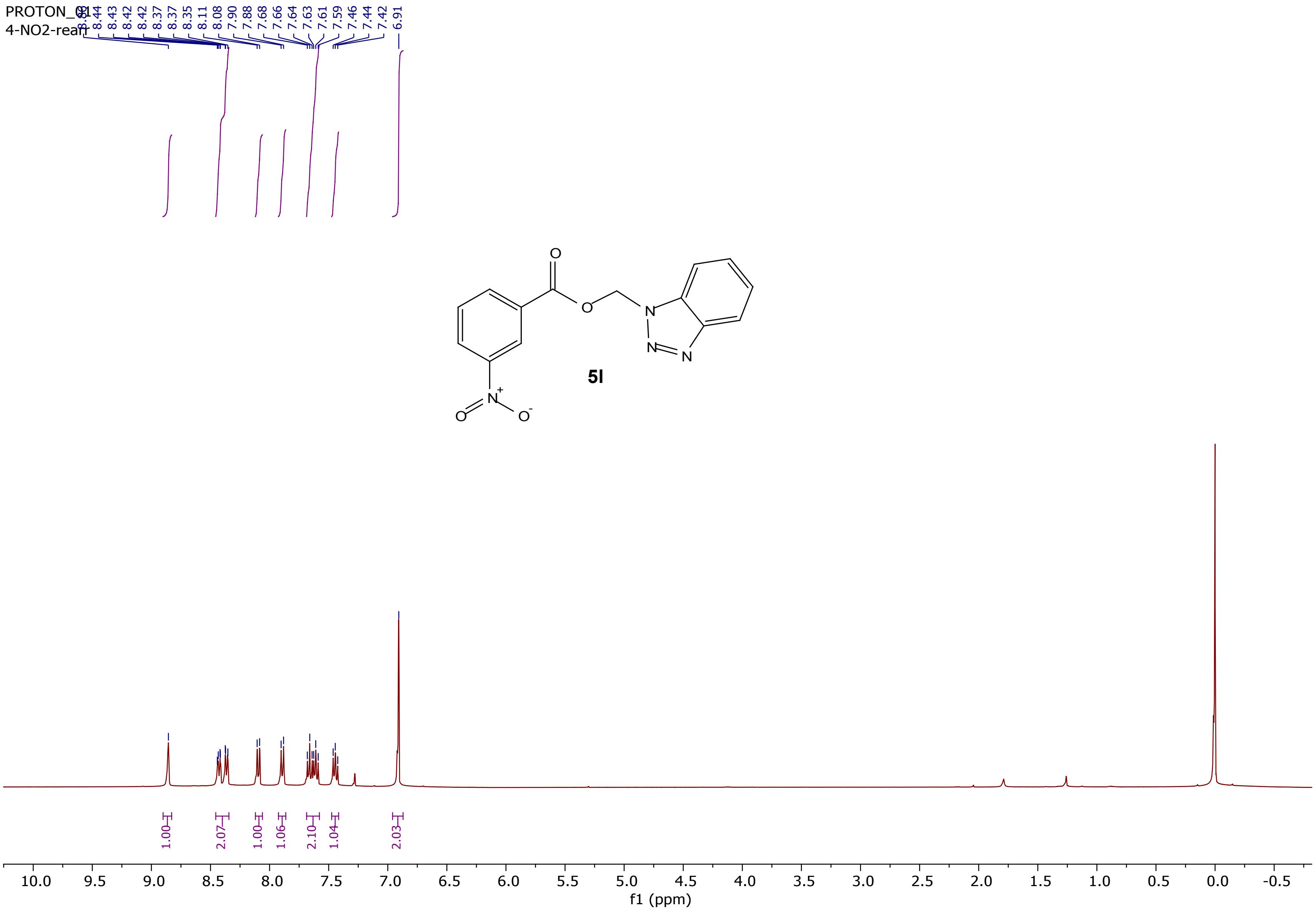

Figure S35. ^{1}H NMR of **5l**.

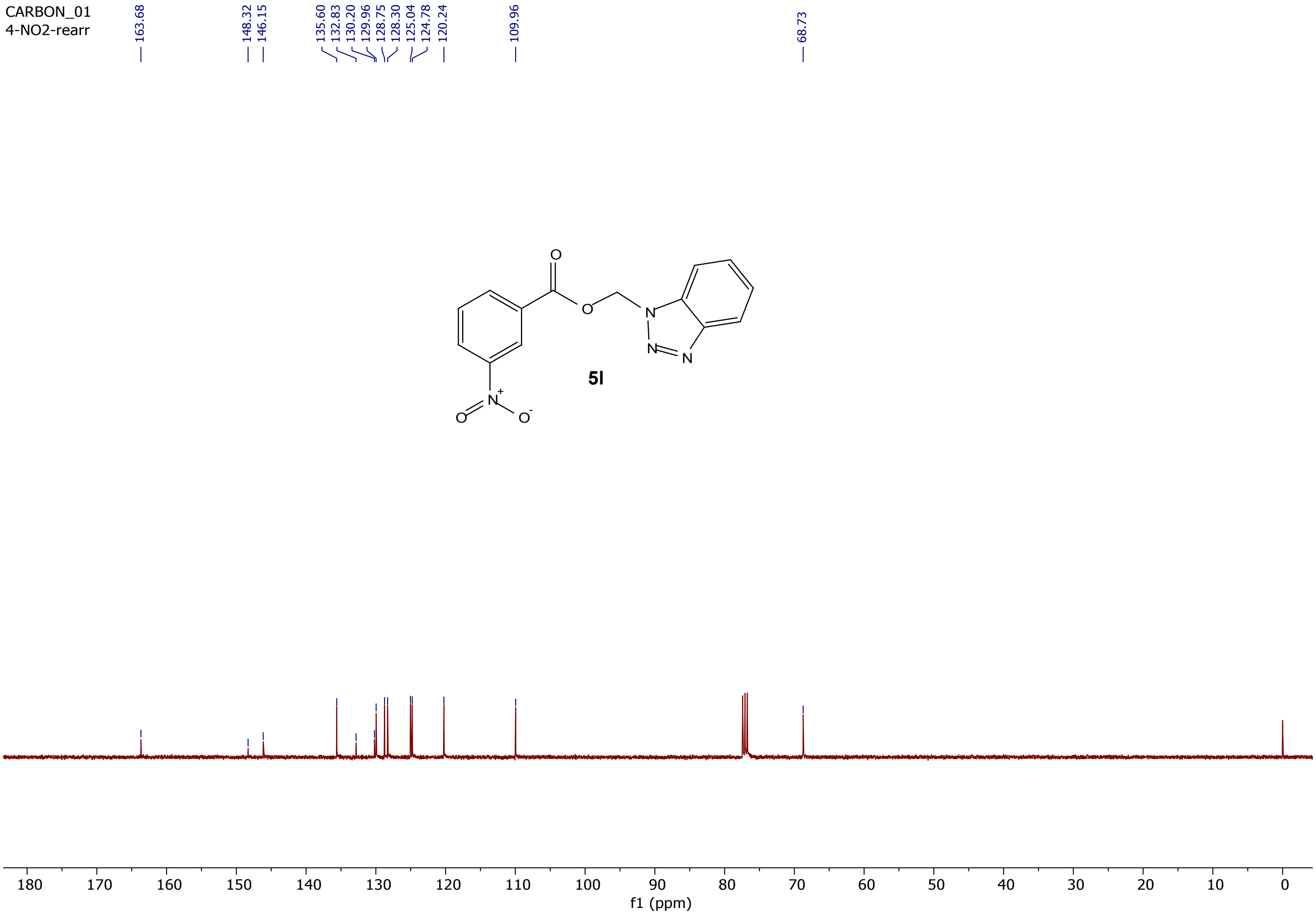

Figure S36. ^{13}C NMR of **5l**.

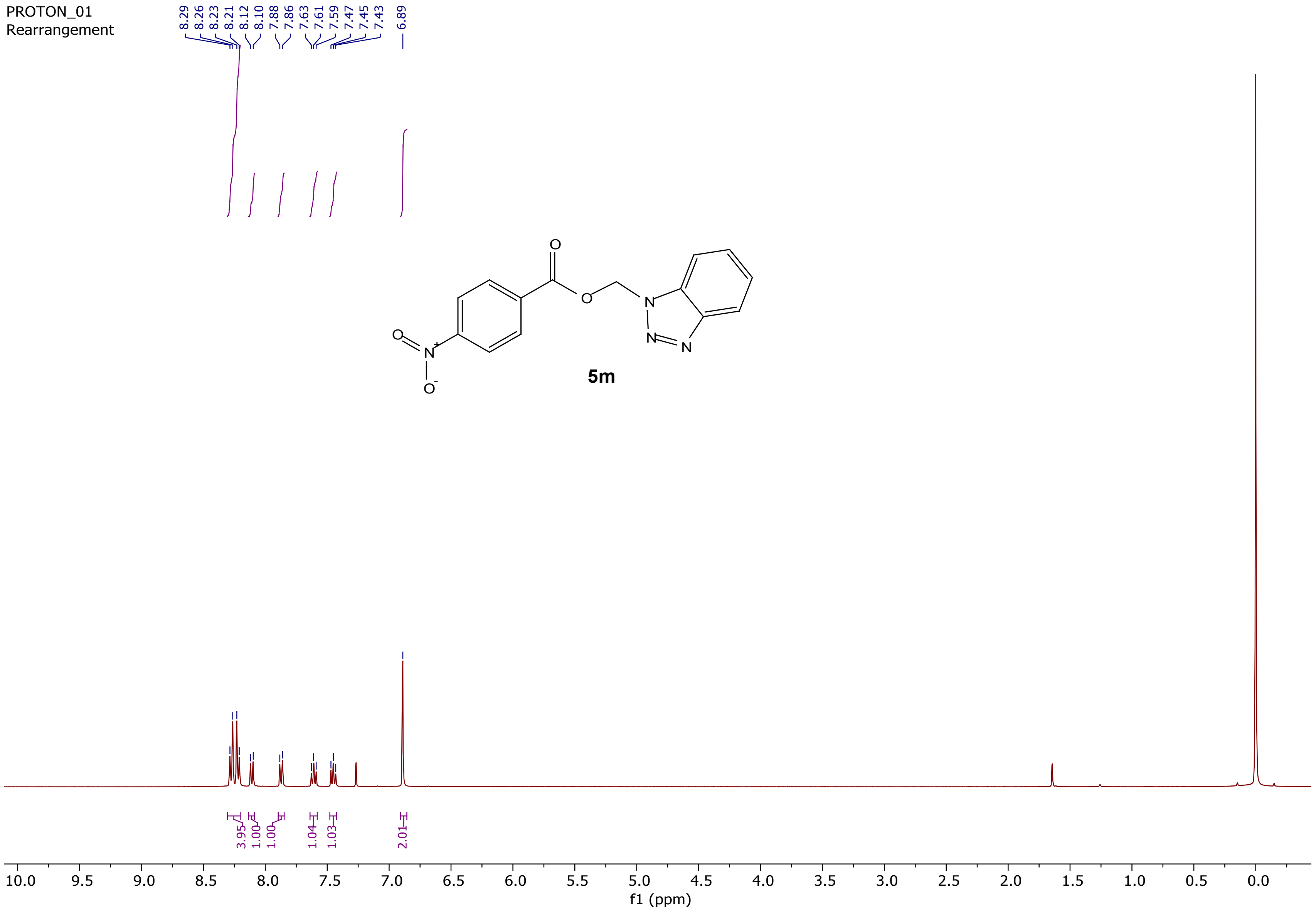

Figure S37. ^{1}H NMR of **5m**.

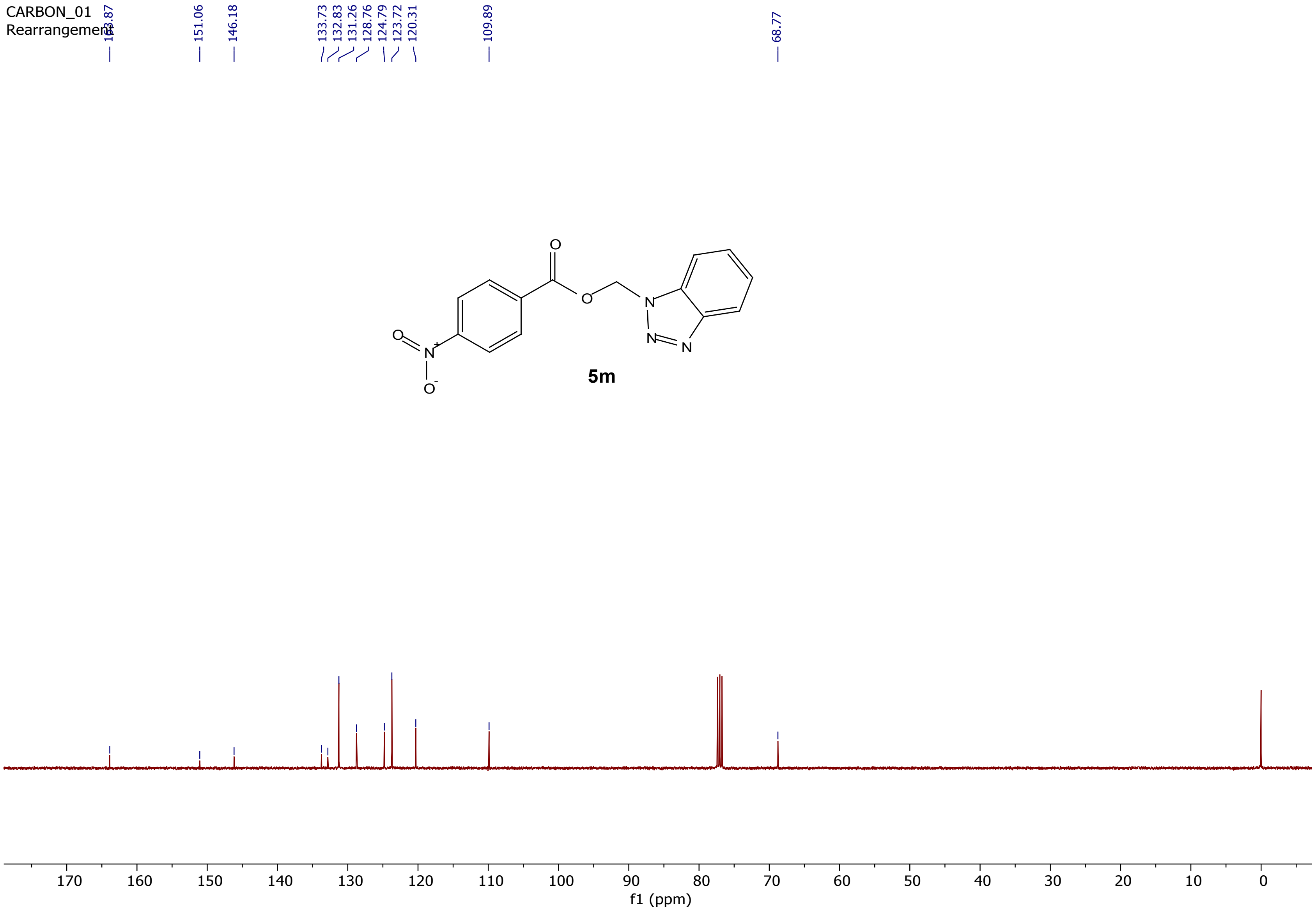

Figure S38. ^{13}C NMR of **5m**.

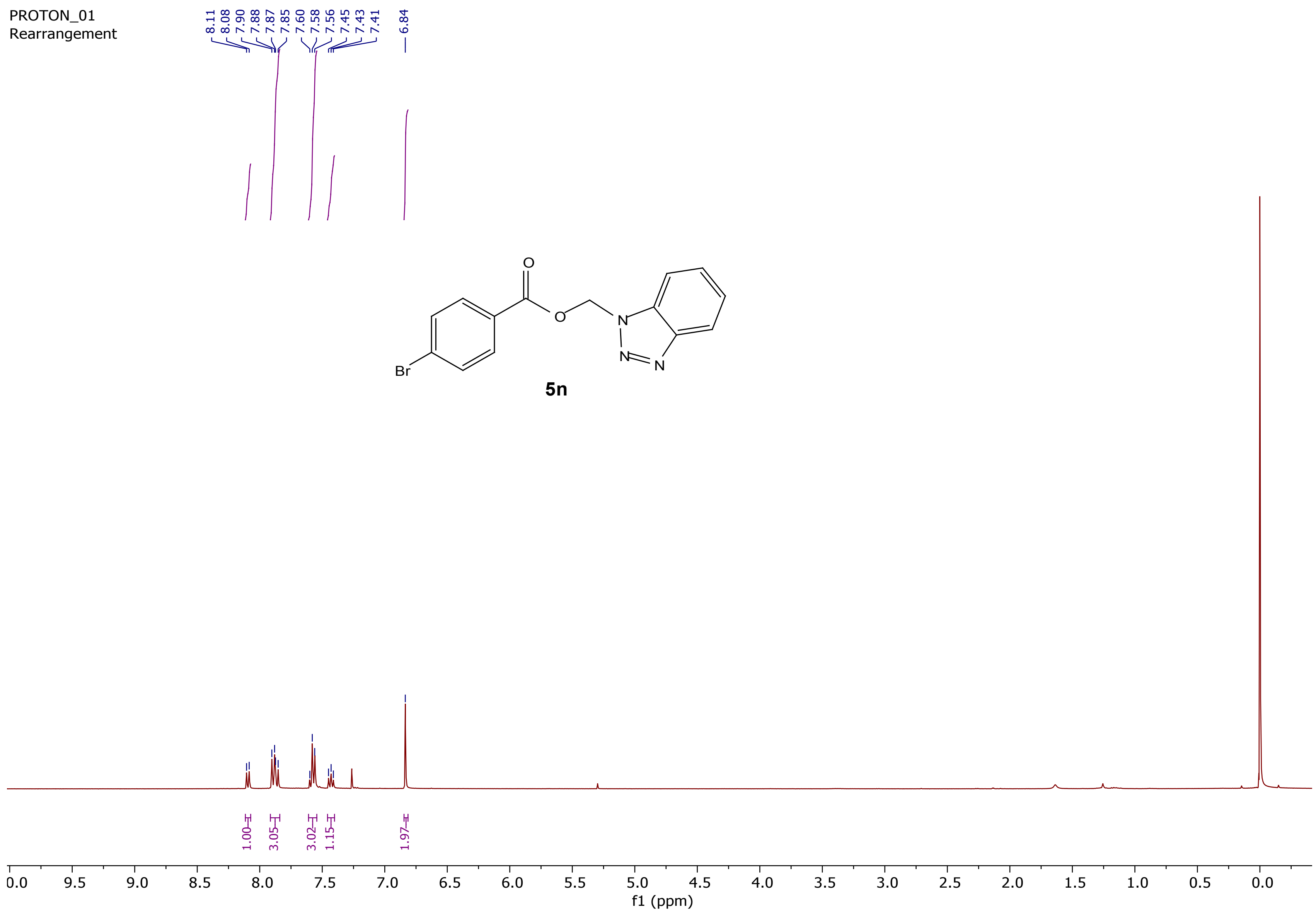

Figure S39. ^{1}H NMR of **5n**.

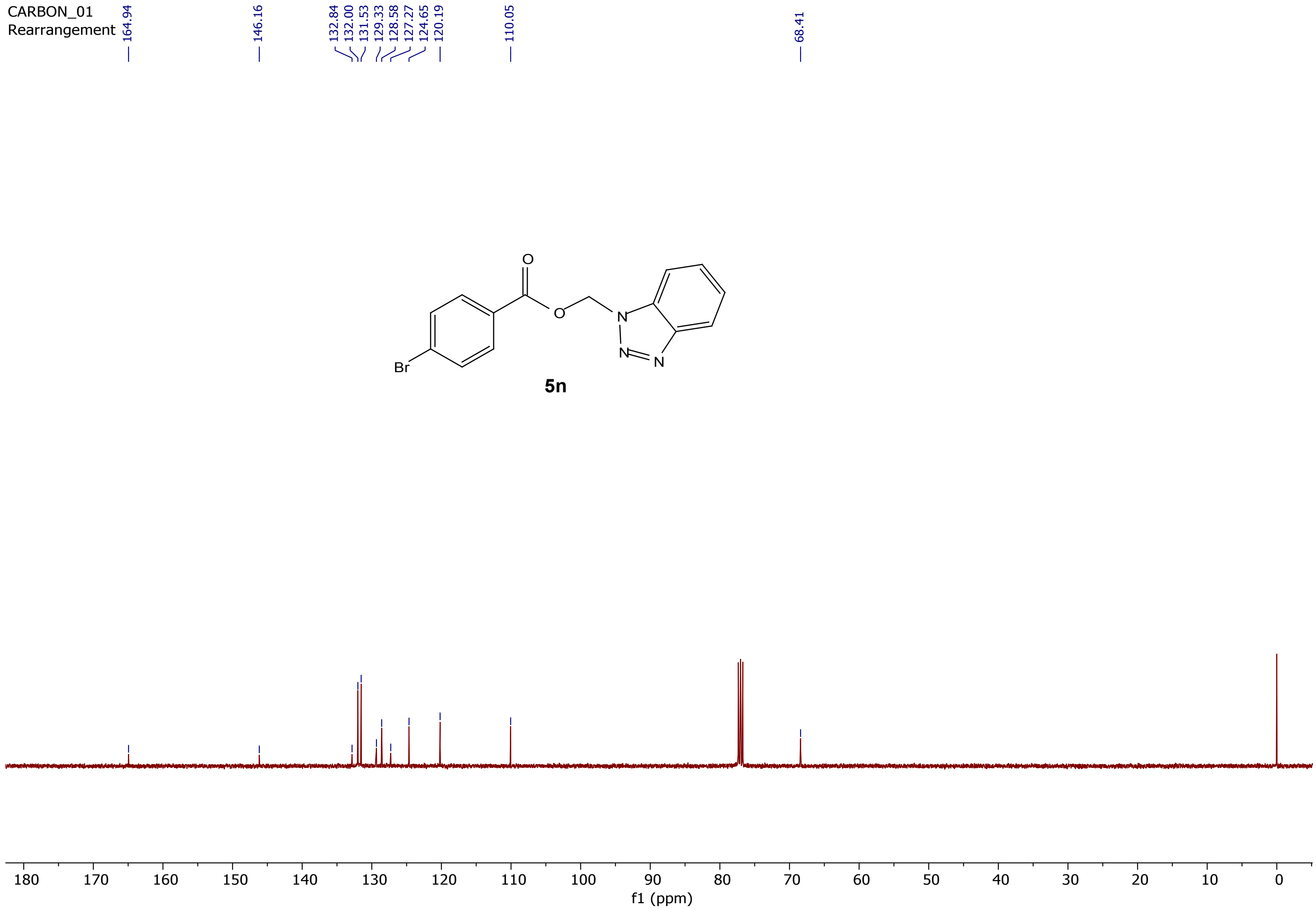

Figure S40. ^{13}C NMR of **5n**.

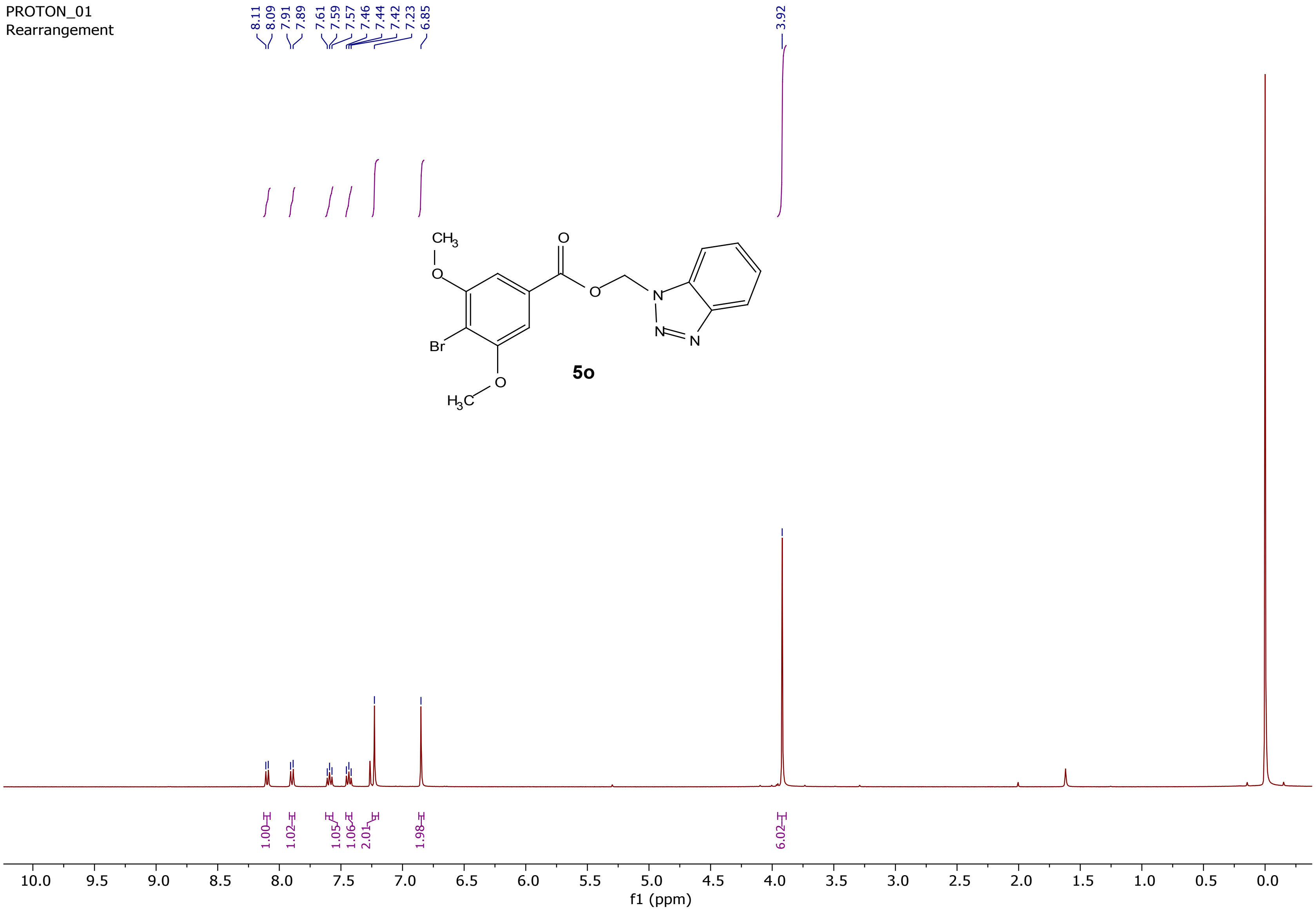

Figure S41. ^{1}H NMR of **5o**.

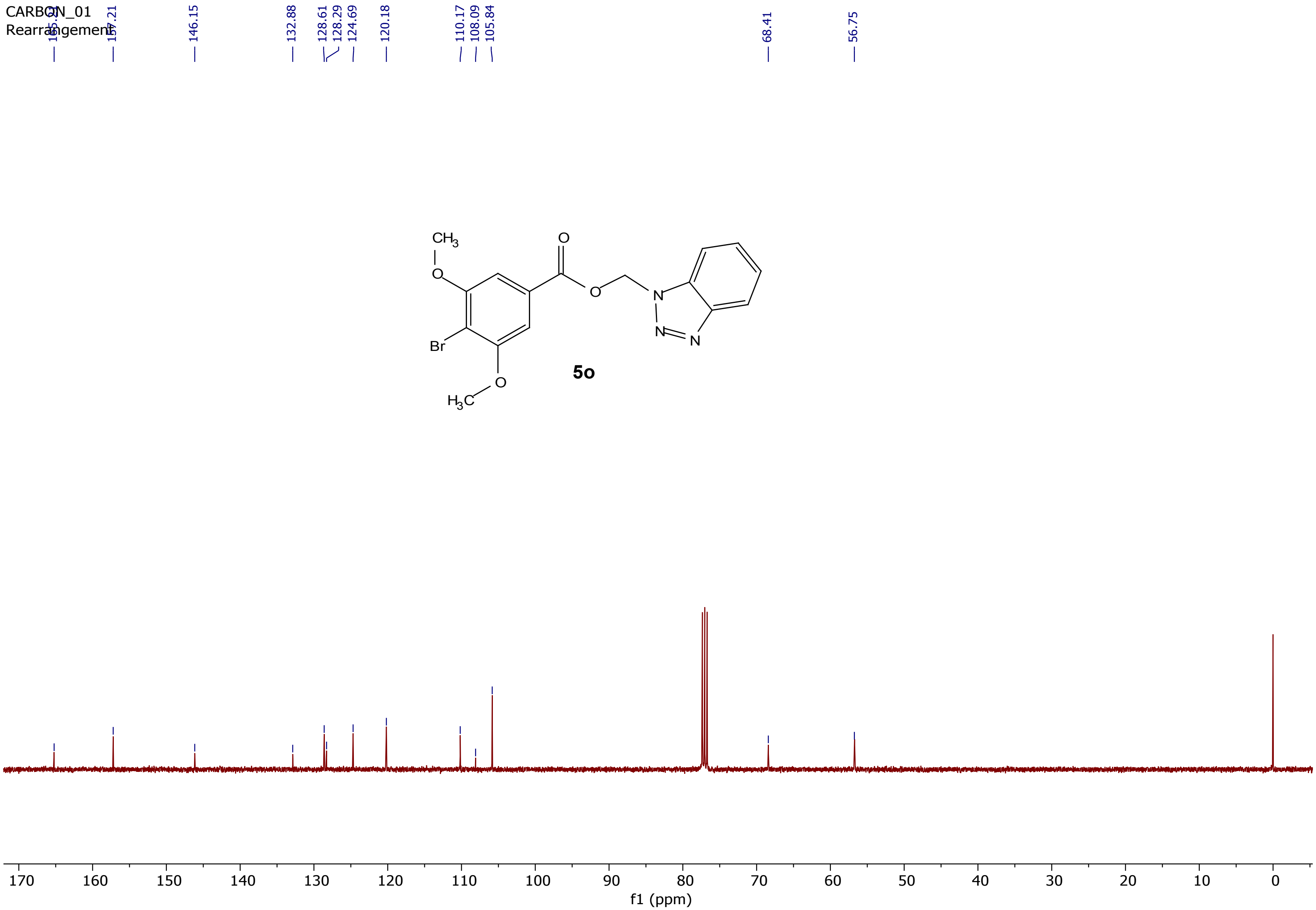

Figure S42. ^{13}C NMR of **5o**.

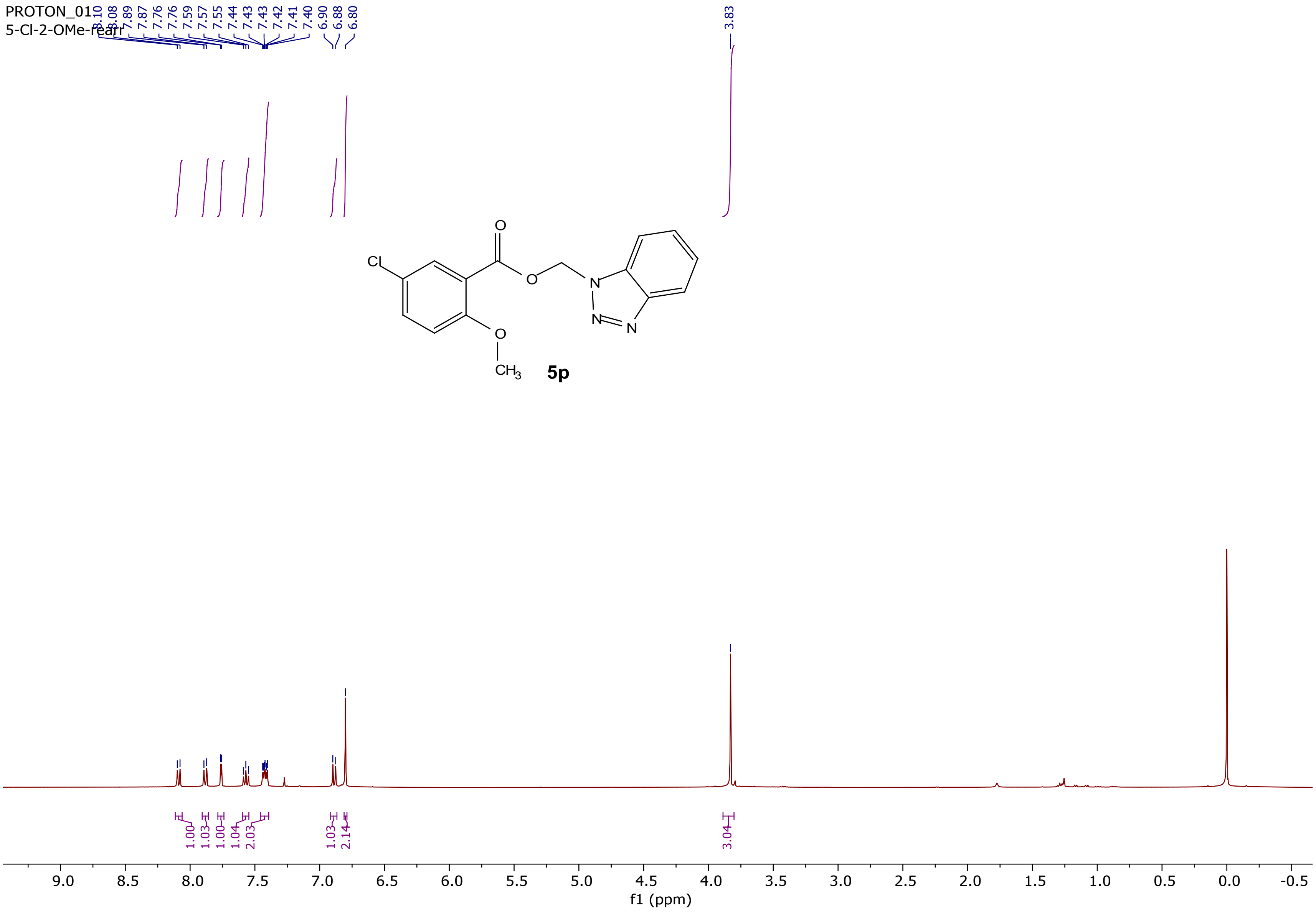

Figure S43. ^{1}H NMR of **5p**.

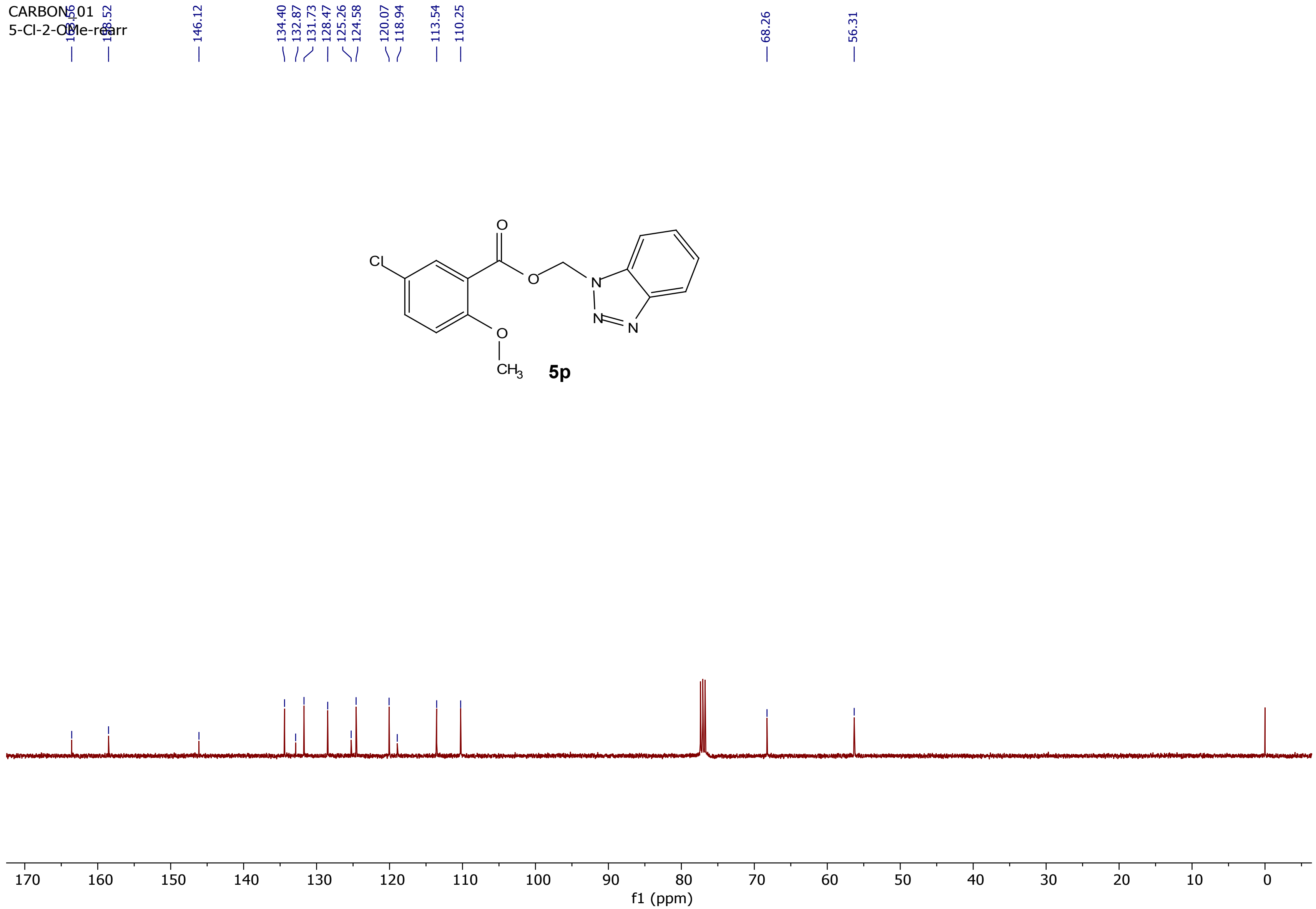

Figure S44. ^{13}C NMR of **5p**.

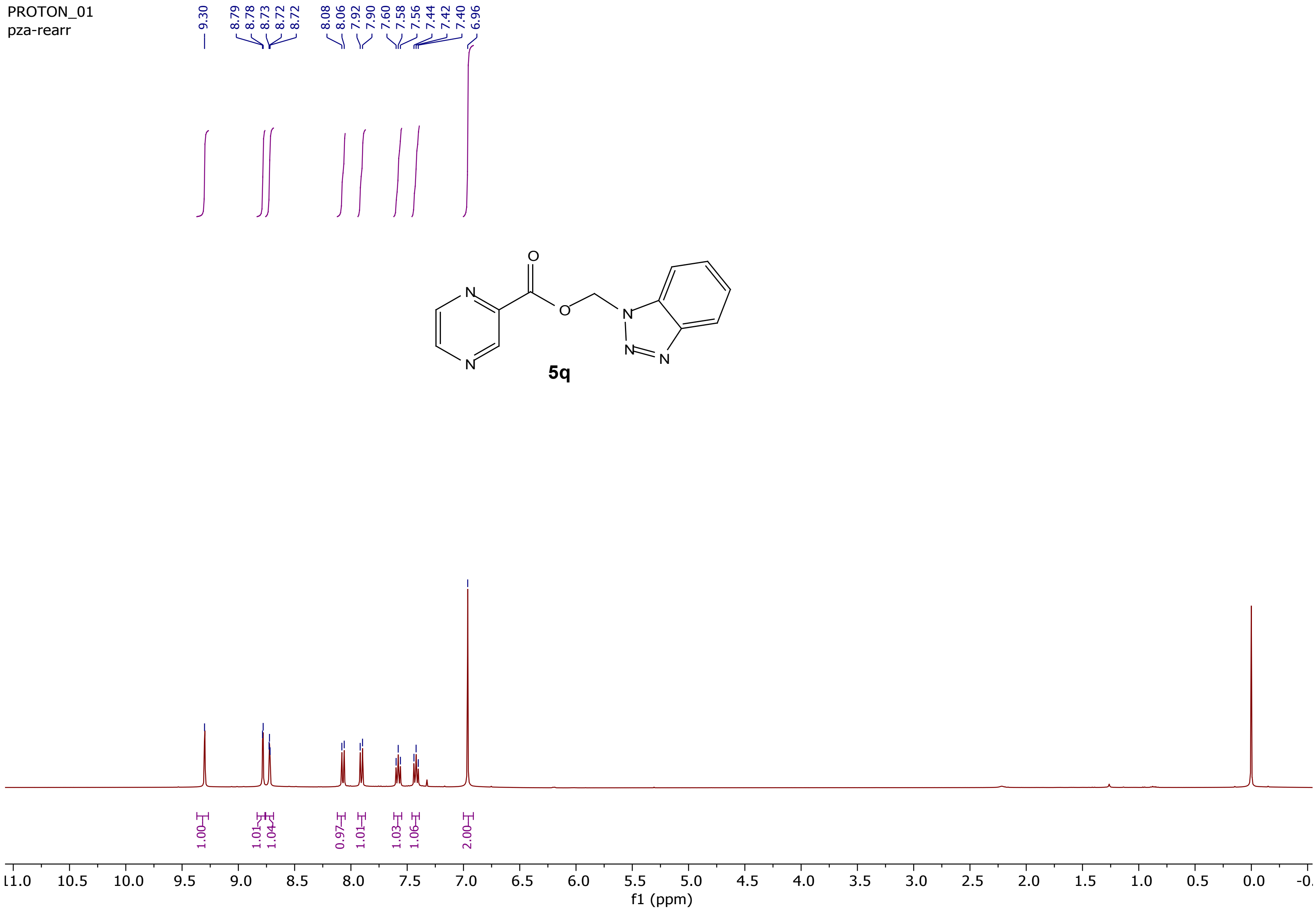

Figure S45. ^{1}H NMR of **5q**.

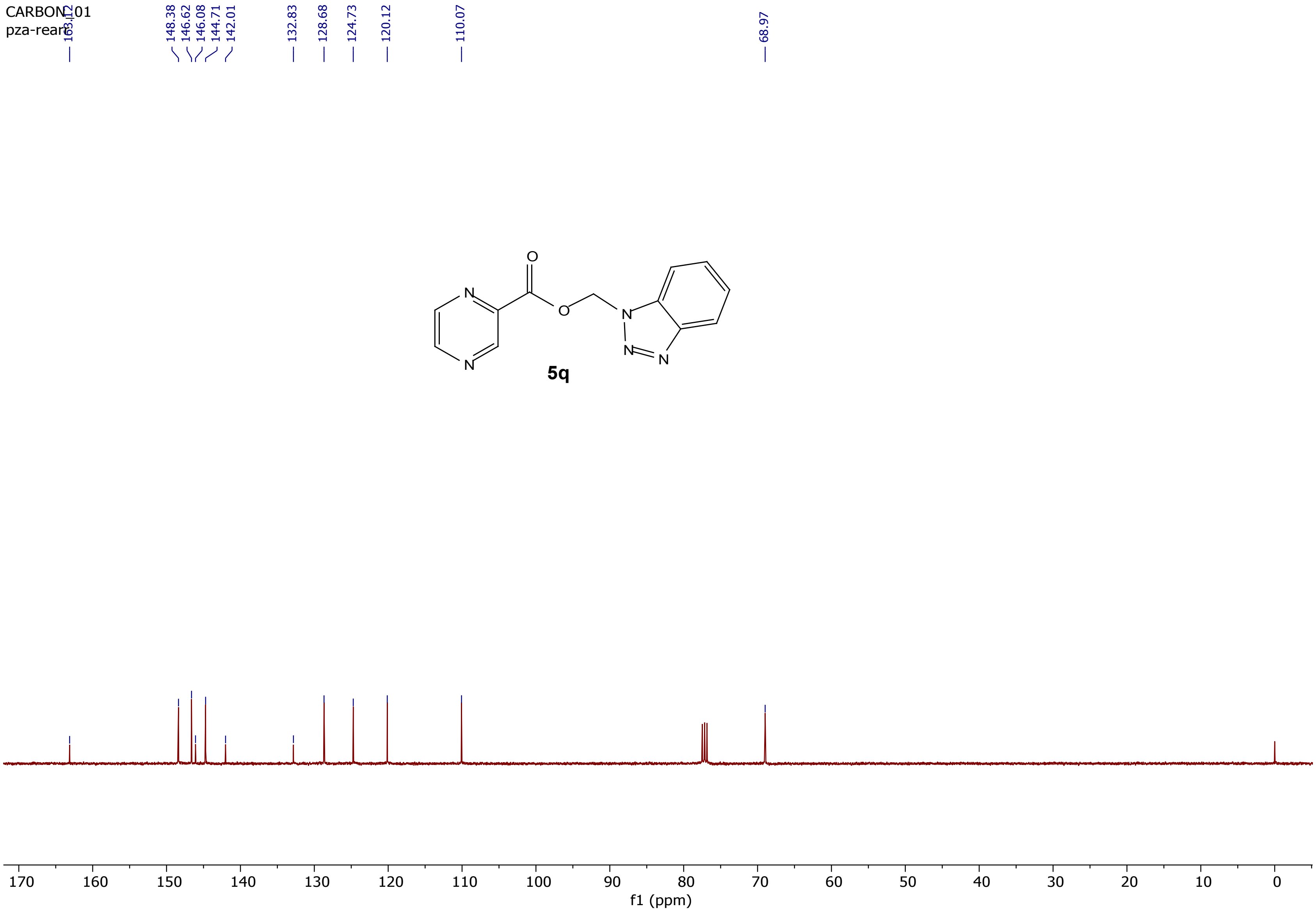

Figure S46. ^{13}C NMR of **5q**.

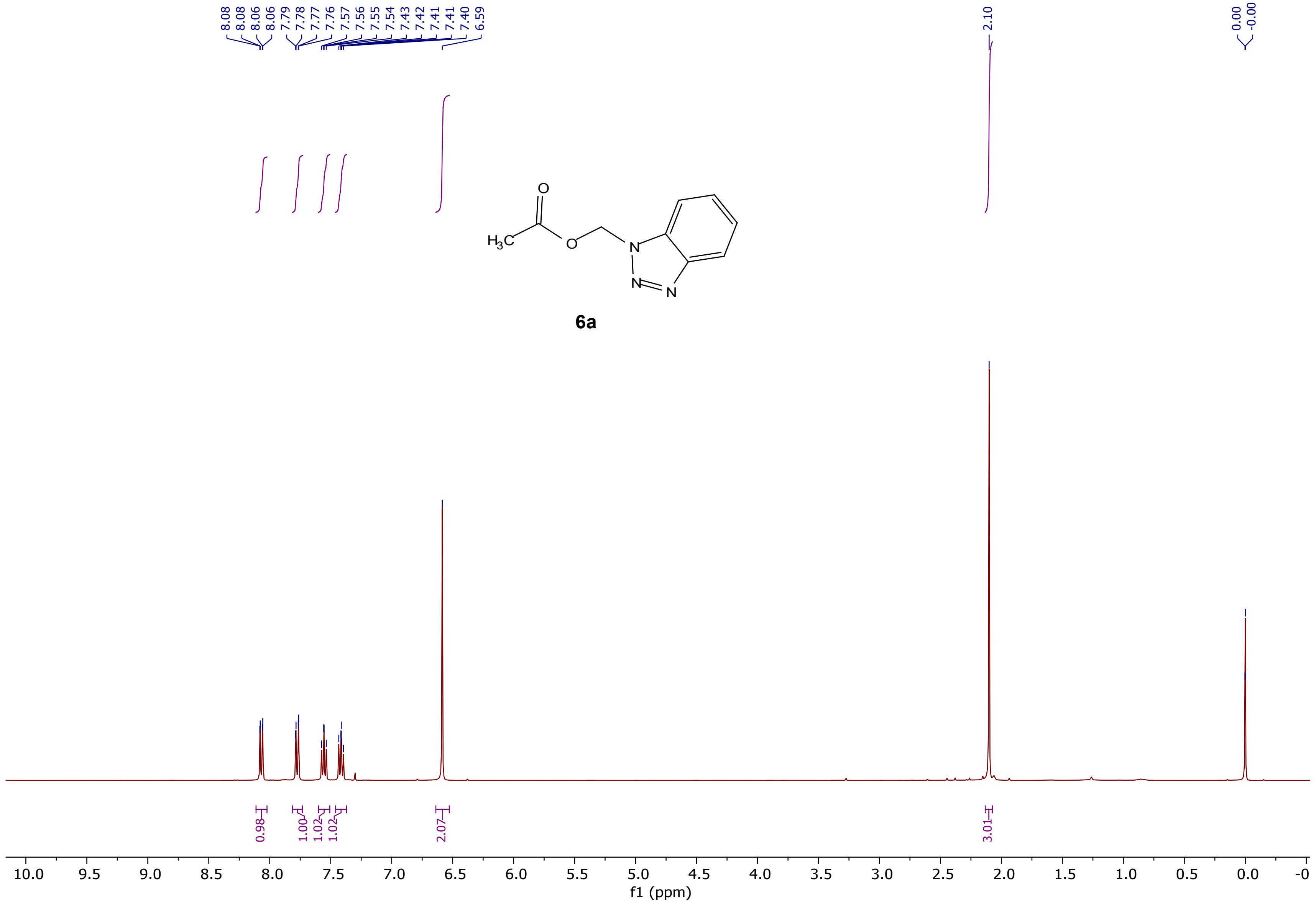

Figure S47. ^{1}H NMR of **6a**.

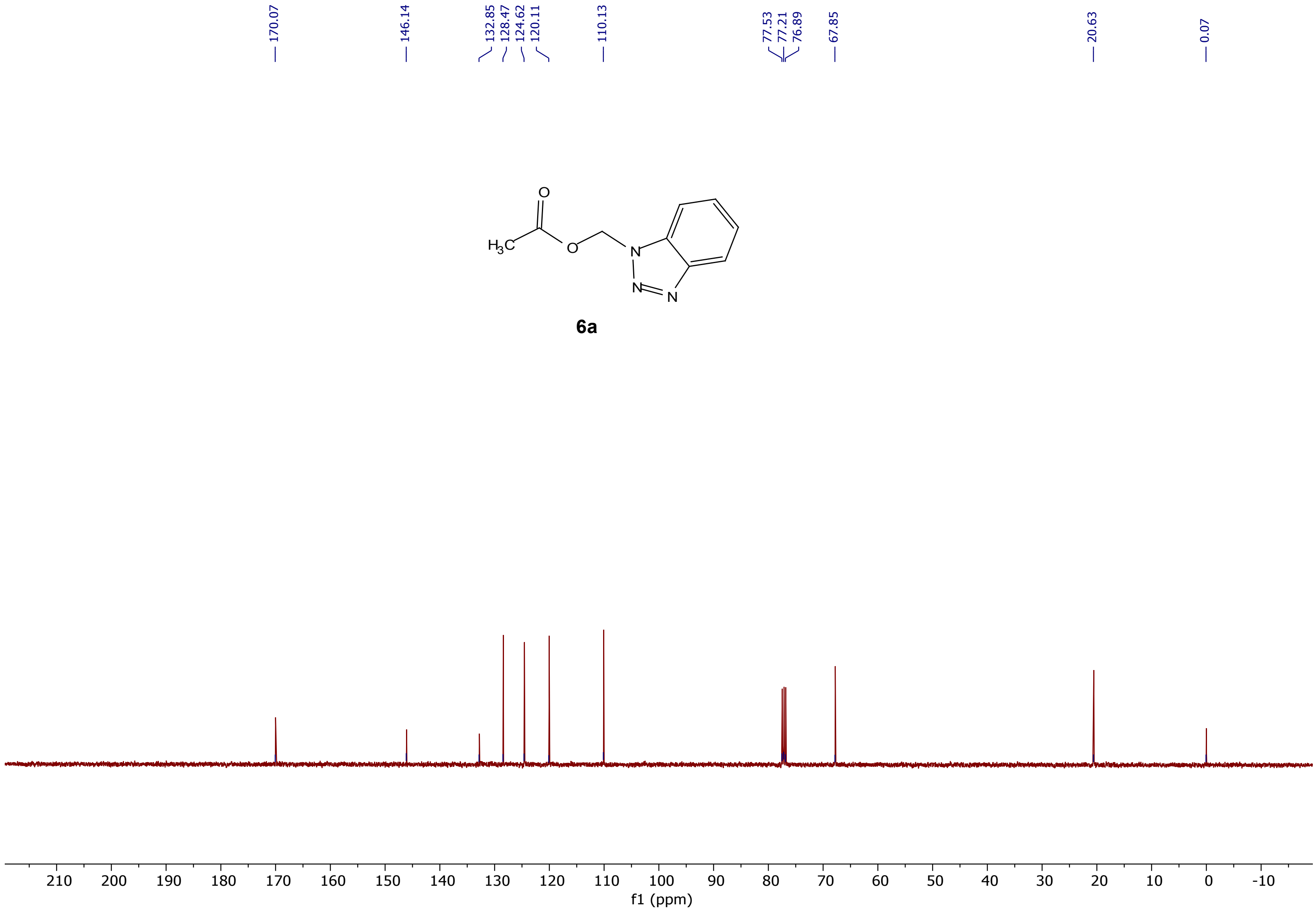

Figure S48. ^{13}C NMR of **6a**.

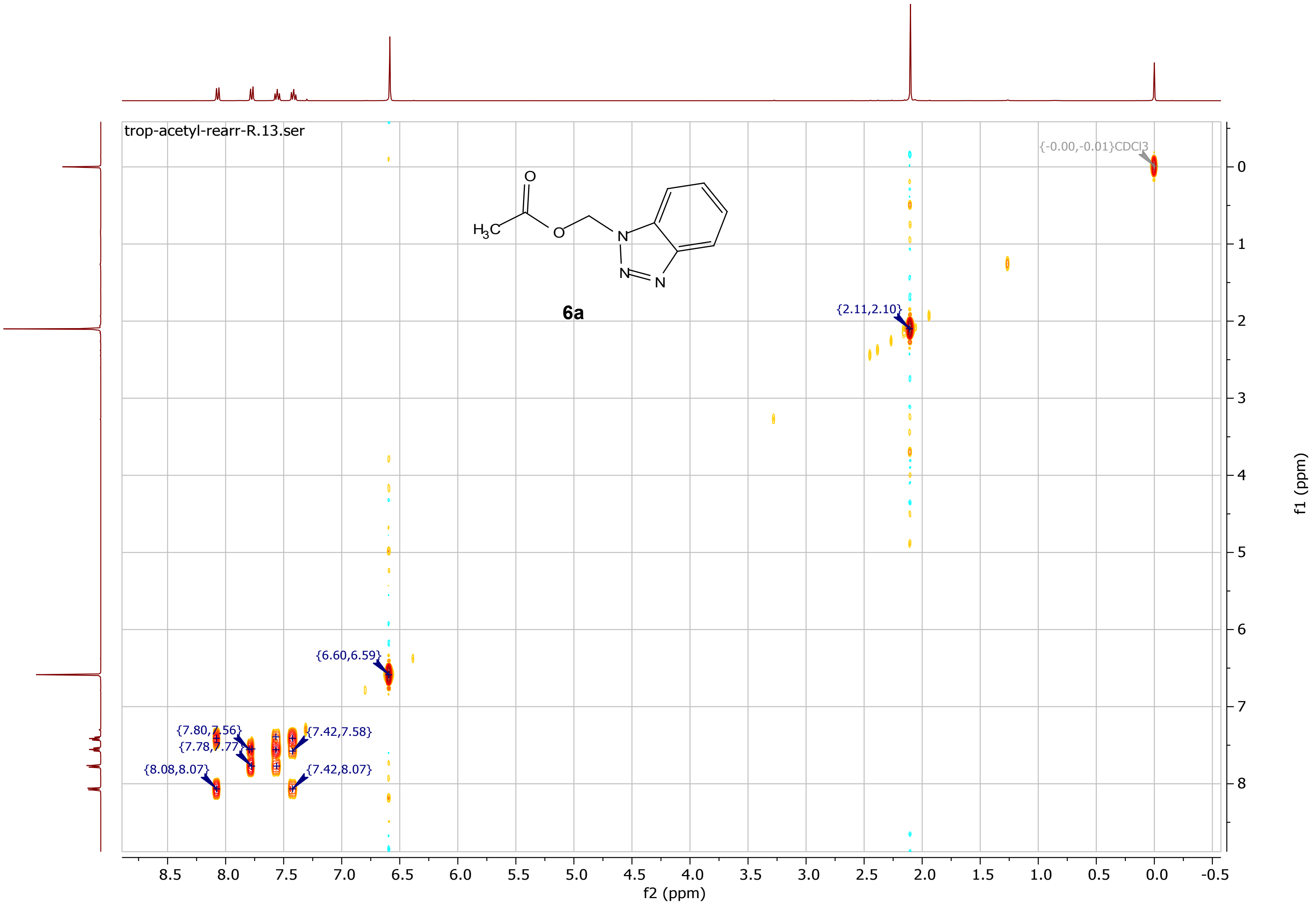

Figure S49. ^{1}H-^{1}H COSY of **6a**.

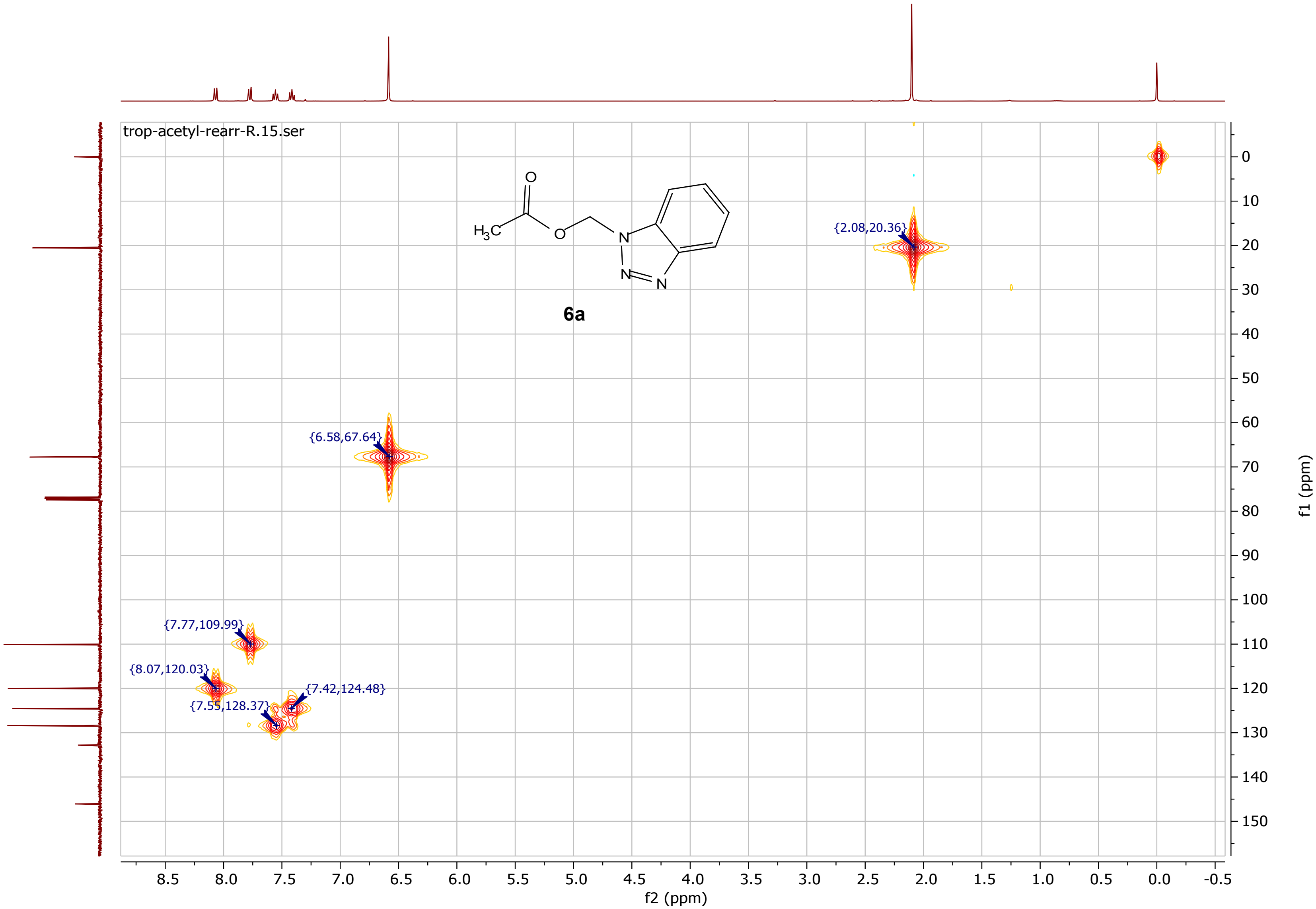

Figure S50. ^{1}H-^{13}C HMQC of **6a**.

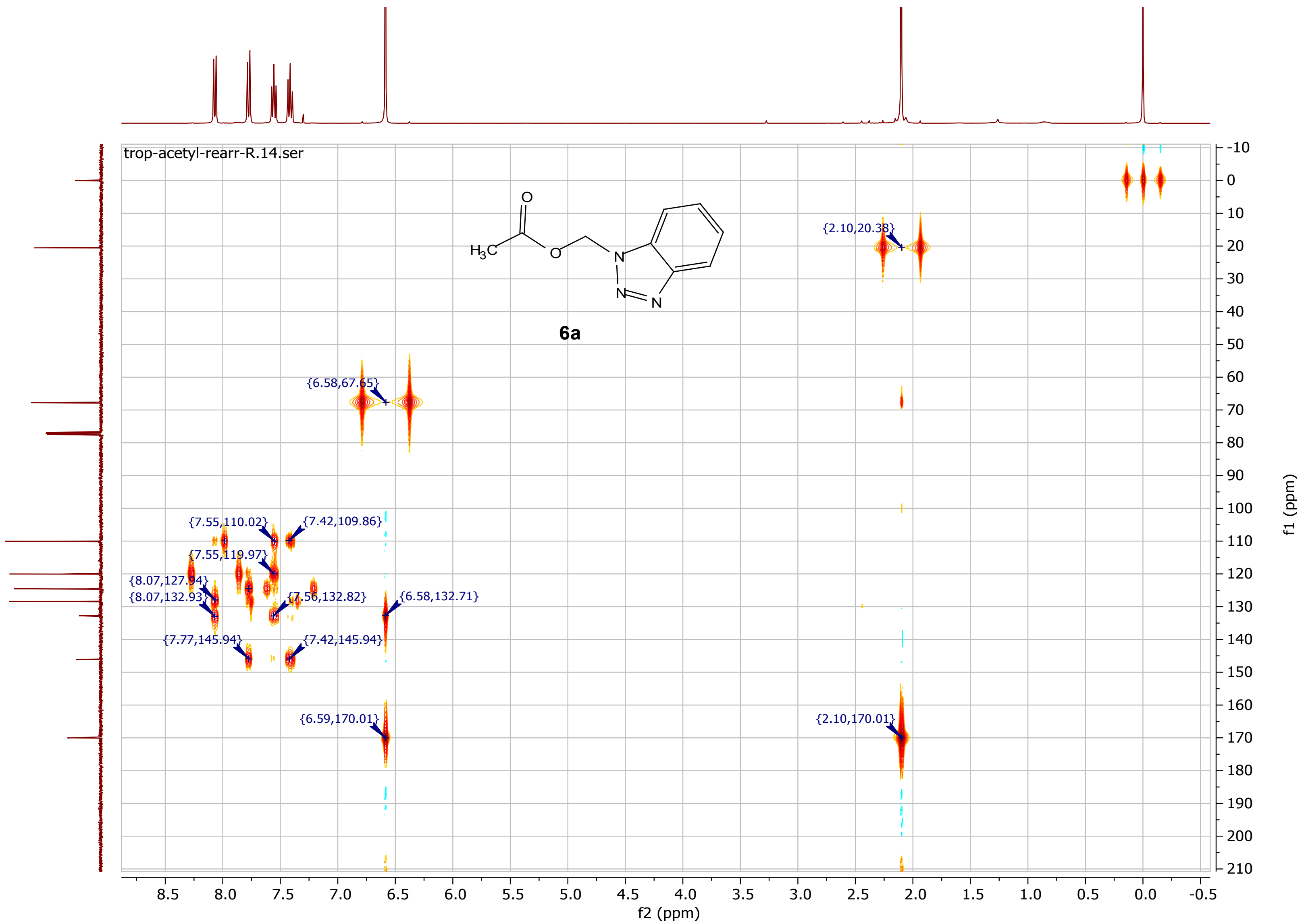

Figure S51. ^{1}H-^{13}C HMBC of **6a**.

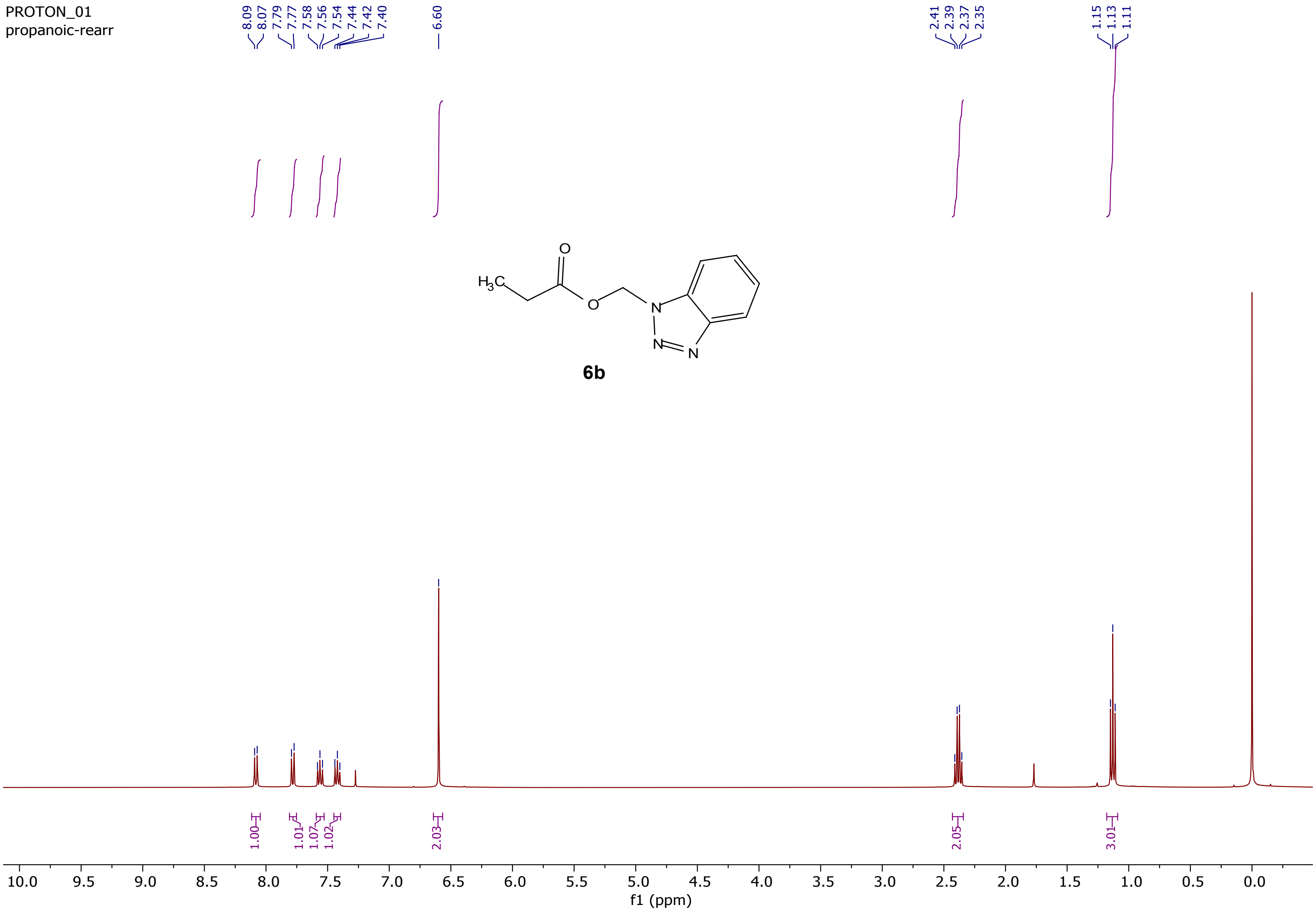

Figure S52. ^{1}H NMR of **6b**.

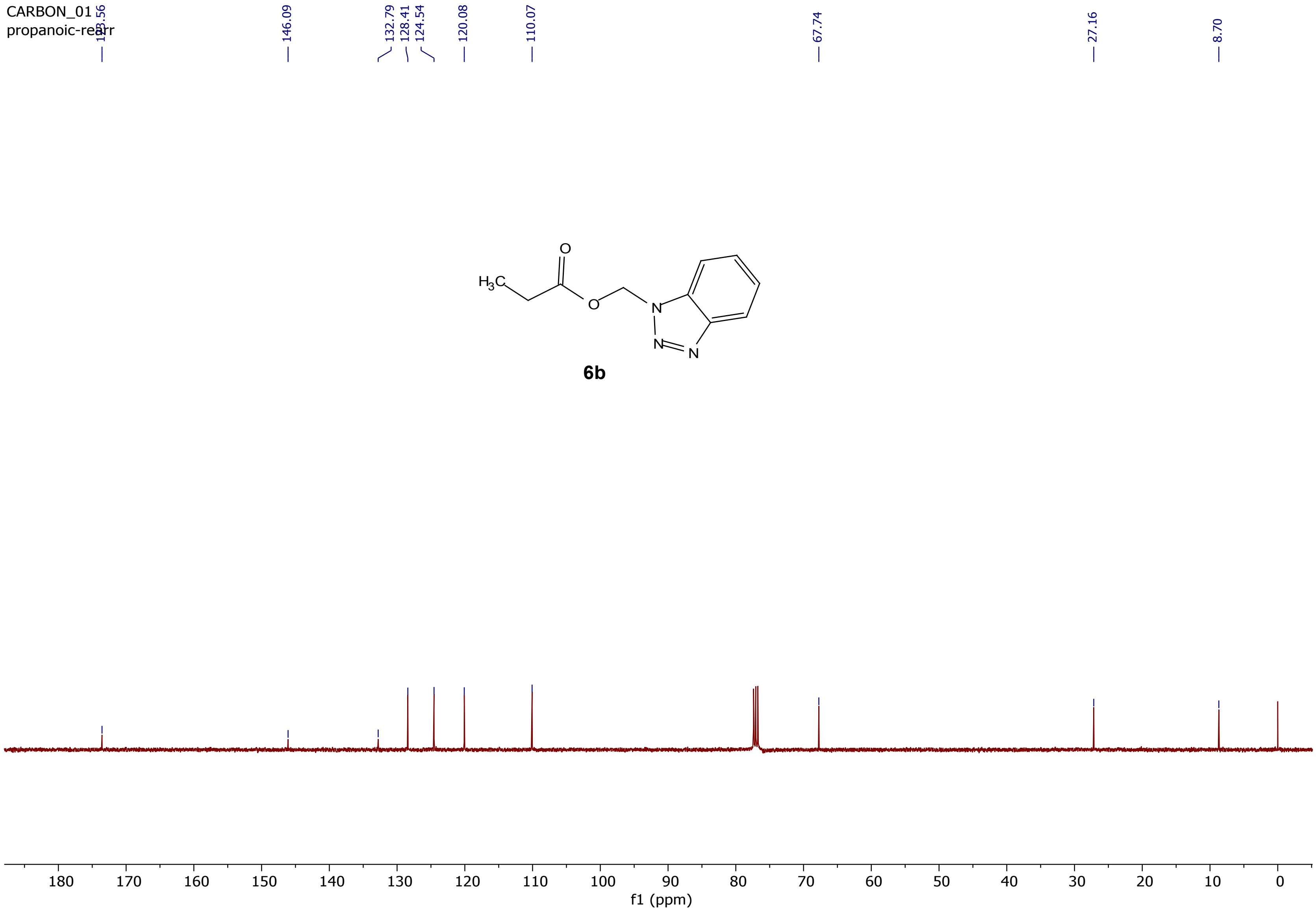

Figure S53. ^{13}C NMR of **6b**.

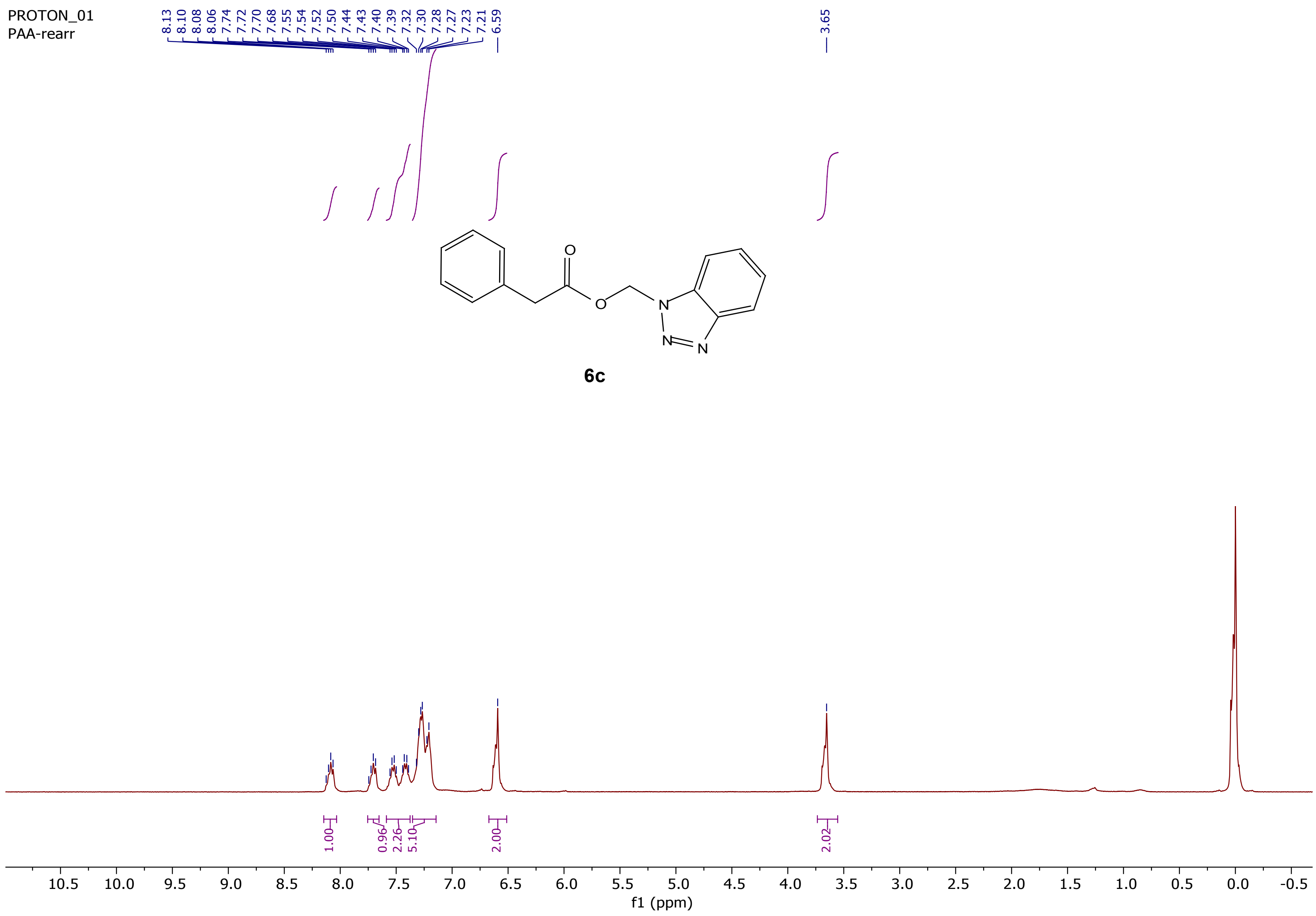

Figure S54. ^{1}H NMR of **6c**.

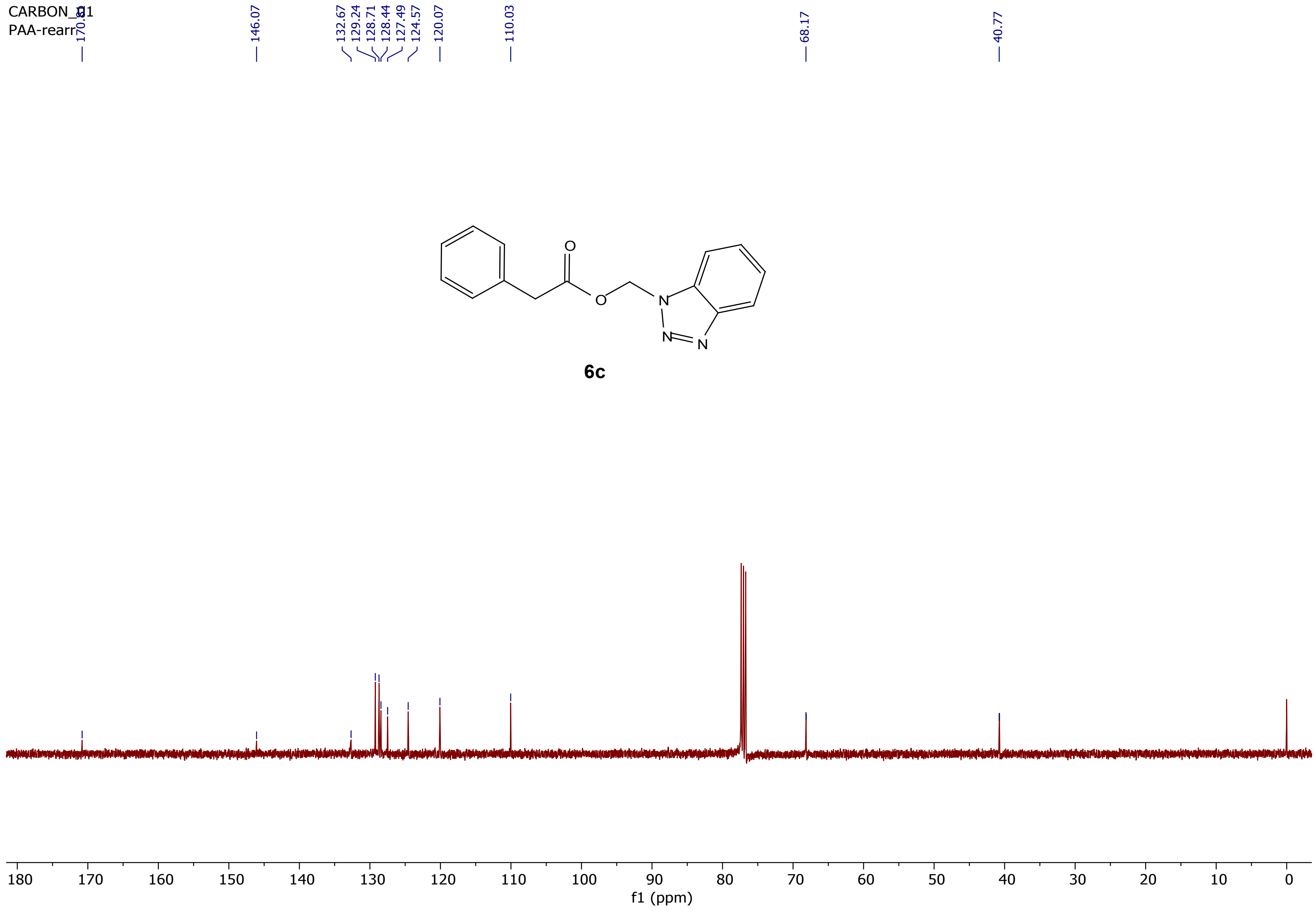

Figure S55. ^{13}C NMR of **6c**.

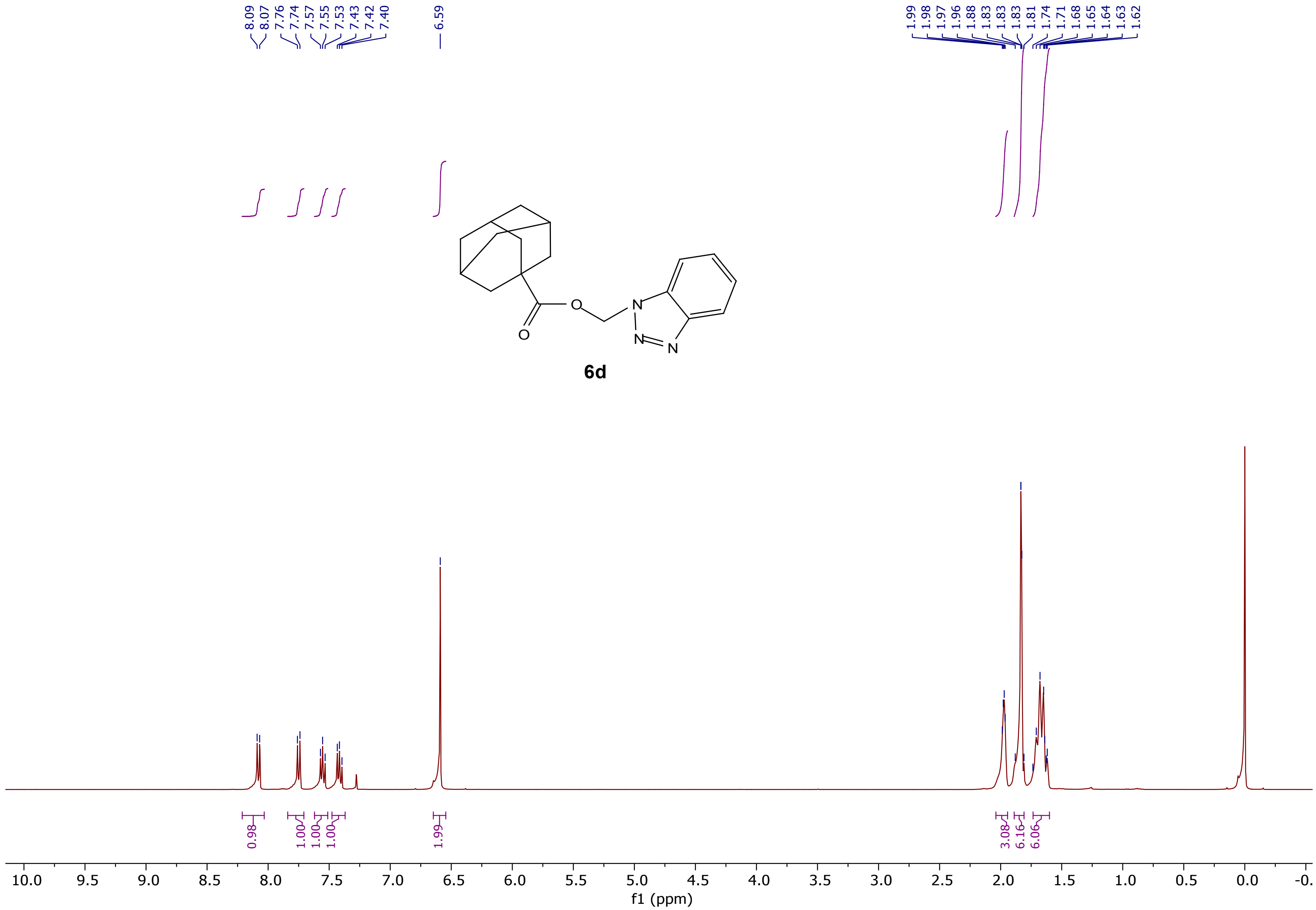

Figure S56. ^{1}H NMR of **6d**.

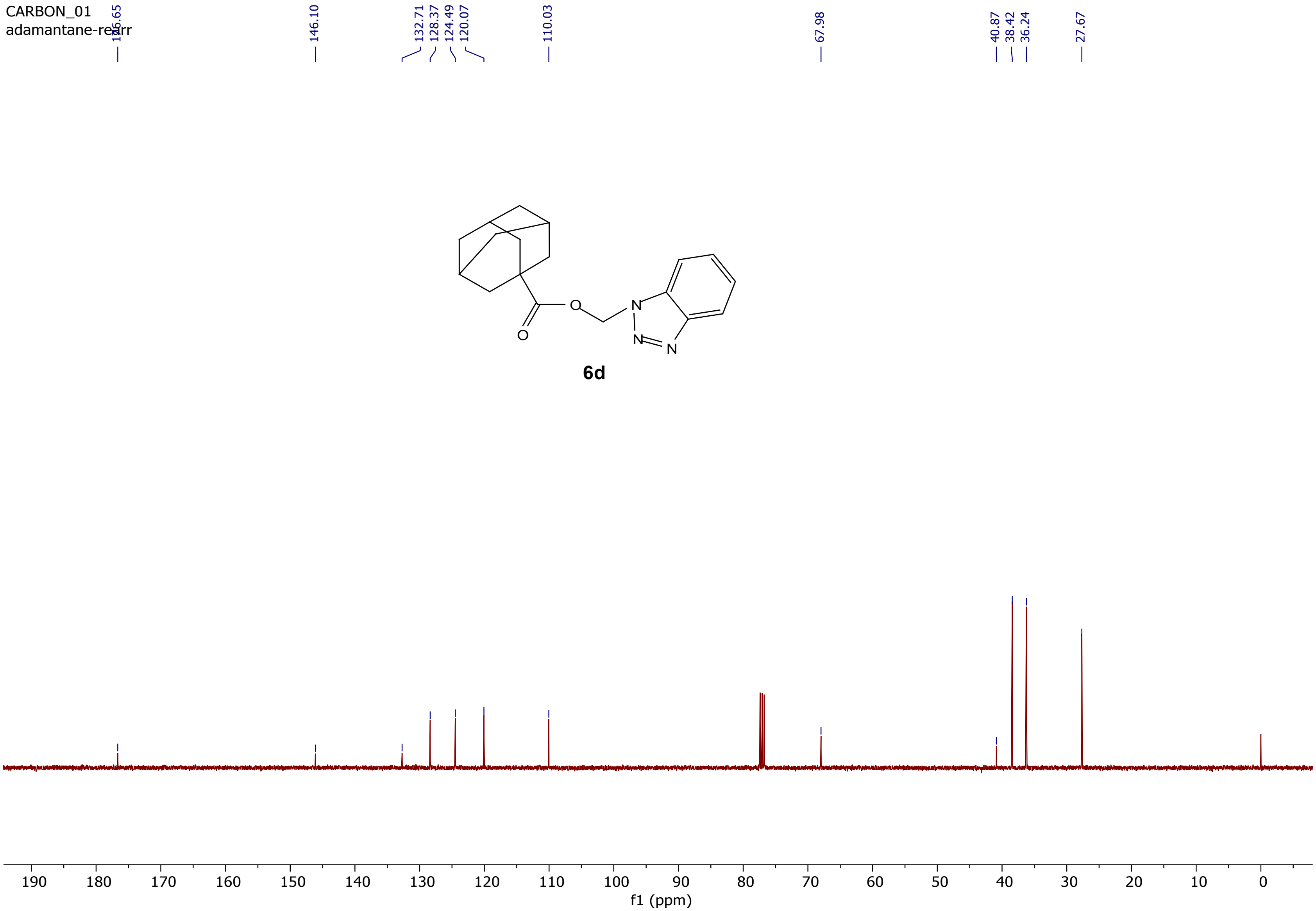

Figure S57. ^{13}C NMR of **6d**.

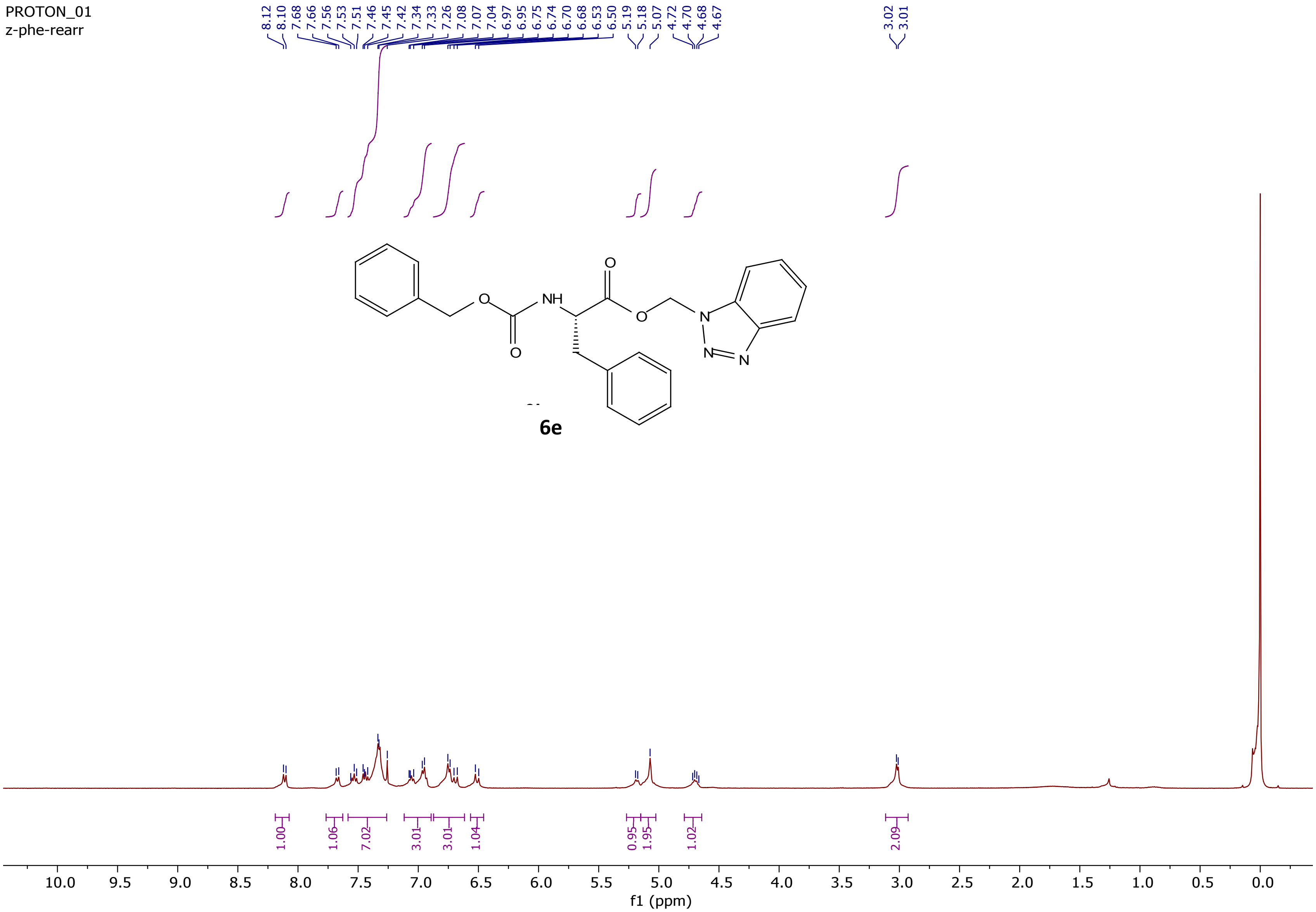

Figure S58. ^{1}H NMR of **6e**.

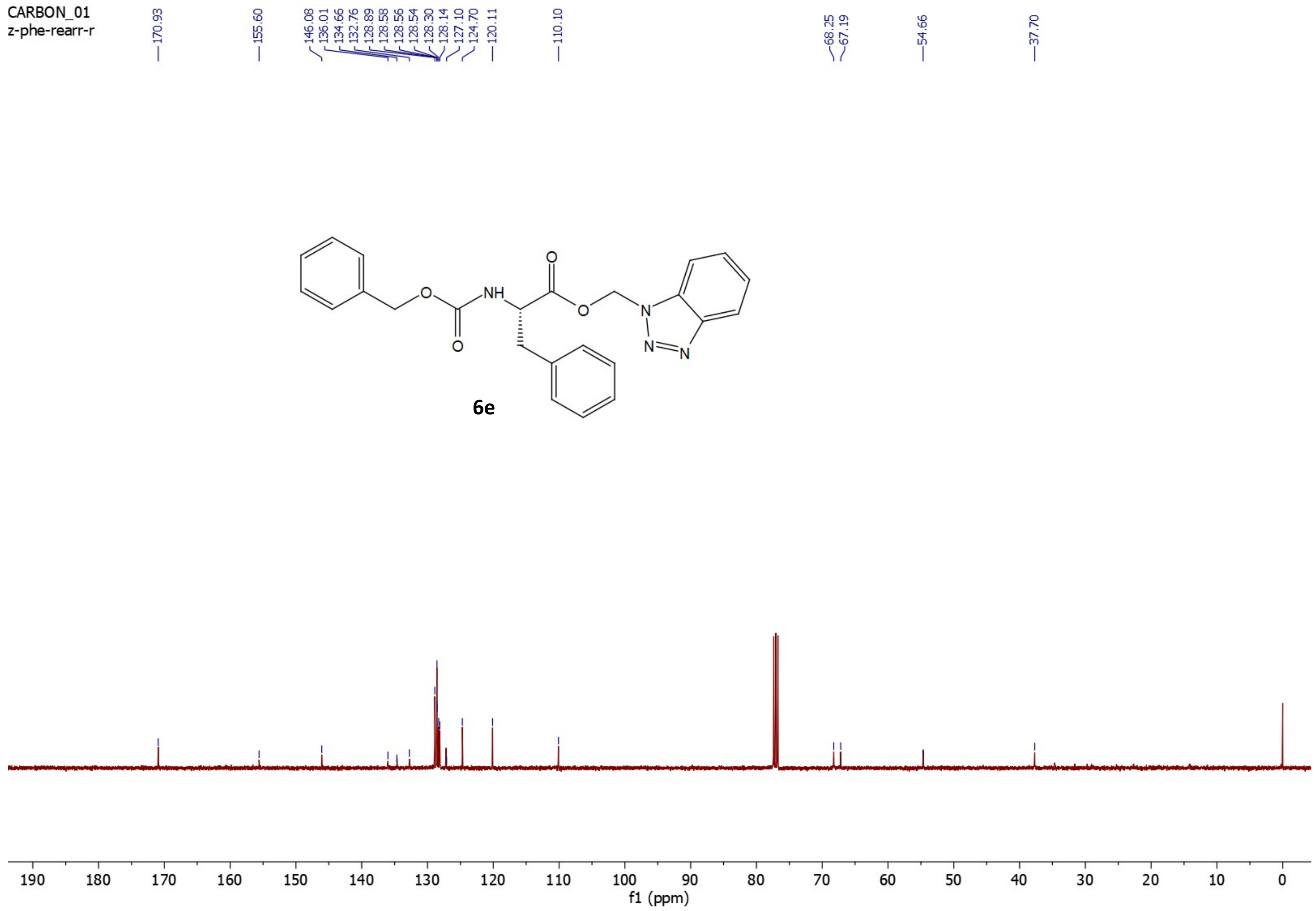

Figure S59. ^{13}C NMR of **6e**.

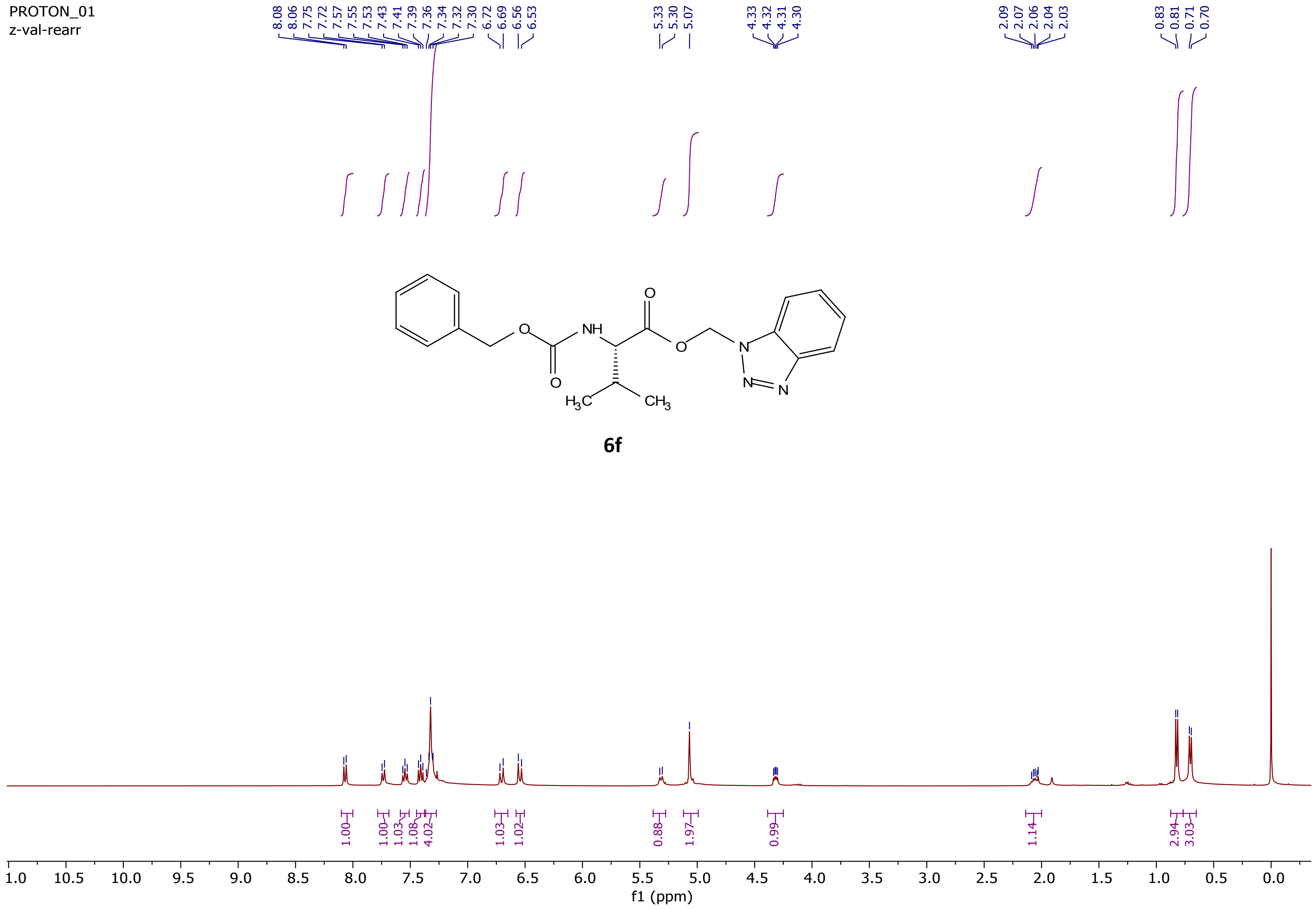

Figure S60. ^{1}H NMR of **6f**.

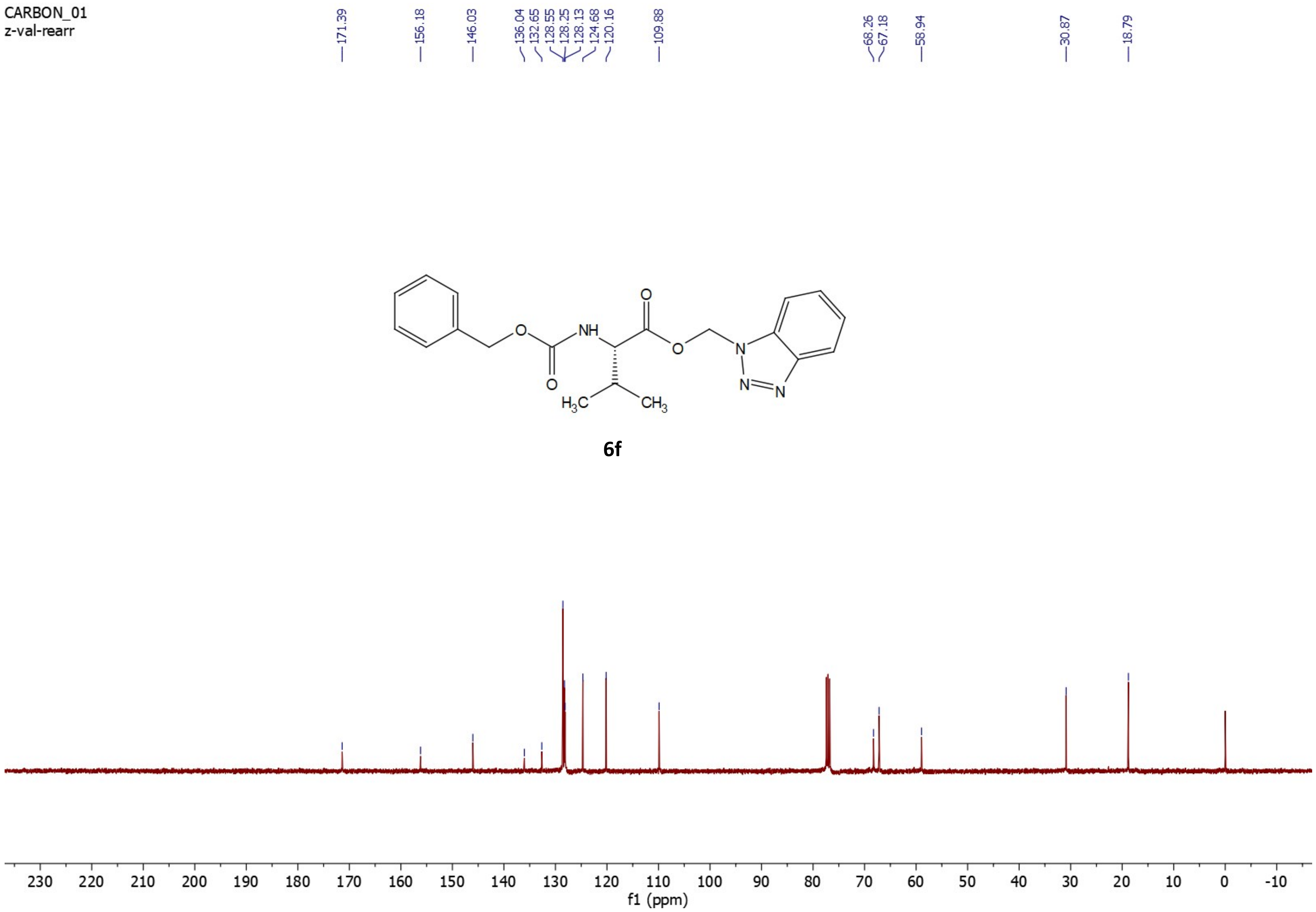

Figure S61. ^{13}C NMR of **6f**.

Crystallography

Crystals of appropriate dimension were obtained by slow evaporation of **3c**. A crystals of approximate dimensions 0.576 x 0.084 x 0.043 mm^3 was mounted on a MiTeGen cryoloop in random orientations. Preliminary examination and data collection were performed using a Bruker X8 Kappa ApexII Charge Coupled Device (CCD) Detector system single crystal X-Ray diffractometer equipped with an Oxford Cryostream LT device. All data were collected using graphite monochromated Mo Kα radiation (λ= 0.71073 Å) from a fine focus sealed tube X-Ray source. Preliminary unit cell constants were determined with a set of 36 narrow frame scans. The data sets consist of combinations of ϖ and ϕ scan frames with scan width of 0.5° and counting time of 20 seconds/frame at a crystal to detector distance of 4.0 cm. The collected frames were integrated using an orientation matrix determined from the narrow frame scans. Apex II and SAINT software packages were used for data collection and data integration.[1] Analysis of the integrated data did not show any decay. Final cell constants were determined by global refinement of reflections harvested from the complete data set. Collected data were corrected for systematic errors using SADABS based on the Laue symmetry using equivalent reflections.[1]

Crystal data and intensity data collection parameters are listed in Table S1. Structure solution and refinement were carried out using the SHELXTL- PLUS software package.[2] The structure was solved and refined successfully in the triclinic space group P-1. Full matrix least-squares refinements were carried out by minimizing $\Sigma w(F_o^2-F_c^2)^2$. The non-hydrogen atoms were refined anisotropically to convergence. All hydrogen atoms were treated using appropriate riding model (AFIX m3). The final residual values and structure refinement parameters are listed in Table S2.

Complete listings of positional and isotropic displacement coefficients for hydrogen atoms, anisotropic displacement coefficients for the non-hydrogen atoms are listed as supplementary material (Tables S2-S7, Supplementary materials). Table of calculated and observed structure factors are available in electronic format. **CCDC 1998062** contains the supplementary crystallographic data for this paper.

References:

1. Bruker Analytical X-Ray, Madison, WI, 2016

2. G.M. Sheldrick, A short history of SHELX, Acta Crystallogr. Sect. A. 64 (2008) 112–122. doi:10.1107/S0108767307043930.

Table S1. Crystal data and structure refinement for be9218.

Identification code	e9218/lt/x8	
Empirical formula	$C_{18} H_{17} F_3 N_4 O_4$	
Formula weight	410.35	
Temperature	100(2) K	
Wavelength	0.71073 Å	
Crystal system	Monoclinic	
Space group	$P2_1/n$	
Unit cell dimensions	a = 16.983(3) Å	α= 90°.
	b = 5.7953(9) Å	β= 111.694(11)°.
	c = 19.334(3) Å	γ = 90°.
Volume	1768.1(5) $Å^3$	
Z	4	
Density (calculated)	1.542 Mg/m^3	
Absorption coefficient	0.131 mm^{-1}	
F(000)	848	
Crystal size	0.576 x 0.084 x 0.043 mm^3	
Theta range for data collection	2.008 to 25.427°.	
Index ranges	$-20 \le h \le 20$, $-6 \le k \le 6$, $-23 \le l \le 23$	
Reflections collected	12882	
Independent reflections	3252 [R(int) = 0.140]	
Completeness to theta = 25.242°	100.0 %	
Absorption correction	Semi-empirical from equivalents	
Max. and min. transmission	0.9281 and 0.7637	
Refinement method	Full-matrix least-squares on F^2	
Data / restraints / parameters	3252 / 0 / 263	
Goodness-of-fit on F^2	1.008	
Final R indices [I>2sigma(I)]	R1 = 0.0655, wR2 = 0.1408	
R indices (all data)	R1 = 0.1562, wR2 = 0.1787	
Largest diff. peak and hole	0.343 and -0.388 e.$Å^{-3}$	

Table S2. Atomic coordinates (x 10^4) and equivalent isotropic displacement parameters ($Å^2$x 10^3) for be9218. U(eq) is defined as one third of the trace of the orthogonalized U^{ij} tensor.

	x	y	z	U(eq)
F(1)	8481(2)	4689(5)	5097(2)	40(1)
F(2)	9426(2)	2970(4)	4799(2)	33(1)
F(3)	8765(2)	5893(4)	4170(2)	39(1)
O(3)	7967(2)	421(5)	4277(2)	27(1)
O(4)	7552(2)	3101(5)	3383(2)	24(1)
C(17)	8669(3)	3983(8)	4523(3)	26(1)
C(18)	7989(3)	2345(7)	4016(2)	18(1)
O(1)	6206(2)	4682(5)	2026(2)	24(1)
O(2)	5076(2)	7051(5)	1616(2)	25(1)
N(1)	7177(2)	7760(6)	2998(2)	19(1)
N(2)	4100(2)	3967(7)	1359(2)	29(1)
N(3)	4404(3)	1977(7)	1735(2)	38(1)
N(4)	3815(3)	1029(7)	1937(2)	41(1)
C(1)	5729(3)	6916(8)	3570(3)	23(1)
C(2)	4999(3)	5573(8)	3311(3)	29(1)
C(3)	4901(3)	3691(9)	3705(3)	37(1)
C(4)	5531(3)	3101(9)	4366(3)	40(1)
C(5)	6269(3)	4411(9)	4633(3)	36(1)
C(6)	6364(3)	6303(8)	4236(3)	29(1)
C(7)	5826(3)	8950(7)	3126(2)	22(1)
C(8)	6273(3)	8425(7)	2589(2)	19(1)
C(9)	5872(3)	6470(8)	2056(2)	19(1)
C(10)	4612(3)	5336(8)	1074(2)	27(1)
C(11)	3288(3)	4335(8)	1316(3)	30(1)
C(12)	2687(3)	6053(9)	994(3)	36(1)
C(13)	1920(3)	5749(7)	1052(3)	23(1)
C(14)	1732(3)	3879(8)	1419(3)	32(1)
C(15)	2320(3)	2127(9)	1763(3)	35(1)
C(16)	3116(3)	2451(8)	1696(3)	31(1)

Table S3. Bond lengths [Å] and angles [°] for be9218.

F(1)-C(17)	1.329(5)
F(2)-C(17)	1.331(5)
F(3)-C(17)	1.341(5)
O(3)-C(18)	1.231(5)
O(4)-C(18)	1.252(5)
C(17)-C(18)	1.537(6)
O(1)-C(9)	1.193(5)
O(2)-C(9)	1.347(5)
O(2)-C(10)	1.448(5)
N(1)-C(8)	1.495(5)
N(1)-H(1A)	0.9100
N(1)-H(1B)	0.9100
N(1)-H(1C)	0.9100
N(2)-N(3)	1.359(5)
N(2)-C(11)	1.368(6)
N(2)-C(10)	1.428(6)
N(3)-N(4)	1.321(6)
N(4)-C(16)	1.377(6)
C(1)-C(6)	1.386(6)
C(1)-C(2)	1.392(6)
C(1)-C(7)	1.502(6)
C(2)-C(3)	1.376(7)
C(2)-H(2)	0.9500
C(3)-C(4)	1.373(7)
C(3)-H(3)	0.9500
C(4)-C(5)	1.391(7)
C(4)-H(4)	0.9500
C(5)-C(6)	1.381(7)
C(5)-H(5)	0.9500
C(6)-H(6)	0.9500
C(7)-C(8)	1.527(6)
C(7)-H(7A)	0.9900
C(7)-H(7B)	0.9900
C(8)-C(9)	1.514(6)

C(8)-H(8)	1.0000
C(10)-H(10A)	0.9900
C(10)-H(10B)	0.9900
C(11)-C(12)	1.397(6)
C(11)-C(16)	1.406(7)
C(12)-C(13)	1.360(7)
C(12)-H(12)	0.9500
C(13)-C(14)	1.394(6)
C(13)-H(13)	0.9500
C(14)-C(15)	1.406(7)
C(14)-H(14)	0.9500
C(15)-C(16)	1.418(7)
C(15)-H(15)	0.9500

F(1)-C(17)-F(2)	107.2(4)
F(1)-C(17)-F(3)	106.4(4)
F(2)-C(17)-F(3)	106.7(4)
F(1)-C(17)-C(18)	111.5(4)
F(2)-C(17)-C(18)	111.7(4)
F(3)-C(17)-C(18)	112.9(4)
O(3)-C(18)-O(4)	128.4(4)
O(3)-C(18)-C(17)	115.3(4)
O(4)-C(18)-C(17)	116.2(4)
C(9)-O(2)-C(10)	115.8(3)
C(8)-N(1)-H(1A)	109.5
C(8)-N(1)-H(1B)	109.5
H(1A)-N(1)-H(1B)	109.5
C(8)-N(1)-H(1C)	109.5
H(1A)-N(1)-H(1C)	109.5
H(1B)-N(1)-H(1C)	109.5
N(3)-N(2)-C(11)	109.9(4)
N(3)-N(2)-C(10)	120.8(4)
C(11)-N(2)-C(10)	129.3(4)
N(4)-N(3)-N(2)	109.4(4)
N(3)-N(4)-C(16)	107.7(4)
C(6)-C(1)-C(2)	118.3(5)

C(6)-C(1)-C(7)	121.3(4)
C(2)-C(1)-C(7)	120.3(4)
C(3)-C(2)-C(1)	121.2(5)
C(3)-C(2)-H(2)	119.4
C(1)-C(2)-H(2)	119.4
C(4)-C(3)-C(2)	120.1(5)
C(4)-C(3)-H(3)	120.0
C(2)-C(3)-H(3)	120.0
C(3)-C(4)-C(5)	119.6(5)
C(3)-C(4)-H(4)	120.2
C(5)-C(4)-H(4)	120.2
C(6)-C(5)-C(4)	120.1(5)
C(6)-C(5)-H(5)	119.9
C(4)-C(5)-H(5)	119.9
C(5)-C(6)-C(1)	120.6(5)
C(5)-C(6)-H(6)	119.7
C(1)-C(6)-H(6)	119.7
C(1)-C(7)-C(8)	114.8(4)
C(1)-C(7)-H(7A)	108.6
C(8)-C(7)-H(7A)	108.6
C(1)-C(7)-H(7B)	108.6
C(8)-C(7)-H(7B)	108.6
H(7A)-C(7)-H(7B)	107.5
N(1)-C(8)-C(9)	106.5(3)
N(1)-C(8)-C(7)	111.3(3)
C(9)-C(8)-C(7)	113.6(4)
N(1)-C(8)-H(8)	108.5
C(9)-C(8)-H(8)	108.5
C(7)-C(8)-H(8)	108.5
O(1)-C(9)-O(2)	124.9(4)
O(1)-C(9)-C(8)	125.5(4)
O(2)-C(9)-C(8)	109.7(4)
N(2)-C(10)-O(2)	110.4(4)
N(2)-C(10)-H(10A)	109.6
O(2)-C(10)-H(10A)	109.6
N(2)-C(10)-H(10B)	109.6

O(2)-C(10)-H(10B)	109.6
H(10A)-C(10)-H(10B)	108.1
N(2)-C(11)-C(12)	133.9(5)
N(2)-C(11)-C(16)	104.3(4)
C(12)-C(11)-C(16)	121.8(5)
C(13)-C(12)-C(11)	115.8(5)
C(13)-C(12)-H(12)	122.1
C(11)-C(12)-H(12)	122.1
C(12)-C(13)-C(14)	123.2(5)
C(12)-C(13)-H(13)	118.4
C(14)-C(13)-H(13)	118.4
C(13)-C(14)-C(15)	123.1(5)
C(13)-C(14)-H(14)	118.5
C(15)-C(14)-H(14)	118.5
C(14)-C(15)-C(16)	113.3(5)
C(14)-C(15)-H(15)	123.3
C(16)-C(15)-H(15)	123.3
N(4)-C(16)-C(11)	108.8(5)
N(4)-C(16)-C(15)	128.5(5)
C(11)-C(16)-C(15)	122.6(5)

Table S4. Anisotropic displacement parameters ($Å^2$x 10^3) for be9218. The anisotropic displacement factor exponent takes the form: $-2\pi^2[h^2 a^{*2}U^{11} + ... + 2 h k a^* b^* U^{12}]$

	U^{11}	U^{22}	U^{33}	U^{23}	U^{13}	U^{12}
F(1)	45(2)	44(2)	25(2)	-20(1)	5(1)	-4(1)
F(2)	20(1)	32(2)	34(2)	7(1)	-4(1)	5(1)
F(3)	34(2)	24(2)	41(2)	15(1)	-8(1)	-7(1)
O(3)	39(2)	18(2)	18(2)	4(2)	3(2)	0(2)
O(4)	27(2)	21(2)	15(2)	0(2)	0(1)	1(1)
C(17)	32(3)	23(3)	17(2)	2(2)	0(2)	1(2)
C(18)	24(2)	15(2)	14(2)	-5(2)	6(2)	2(2)
O(1)	25(2)	22(2)	17(2)	-4(1)	1(1)	2(2)
O(2)	22(2)	24(2)	20(2)	-7(2)	-2(1)	-2(1)
N(1)	19(2)	17(2)	13(2)	-2(2)	-2(2)	-4(2)
N(2)	26(2)	26(2)	24(2)	2(2)	-1(2)	1(2)
N(3)	44(3)	27(2)	31(2)	3(2)	1(2)	-1(2)
N(4)	48(3)	32(3)	32(3)	4(2)	1(2)	0(2)
C(1)	23(2)	29(3)	22(2)	-9(2)	13(2)	0(2)
C(2)	23(3)	35(3)	29(3)	-7(2)	10(2)	0(2)
C(3)	41(3)	38(3)	37(3)	-9(3)	20(3)	-7(3)
C(4)	61(4)	32(3)	38(3)	1(3)	31(3)	-2(3)
C(5)	36(3)	44(3)	25(3)	3(3)	7(2)	1(3)
C(6)	31(3)	34(3)	21(3)	0(2)	6(2)	-6(2)
C(7)	26(2)	23(3)	13(2)	-3(2)	2(2)	1(2)
C(8)	19(2)	23(2)	12(2)	-4(2)	0(2)	2(2)
C(9)	21(2)	21(3)	12(2)	-1(2)	3(2)	-6(2)
C(10)	32(3)	24(3)	19(2)	-4(2)	0(2)	-5(2)
C(11)	33(3)	26(3)	26(3)	0(2)	3(2)	0(2)
C(12)	38(3)	30(3)	29(3)	7(2)	2(2)	7(3)
C(13)	25(3)	18(3)	30(3)	3(2)	15(2)	5(2)
C(14)	35(3)	28(3)	35(3)	-9(3)	16(2)	-3(2)
C(15)	36(3)	30(3)	40(3)	-13(3)	15(3)	-5(3)
C(16)	35(3)	28(3)	23(3)	-2(2)	1(2)	1(2)

Table S5. Hydrogen coordinates (x 10^4) and isotropic displacement parameters ($Å^2$x 10^3) for be9218.

	x	y	z	U(eq)
H(1A)	7474	7946	2694	22
H(1B)	7204	6258	3142	22
H(1C)	7405	8673	3407	22
H(2)	4560	5961	2853	35
H(3)	4396	2800	3521	45
H(4)	5464	1805	4640	48
H(5)	6709	4002	5088	44
H(6)	6869	7194	4422	35
H(7A)	6148	10167	3476	27
H(7B)	5256	9576	2838	27
H(8)	6260	9846	2291	23
H(10A)	4246	6112	610	33
H(10B)	5015	4330	954	33
H(12)	2807	7353	750	43
H(13)	1490	6866	833	28
H(14)	1181	3783	1437	38
H(15)	2195	854	2014	42

Table S6. Torsion angles [°] for be9218.

F(1)-C(17)-C(18)-O(3)	-70.9(5)
F(2)-C(17)-C(18)-O(3)	49.0(6)
F(3)-C(17)-C(18)-O(3)	169.3(4)
F(1)-C(17)-C(18)-O(4)	111.6(5)
F(2)-C(17)-C(18)-O(4)	-128.5(4)
F(3)-C(17)-C(18)-O(4)	-8.1(6)
C(11)-N(2)-N(3)-N(4)	0.2(5)
C(10)-N(2)-N(3)-N(4)	-179.8(4)
N(2)-N(3)-N(4)-C(16)	-0.9(5)
C(6)-C(1)-C(2)-C(3)	0.6(7)
C(7)-C(1)-C(2)-C(3)	179.4(4)
C(1)-C(2)-C(3)-C(4)	-0.3(8)
C(2)-C(3)-C(4)-C(5)	-0.2(8)
C(3)-C(4)-C(5)-C(6)	0.4(8)
C(4)-C(5)-C(6)-C(1)	-0.2(8)
C(2)-C(1)-C(6)-C(5)	-0.3(7)
C(7)-C(1)-C(6)-C(5)	-179.1(5)
C(6)-C(1)-C(7)-C(8)	86.4(5)
C(2)-C(1)-C(7)-C(8)	-92.4(5)
C(1)-C(7)-C(8)-N(1)	-65.6(5)
C(1)-C(7)-C(8)-C(9)	54.5(5)
C(10)-O(2)-C(9)-O(1)	0.0(6)
C(10)-O(2)-C(9)-C(8)	179.9(4)
N(1)-C(8)-C(9)-O(1)	5.8(6)
C(7)-C(8)-C(9)-O(1)	-117.0(5)
N(1)-C(8)-C(9)-O(2)	-174.1(4)
C(7)-C(8)-C(9)-O(2)	63.1(5)
N(3)-N(2)-C(10)-O(2)	-91.4(5)
C(11)-N(2)-C(10)-O(2)	88.7(5)
C(9)-O(2)-C(10)-N(2)	95.3(4)
N(3)-N(2)-C(11)-C(12)	-179.3(5)
C(10)-N(2)-C(11)-C(12)	0.7(9)
N(3)-N(2)-C(11)-C(16)	0.6(5)
C(10)-N(2)-C(11)-C(16)	-179.5(4)

N(2)-C(11)-C(12)-C(13)	177.8(5)
C(16)-C(11)-C(12)-C(13)	-2.0(7)
C(11)-C(12)-C(13)-C(14)	1.2(7)
C(12)-C(13)-C(14)-C(15)	0.0(8)
C(13)-C(14)-C(15)-C(16)	-0.3(7)
N(3)-N(4)-C(16)-C(11)	1.2(5)
N(3)-N(4)-C(16)-C(15)	177.9(5)
N(2)-C(11)-C(16)-N(4)	-1.1(5)
C(12)-C(11)-C(16)-N(4)	178.8(4)
N(2)-C(11)-C(16)-C(15)	-178.0(5)
C(12)-C(11)-C(16)-C(15)	1.9(7)
C(14)-C(15)-C(16)-N(4)	-176.9(5)
C(14)-C(15)-C(16)-C(11)	-0.6(7)

Table S7. Hydrogen bonds for be9218 [Å and °].

D-H...A	d(D-H)	d(H...A)	d(D...A)	<(DHA)
N(1)-H(1A)...O(4)#1	0.91	2.07	2.879(5)	147.5
N(1)-H(1A)...O(1)#1	0.91	2.33	2.978(5)	128.0
N(1)-H(1B)...O(4)	0.91	1.93	2.811(4)	163.5
N(1)-H(1C)...O(3)#2	0.91	1.89	2.800(4)	175.0
N(1)-H(1C)...O(4)#2	0.91	2.58	3.192(4)	125.1
C(7)-H(7B)...N(3)#2	0.99	2.51	3.368(6)	144.9
C(7)-H(7B)...N(4)#2	0.99	2.57	3.549(6)	172.4
C(10)-H(10B)...F(2)#3	0.99	2.47	3.045(5)	116.4
C(12)-H(12)...F(1)#4	0.95	2.63	3.556(6)	165.2

Symmetry transformations used to generate equivalent atoms:

#1 -x+3/2,y+1/2,-z+1/2 #2 x,y+1,z #3 x-1/2,-y+1/2,z-1/2

#4 x-1/2,-y+3/2,z-1/2

Projection view with 50% probability ellipsoids:

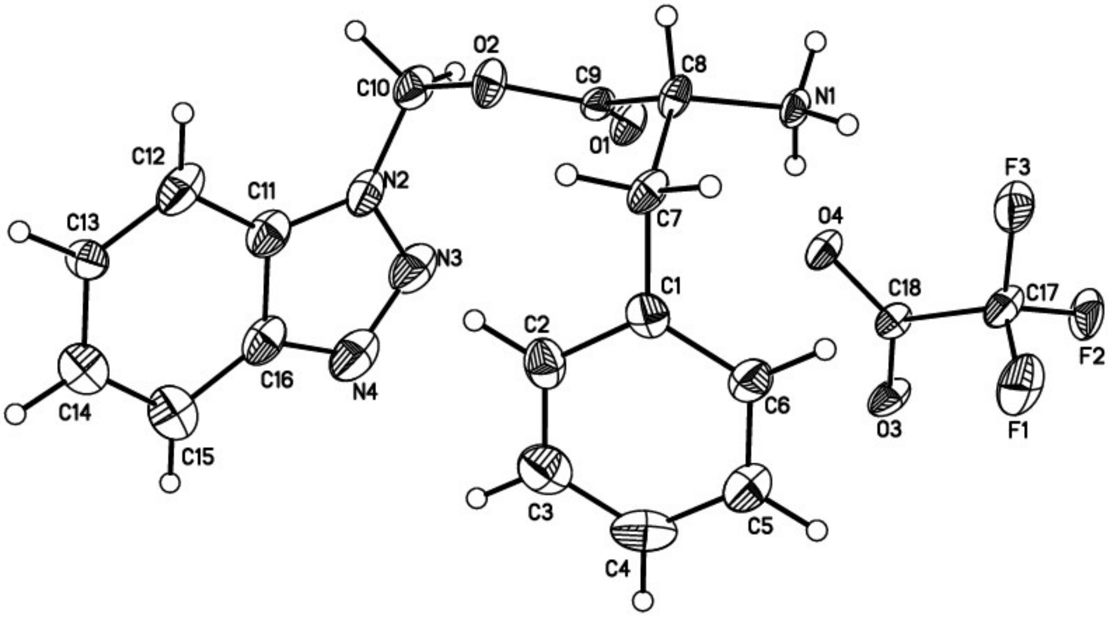

Supplement: RA-011-D0RA10413B-s001 [file RA-011-D0RA10413B-s001.pdf]
